# Improving drought tolerance in some wheat genotypes with foliar application of silicon nanoparticles in Al-Dawadmi, Saudi Arabia

**DOI:** 10.7717/peerj.20823

**Published:** 2026-02-24

**Authors:** Mesfer M. Alqahtani

**Affiliations:** Department of Biological Sciences, Faculty of Science and Humanities, Shaqra University, Shaqra, Saudi Arabia

**Keywords:** Drought stress, Genotypes, Grain yield, SiNP, Wheat

## Abstract

Water stress, even at light or moderate levels, can negatively affect wheat physiology by altering gas exchange and photosynthetic pigments, thereby restricting growth, while severe stress further amplifies these effects and leads to more pronounced yield reductions. Foliar application of silicon nanoparticles (SiNPs) may help mitigate some of these detrimental effects on wheat’s physiological and agronomic traits, as evaluated over two growing seasons at a single location. To test this hypothesis, two field experiments were conducted during the 2022/2023 and 2023/2024 winter seasons at the Experimental Farm in Al-Dawadmi, Saudi Arabia. The study evaluated the response of eight wheat genotypes to foliar-applied SiNPs under three irrigation regimes representing full, moderate, and severe water stress conditions.

Agronomic and physiological characteristics of wheat were negatively affected by moderate and severe water stress. In comparison to the untreated control, the application of 100 and 200 mg L^−1^ of SiNPs, particularly at the higher concentration, alleviated some of the negative effects of water stress on various physiological and yield traits, with Giza 171, SOKOLL, and Giza 168 showing the most pronounced improvements. In the same context, under severe water stress, SiNP application improved net photosynthesis (up to 57.19%), transpiration rate (up to 36.20%), stomatal conductance (up to 31.34%), intercellular CO_2_ concentration (up to 34.21%), water use efficiency (up to 15.69%), relative water content (up to 38.16%), chlorophyll content (up to 37.51%), spikes per plant (up to 50.80%), grains per spike (up to 56.52%), and grain yield per hectare (up to 50.33%). These results demonstrate the potential of SiNP application to improve drought tolerance in wheat genotypes under the specific agro-ecological conditions of Al-Dawadmi and highlight genotype-specific responses, particularly in Giza 171 and SOKOLL. While confirming previous findings on the role of silicon in mitigating drought stress, this study provides novel insights into the differential responses of wheat genotypes in an arid environment. However, further multi-location and long-term trials are needed to validate these effects and to assess environmental safety, soil accumulation, and practical feasibility.

## Introduction

Drought stress has a profound influence on cereal crop productivity, particularly wheat, by altering physiological and biochemical processes that ultimately determine yield (Wasaya et al., 2023). Global warming and changed precipitation patterns can produce water shortage, leading to physiological drought even when soil moisture is adequate, altering plant water uptake (Abdelaal et al., 2021). Drought affects plants differently depending on their developmental stage, stress severity and duration, genetic makeup, and environmental interactions (Begna, 2020). Plants respond to drought by employing various mechanisms to conserve water while maintaining essential metabolic activities (Kapoor et al., 2020). Reduced rainfall at critical growth stages may delay sowing, increase the risk of terminal drought, and significantly reduce grain yield (Saradadevi, Palta & Siddique, 2017). Water shortage reduces yield by 29.4% to 61.8% depending on genotype and environment, while negatively affecting physiological characteristics such as chlorophyll content and relative water content (Kamara et al., 2022; Hussein et al., 2024). Drought conditions also reduce chlorophyll content, photosynthetic efficiency, and grain yield, while proline and antioxidant enzyme activities increase as stress responses (Kamara et al., 2022).

Silicon is recognized as an essential element for enhancing plant tolerance to abiotic stresses, particularly drought (Sakr, 2018; Abou-Shlell, El-Emary & Khalifa, 2020). Exogenous silicon application, including foliar sprays, has been shown to improve drought tolerance in wheat by enhancing chlorophyll content, gas exchange, antioxidant activity, biomass accumulation, and grain yield (Bukhari et al., 2015; Maghsoudi et al., 2019; Aurangzaib et al., 2021; Khalid et al., 2022). Despite extensive studies on silicon and drought, limited information exists regarding the effects of foliar-applied silicon nanoparticles (SiNPs) on the physiological, biochemical, and agronomic performance of wheat genotypes in the arid agro-ecological conditions of Al-Dawadmi Governorate, Saudi Arabia. This represents a clear knowledge gap that needs to be addressed. We hypothesized that foliar application of SiNPs would improve wheat tolerance to water deficit by enhancing chlorophyll content, gas exchange, water-use efficiency, and ultimately grain yield, with the magnitude of response varying among different wheat genotypes.

Recent studies further justify this knowledge gap and the importance of this research. While nano-silicon fertilizers and foliar-applied SiNPs have been shown to significantly improve drought tolerance, yield, water use efficiency, and stress gene expression in wheat under various drought conditions, most research has been conducted outside the Arabian Peninsula and under different agro-ecological settings (Ahmadian, Jalilian & Pirzad, 2021; Boora et al., 2023a; Boora et al., 2023b; Raza et al., 2023; Ning et al., 2023; Davoudi et al., 2024; Zulfiqar et al., 2024; Direkvandy et al., 2024). There is a lack of data on the effectiveness of SiNPs in the unique arid conditions of Al-Dawadmi, especially regarding their interaction with local wheat genotypes and the timing of application during critical growth stages (Boora et al., 2023a; Boora et al., 2023b; Raza et al., 2023; Ning et al., 2023; Zulfiqar et al., 2024; Davoudi et al., 2024; Direkvandy et al., 2024). This highlights the urgent need for region-specific studies to develop targeted, sustainable strategies for wheat production under increasing drought stress due to climate change.

Therefore, the aim of this study was to evaluate the potential of foliar-applied SiNPs in mitigating drought stress effects on physiological traits, photosynthetic pigments, water relations, growth, and grain yield of selected wheat genotypes under the specific conditions of Al-Dawadmi Governorate.

## Materials and Methods

### Site description

**Figure 1:**
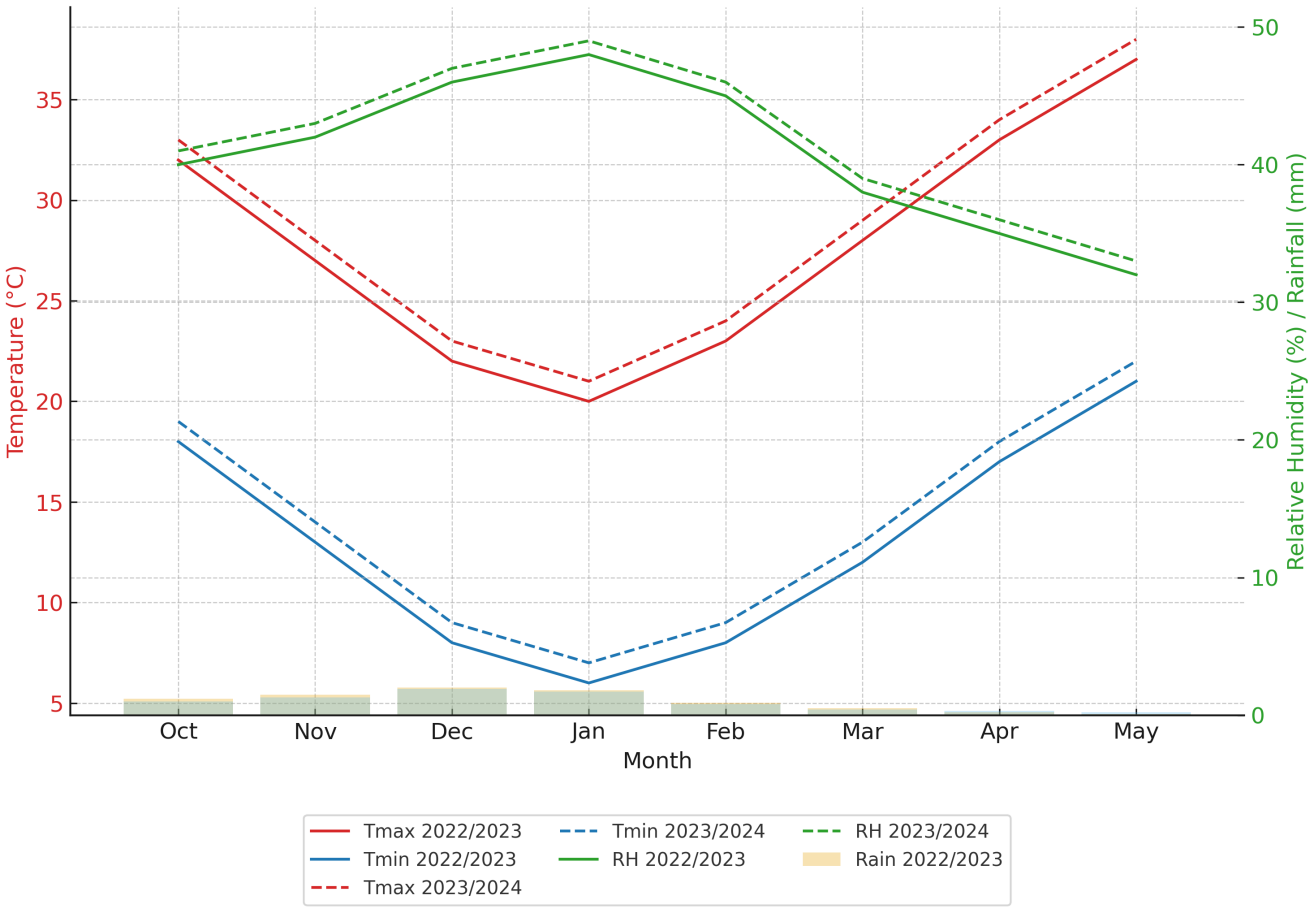
Monthly climatic conditions at Al-Dawadmi experimental site during the 2022/2023 and 2023/2024 wheat growing seasons.

Two field trials were conducted at the Experimental Farm in Al-Dawadmi Governorate, Kingdom of Saudi Arabia, during the winter seasons of 2022/2023 and 2023/2024. The experimental farm in Saudi Arabia, located at 24°31′0.12″N latitude, 44°25′5.52″E Longitude, and 933.75 m above sea level, experienced an average relative humidity of 41.28%, a temperature of 32.92 °C during the day and 7.20 °C at night, and an average precipitation of 0.69 mm/day (Fig. 1). Moreover, the Al-Dawadmi site is uniquely characterized by extreme aridity and highly alkaline, sandy-loam soils with low water-holding capacity, underscoring the need for localized field studies in this region.

Soil samples were collected before sowing to identify soil properties; the samples were air-dried, ground, sieved, and stored for further analysis. The soil experiment involved a comprehensive analysis of physical and chemical parameters using Jackson’s (1973) methods (Table 1). In addition, analytical procedures were verified and aligned with the updated soil testing protocols described by Jones (2001) and Carter & Gregorich (2008) to ensure measurement accuracy and comparability with recent studies, while maize was the preceding crop in both seasons.

#### Plant materials

The research involved examining the plant material of bread wheat, consisting of eight genotypes. These genotypes included six Egyptian cultivars, Giza 171, Sakha 95, Misr 3, Gemmeiza-9, Giza-168, and Sids-14, two CIMMYT genotypes, SOKOLL, and 18 SAWYT 19/20 (Table 2).

### Experimental design and treatments

Because irrigation treatments are more difficult to change frequently, three adjacent trials, each representing a separate irrigation regime, were conducted in a randomized complete block design with a split-plot scheme and three replications. The first treatment (I1) received the recommended seasonal irrigation amount for Al-Dawadmi Governorate (≈7,000 m^3^ ha^−1^), distributed equally over ten irrigations (≈700 m^3^ ha^−1^ per irrigation) throughout the growing season according to crop requirements and prevailing weather conditions. The second (I2) and third (I3) treatments received 2/3 (≈470 m^3^ ha^−1^ per irrigation; total ≈4,700 m^3^ ha^−1^) and 1/3 (≈235 m^3^ ha^−1^ per irrigation; total ≈2,350 m^3^ ha^−1^) of the I1 amount, respectively, while maintaining the same number (ten) and timing of irrigations as the control, starting 30 days after sowing. Within each irrigation trial, foliar applications of silicon nanoparticles (SiNPs) in the form of silicon dioxide (SiO_2_) were applied to the main plots at three concentrations: an untreated control (SiNPs0), 100 mg L^−1^ (SiNPs100), and 200 mg L^−1^ (SiNPs200), while the eight wheat genotypes were arranged in the sub-plots. Foliar sprays of SiNPs were applied twice: the first at 30 days after sowing (tillering stage) and the second at 65 days after sowing (booting stage) using a hand sprayer until full leaf wetness. This design allowed precise assessment of SiNP effects and genotype × SiNP interactions under each water regime, while maintaining operational feasibility and statistical precision.

**Table 1:** Physicals and chemicals analyses of experimental sites at 30 cm depth of soil.

Seasons	Available			pH	EC mmh/v	Clay %	Silt %	Fine sand %	Texture
	N	P	K						
2022/2023	8.15	2.42	34	7.16	1.23	19.28	35.24	45.48	Loam
2023/2024	8.19	2.56	44	7.18	1.28	22.32	34.51	43.17	Loam

**Table 2:** Pedigree, selection history and origin of the eight bread wheat genotypes.

Source	Pedigree and selection history	Origin
Giza 171	Sakha 93 /Gemmieza 9. GZ2003 –101-1GZ - 4GZ –1GZ - 2GZ - 0GZ.	Egypt
Sakha 95	PASTOR//SITE/MO/3/CHEN/AEGILOPS SQUARROSA (TAUS) // BCN /4/ WBLL1 (CMSA01Y00158S-040P0Y-040M-030ZTM-040SY-26M-0Y-0SY-0S).	Egypt
Misr 3	Rohf 07*2/Kiriti CGSS 05 B00123T-099T-0PY-099M-099NJ-6WGY-0B-0BGY-0GZ.	Egypt
Gemmeiza-9	Ald ”S”/Huac ”S”//CMH74A. 630/5x CGM4583-5GM-1GM-0GM	Egypt
Giza 168	MRL/BUC//Seri. CM93046-8M-0Y-0M-2Y-0B-0GZ	Egypt
Sids-14	BOW”s”/vee”s”//BOW”S”/TSI/BANI SEWEF1 AND SD293-1SD-2SD-4SD-0SD	Egypt
SOKOLL	Sokoll CMSS97M00316S-0P20M-0P20Y-43M-010Y	CIMMYT
18 SAWYT 19/20	MUU/KBIRD//2*KACHU/KIRITATI CMSS12Y01082T-099TOPM-099Y-099M-099NJ-099NJ-6Y-0WGY	CIMMYT

The nanoparticles had sizes ranging from 20 to 30 nm with 99% purity. The spraying solution was prepared by dispersing the nanoparticles in deionized water using an ultrasonic water bath (300 W, 40 kHz) combined with magnetic stirring for 30 min, following Dai et al. (2018).

The eight wheat genotypes evaluated in the sub-plots included six Egyptian cultivars (Giza 171, Sakha 95, Misr 3, Gemmeiza 9, Giza 168, and Sids 14) and two CIMMYT genotypes (SOKOLL and 18 SAWYT 19/20).

### Planting procedure

A wide border was surrounded to minimize underground water permeability, ensuring optimal growth of wheat genotypes in the Al-Dawadmi Governorate. In the fourth week of December, wheat genotypes were grown in a randomized complete block design with a split-plot arrangement of three replications under three irrigation regime treatments and three foliar applications of silicon nanoparticles. Each plot had four rows, two m long and 20 cm apart. Thus, the experiment involved 1.6 m^2^ plots with seeds drilled in rows at a rate of 400 seeds per m^−2^. To ensure uniform germination and early growth, all plots were initially irrigated with the full irrigation level (control) to establish the crop. Irrigation treatments were applied 30 days after sowing using a sprinkler irrigation system designed and installed to ensure uniform water distribution across the plots. The system was equipped with properly spaced sprinkler heads operating at constant pressure, and water flow meters were used to monitor the actual amounts of water delivered. The scheduled irrigations were distributed over the growing season according to crop requirements. All other cultural practices were implemented according to the locally recommended guidelines for wheat production in the Al-Dawadmi Region, including land preparation, sowing dates, fertilization schedules, weed and pest management, and harvesting procedures, to ensure optimal wheat cultivation under regional conditions.

### Data collection

#### Leaf gas exchange traits

Five wheat plants were randomly selected 75 days after sowing to measure gas exchange characteristics of their leaves, including photosynthetic rate (µmol CO_2_/m^2^/s), stomatal conductance (mol/m^2^/s), intracellular CO_2_ concentration (µmol/mol), and transpiration rate (mmol H_2_O/m^2^/s). These measurements provided insights into the physiological functions of wheat plants. The third leaf on the upper part of each plant was measured between 9 AM and 11 AM on sunny days using a portable LI-6400XT photosynthesis system (Li-Cor Inc, 1998). The best management was achieved using a battery to maintain optimal environmental conditions in the leaf chambers.

### Plant water potential

Water use efficiency (WUE) is a measure of plant growth efficiency, calculated by dividing the rate of photosynthetic (Pn) by transpiration (TR). Leaf relative water content (RWC) was calculated as ((fresh mass–dry mass)/(turgid mass–dry mass)) × 100, following Farouk, Elhindi & Alotaibi (2020).

### Photosynthetic pigments

Photosynthetic pigments, such as chlorophyll a, chlorophyll b, carotenoids, and chlorophyll a + b, are essential components of the plant’s growth and development. These pigments are measured in fresh wheat leaves at 75 days after sowing using spectrophotometric analysis, following Moran’s (1982) recommended method. The concentrations of these pigments are measured as mg g^−1^ fresh weight.

### Plant growth measurements

For the purpose of measuring growth traits, ten guarded plants were chosen at random from each plot of the three replications. Several characteristics were measured during harvest, including as leaf area (cm^2^), number of leaves and tillers per plant, shoot length (cm), and shoot dry weight (g). LI-COR (LI-3000; LI-CORincoln, NE, USA) was used to measure the leaf area, and following oven drying, the dry weight (g) was noted at 70 °C.

### Yield measurement

Ten plants were randomly selected at harvest maturity in both seasons, focusing on estimating spike length, spikes number per plant, and grains number per spike from each replicate of each treatment. The grain and straw yields were estimated from the three central rows to convert each plot into ton hectare^−1^, for eliminating the border effect and ensuring accurate results. Additionally, grain yield per plant was recorded to validate the hectare-based yield calculations and to ensure realistic estimation across treatments.

#### Statistical analysis

The collected data was analyzed using the “MSTATC” microcomputer program (MSTAT-C, 1991) and verified, with all final results produced using IBM SPSS Statistics v26 (Armonk, NY, USA). The experiment consisted of three adjacent irrigation trials, each with three replications, arranged in a randomized complete block design with a split-plot structure. Within each irrigation trial, SiNPs foliar application was assigned to the main plots, and wheat genotypes were allocated to the subplots. Treatment means for irrigation regimes, SiNPs foliar application, and wheat genotypes, including their interactions, were compared using Duncan’s multiple range test (Duncan, 1955) at a 5% level of probability, which is widely used in agricultural experiments to reliably distinguish among treatment means. Before performing ANOVA, data were tested for normality using the Shapiro–Wilk test and for homogeneity of variances using Levene’s test to verify that the assumptions of parametric analysis were met. These tests were conducted on the residuals, and these diagnostic checks confirmed that the residuals followed a normal distribution and that variances were homogeneous across treatments, ensuring the validity of the ANOVA model (Gomez & Gomez, 1984; Ghasemi & Zahediasl, 2012; Mohd Razali & Bee Wah, 2011; Blanca et al., 2017). All figures were drawn using standard error (SE) bars to clearly illustrate the variability and significance among treatments.

## Results

### Analysis of variance

The analysis of variance revealed highly significant effects of the studied factors, irrigation regimes, silicon nanoparticles (SiNPs), and genotypic variation, on the majority of physiological, biochemical, growth, and yield-related traits across both seasons (Tables 3–7). Irrigation regime consistently exerted a strong influence on net photosynthesis, transpiration rate, stomatal conductance, water use efficiency, and intercellular CO_2_ concentration, indicating the pivotal role of water availability in regulating gas exchange and overall plant performance. Similarly, the application of SiNPs significantly enhanced photosynthetic attributes, water relations, and pigment composition, suggesting that silicon supplementation mitigated the adverse effects of water deficit and improved physiological resilience.

Genotypic differences were pronounced for almost all evaluated traits, including photosynthetic efficiency, chlorophyll fractions, shoot growth, and yield components, underscoring the genetic diversity in adaptive responses. The significant interaction effects between irrigation regimes and SiNPs, as well as between irrigation and genotypes, highlight the importance of combined management practices and genetic selection in optimizing crop performance under varying water conditions. Moreover, the three-way interaction (I × SiNPs × G) was significant for several traits, reflecting the complexity of plant responses when both genetic and environmental factors coincide with nanonutrient application.

In terms of growth and yield parameters, irrigation and SiNPs markedly influenced shoot length, leaf area, tillering, and biomass accumulation, which were positively translated into higher spike length, spike number, grain number per spike, and ultimately grain yield. The consistent significance of genotype main effects and interactions across seasons further validates the stability and reliability of these responses. Collectively, these findings confirm that integrating appropriate irrigation strategies with SiNPs application can enhance physiological efficiency, maintain higher relative water content, and promote pigment stability, thereby supporting superior growth and yield potential in drought-challenged environments.

**Table 3:** Analysis of variance for net photosynthesis, transpiration rate, and stomatal conductance across two growing seasons.

S.O.V.	df	Net photosynthesis		Transpiration rate		Stomatal conductance	
		1st season	2nd season	1st season	2nd season	1st season	2nd season
Irrigation regime (I)	2	33.6201^**^	32.5320^**^	0.6449^**^	0.6574^**^	0.0059^**^	0.0060^**^
Reps within irrigation	6	0.2088	0.1716	0.0110	0.0070	0.0001	0.0001
Silicon nanoparticles (SiNPs)	2	19.4756^**^	20.6229^**^	0.3731^**^	0.4166^**^	0.0034^**^	0.0038^**^
I X SiNPs	4	0.9740^**^	0.7284^**^	0.0186^**^	0.0147^**^	0.0002^**^	0.0001^**^
Pooled error A	12	0.0593	0.0573	0.0032	0.0023	0.0001	0.0001
Genotypes (G)	7	52.3842^**^	56.2464^**^	1.0043^**^	1.1364^**^	0.0092^**^	0.0104^**^
I X G	14	3.2515^**^	3.1970^**^	0.0623^**^	0.0646^**^	0.0006^**^	0.0006^**^
SiNPs X G	14	0.9691^**^	1.0074^**^	0.0186^**^	0.0204^**^	0.0002^**^	0.0002^**^
I x SiNPs x G	28	0.8955^**^	1.0423^**^	0.0172^**^	0.0211^**^	0.0002^**^	0.0002^**^
Pooled Error B	126	0.0637	0.0602	0.0034	0.0025	0.0001	0.0001

**Notes.**

*, ** Significant at 0.05 and 0.01 probability level, respectively.

**Table 4:** Analysis of variance for water use efficiency, intercellular CO_2_ concentration, and relative water content in both seasons.

S.O.V.	df	Water use efficiency		Intercellular CO2		Relative water content	
		1st season	2nd season	1st season	2nd season	1st season	2nd season
Irrigation regime (I)	2	0.6980^**^	0.3770^**^	5,502.32^**^	5,609.23^**^	733.5617^**^	619.1426^**^
Reps within irrigation	6	0.0013	0.0010	93.65	59.992	12.4851	6.6219
Silicon nanoparticles (SiNPs)	2	0.3523^**^	0.2106^**^	3,183.57^**^	3,554.36^**^	424.4296^**^	392.3279^**^
I X SiNPs	4	0.0202^**^	0.0087^**^	159.02^**^	125.20^**^	21.2002^**^	13.8199^**^
Pooled error A	12	0.0003	0.0003	27.72	19.82	3.6959	2.1882
Genotypes (G)	7	0.9796^**^	0.5960^**^	8,569.10^**^	9,695.65^**^	1,142.4197^**^	1,070.1992^**^
I X G	14	0.0558^**^	0.0294^**^	531.87^**^	551.29^**^	70.9088^**^	60.8515^**^
SiNPs X G	14	0.0232^**^	0.0135^**^	158.41^**^	173.64^**^	21.1184^**^	19.1666^**^
I x SiNPs x G	28	0.0170^**^	0.0113^**^	146.42^**^	179.69^**^	19.5205^**^	19.8344^**^
Pooled Error B	126	0.0003	0.0003	29.0348	20.9654	3.8709	2.3141

**Notes.**

*, ** Significant at 0.05 and 0.01 probability level, respectively.

**Table 5:** Analysis of variance for chlorophyll a, chlorophyll b, carotenoids, and total photosynthetic pigments across seasons.

S.O.V.	df	Chlorophyll a		Chlorophyll b		Carotenoid		Total photosynthetic pigments	
		1st season	2nd season	1st season	2nd season	1st season	2nd season	1st season	2nd season
Irrigation regime (I)	2	0.8593^**^	0.8760^**^	0.0616^**^	0.0628^**^	0.0194^**^	0.0198^**^	0.9564^**^	0.9749^**^
Reps within irrigation	6	0.0146	0.0094	0.0010	0.0007	0.0003	0.0002	0.016	0.010
Silicon nanoparticles (SiNPs)	2	0.4972^**^	0.5551^**^	0.0357^**^	0.0398^**^	0.0112^**^	0.0125^**^	0.5533^**^	0.6178^**^
I X SiNPs	4	0.0248^**^	0.0196^**^	0.0018^**^	0.0014^**^	0.0006^**^	0.0004^**^	0.0276^**^	0.0218^**^
Pooled error A	12	0.0043	0.0031	0.0003	0.0002	0.0001	0.0001	0.005	0.003
Genotypes (G)	7	1.3383^**^	1.5143^**^	0.0960^**^	0.1086^**^	0.0302^**^	0.0342^**^	1.4894^**^	1.6852^**^
I X G	14	0.0831^**^	0.0861^**^	0.0060^**^	0.0062^**^	0.0019^**^	0.0019^**^	0.0924^**^	0.0958^**^
SiNPs X G	14	0.0247^**^	0.0271^**^	0.0018^**^	0.0019^**^	0.0006^**^	0.0006^**^	0.0275^**^	0.030^**^
I x SiNPs x G	28	0.0229^**^	0.0281^**^	0.0016^**^	0.0020^**^	0.0005^**^	0.0006^**^	0.0254^**^	0.0312^**^
Pooled Error B	126	0.0045	0.0033	0.0003	0.0002	0.0001	0.0001	0.005	0.004

**Notes.**

*, ** Significant at 0.05 and 0.01 probability level, respectively.

**Table 6:** Analysis of variance for shoot length, leaf area per plant, number of tillers per plant, number of leaves per plant, and shoot dry weight in both seasons.

S.O.V.	Df	Shoot length		Leaf area per plant		No. of tillers per plant		No. of leaves per plant		Shoot DW per plant	
		1st season	2nd season	1st season	2nd season	1st season	2nd season	1st season	2nd season	1st season	2nd season
Irrigation regime (I)	2	1,735.3535^**^	1,805.1328^**^	11,946.1572^**^	12,178.2667^**^	3.0625^**^	3.1220^**^	29.7002^**^	30.2772^**^	8.54^**^	8.70^**^
Reps within irrigation	6	10.7773	15.1473	203.3215	130.2502	0.0521	0.0334	0.5055	0.3238	0.15	0.093
Silicon nanoparticles (SiNPs)	2	1,005.2649^**^	1,144.5758^**^	6,911.8963^**^	7,716.9196^**^	1.7719^**^	1.9783^**^	17.1841^**^	19.1856^**^	4.94^**^	5.51^**^
I X SiNPs	4	50.2765^**^	40.4595^**^	345.2486^**^	271.8321^**^	0.0885^**^	0.0697^**^	0.8583^**^	0.6758^**^	0.25^**^	0.19^**^
Pooled error A	12	3.0591	5.1630	60.1888	43.0406	0.0154	0.0110	0.1496	0.1070	0.04	0.03
Genotypes (G)	7	2,703.8933^**^	3,121.3088^**^	18,604.4679^**^	21,050.3532^**^	4.7693^**^	5.3964^**^	46.2538^**^	52.3347^**^	13.29^**^	15.04^**^
I X G	14	167.8292^**^	177.4144^**^	1,154.7595^**^	1,196.9223^**^	0.2960^**^	0.3068^**^	2.8709^**^	2.9758^**^	0.83^**^	0.86^**^
SiNPs X G	14	50.0203^**^	55.8840^**^	343.9167^**^	376.9979^**^	0.0882^**^	0.0966^**^	0.8550^**^	0.9373^**^	0.25^**^	0.27^**^
I x SiNPs x G	28	46.2236^**^	57.8543^**^	317.8943^**^	390.1342^**^	0.0815^**^	0.1000^**^	0.7903^**^	0.9699^**^	0.23^**^	0.28^**^
Pooled Error B	126	3.2880	5.3553	63.0377	45.5183	0.0162	0.0117	0.1567	0.1132	0.0450	0.0325

**Notes.**

*, ** Significant at 0.05 and 0.01 probability level, respectively.

**Table 7:** Analysis of variance for spike length, number of spikes per plant, number of grains per spike, straw yield, and grain yield per hectare across two seasons.

S.O.V.	df	Spike length		No. of spikes per plant		No. of grains per spike		Straw yield per hectare		Grain yield per hectare	
		1st season	2nd season	1st season	2nd season	1st season	2nd season	1st season	2nd season	1st season	2nd season
Irrigation regime (I)	2	23.47^**^	23.92^**^	33.09^**^	33.73^**^	356.36^**^	363.28^**^	29.70^**^	30.28^**^	9.59^**^	9.78^**^
Reps within irrigation	6	0.3994	0.2559	0.563	0.361	6.07	3.89	0.51	0.32	0.16	0.10
Silicon nanoparticles (SiNPs)	2	13.58^**^	15.16^**^	19.15^**^	21.38^**^	206.18^**^	230.20^**^	17.18^**^	19.19^**^	5.55^**^	6.20^**^
I X SiNPs	4	0.68^**^	0.53^**^	0.96^**^	0.75^**^	10.30^**^	8.11^**^	0.86^**^	0.68^**^	0.28^**^	0.22^**^
Pooled error A	12	0.1182	0.0845	0.167	0.119	1.80	1.28	0.15	0.11	0.05	0.03
Genotypes (G)	7	36.55^**^	41.35^**^	51.54^**^	58.31^**^	554.98^**^	627.94^**^	46.25^**^	52.33^**^	14.94^**^	16.90^**^
I X G	14	2.268^**^	2.351^**^	3.20^**^	3.32^**^	34.45^**^	35.70^**^	2.87^**^	2.98^**^	0.93^**^	0.96^**^
SiNPs X G	14	0.676^**^	0.74^**^	0.95^**^	1.04^**^	10.26^**^	11.25^**^	0.86^**^	0.94^**^	0.28^**^	0.30^**^
I x SiNPs x G	28	0.62^**^	0.77^**^	0.88^**^	1.08^**^	9.48^**^	11.64^**^	0.79^**^	0.97^**^	0.26^**^	0.31^**^
Pooled Error B	126	0.1238	0.0894	0.175	0.126	1.88	1.36	0.16	0.11	0.05	0.04

**Notes.**

*, ** Significant at 0.05 and 0.01 probability level, respectively.

## Irrigation Regime Effects

### Physiological responses

**Figure 2:**
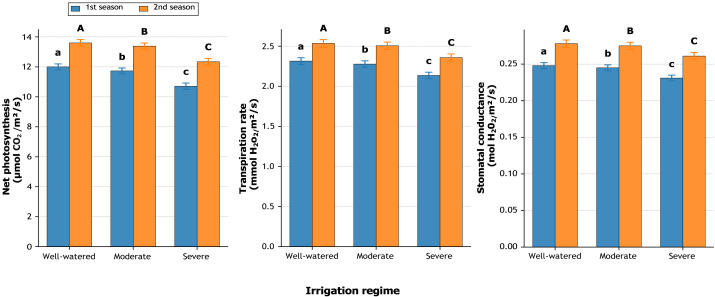
Effect of irrigation regimes on net photosynthesis (µmol CO_2_ m^−2^ s^−1^), transpiration rate (mmol H_2_O m^−2^ s^−1^), and stomatal conductance (mol H_2_O m^−2^ s^−1^) during two growing seasons. Values represent mean ± standard error (SE). Different letters above the bars indicate significant differences among treatments according to Duncan’s multiple range test at *p* ≤ 0.05.

Irrigation regime exerted a highly significant (*p* ≤ 0.01) effect on photosynthetic activity, transpiration rate, and stomatal conductance during both growing seasons (Fig. 2). Well-watered plants recorded the maximum net photosynthetic rate, reaching 12.00 and 13.60 µmol CO_2_ m^−2^ s^−1^ in the first and second seasons, respectively. Well-watered plants recorded the highest photosynthetic rate, reaching 12.00 and 13.60 µmol CO_2_ m^−2^ s^−1^ in the first and second seasons, respectively. These values were slightly higher than those under moderate irrigation (11.7 and 13.4 µmol CO_2_ m^−2^ s^−1^) and clearly greater than under severe water deficit (10.6 and 12.2 µmol CO_2_ m^−2^ s^−1^). This indicates that sufficient water supply directly enhanced carbon assimilation capacity.

Similarly (Fig. 2), transpiration rate was highest under well-watered conditions (2.314 and 2.535 mmol H_2_O m^−2^ s^−1^), followed by moderate (2.277 and 2.505) and severe (2.135 and 2.357) regimes. The decline under severe stress compared to well-watered treatment amounted to −7.7% in season one and −7.0% in second season. Stomatal conductance showed parallel trends, dropping from 0.248–0.278 mol m^−2^ s^−1^ under well-watered to 0.231–0.261 mol m^−2^ s^−1^ under severe stress. This reduction represented −6.9% in season one and −6.1% in season two, indicating partial stomatal closure as a conservative strategy under limited water supply.

### Water relations

**Figure 3:**
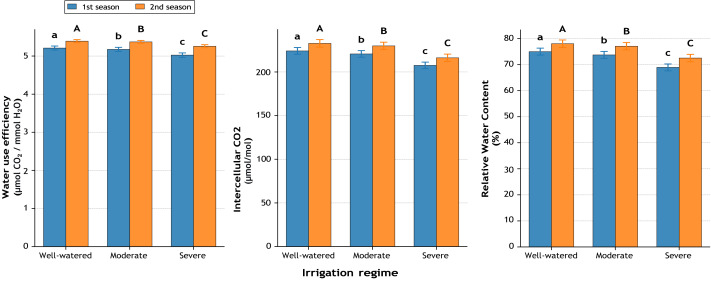
Effect of irrigation regimes on water use efficiency (µmol CO_2_ mmol^−1^ H_2_O), intercellular CO2 concentration (µmol mol^−1^), and relative water content (%) during two growing seasons. Values represent mean ± standard error (SE). Different letters above the bars indicate significant differences among treatments according to Duncan’s multiple range test at *p* ≤ 0.05.

Water use efficiency (WUE) showed minor but significant differences (Fig. 3), with the highest values under well-watered conditions (5.21 and 5.39) compared to severe stress (5.03 and 5.26). This decline reached −3.6% and −2.5%, respectively, highlighting that water deficit restricted photosynthesis more than it reduced transpiration. Intercellular CO_2_ concentration (Ci) decreased progressively with increasing water stress (Fig. 3). Under well-watered conditions, Ci was 224.1 and 232.7 µmol mol^−1^, while under severe stress it dropped to 207.5 and 216.2 µmol mol^−1^, equating to reductions of −7.4% and −7.1%. Relative water content (RWC) followed the same pattern, falling from 75.0% and 78.0% in well-watered plants to 68.9% and 72.5% under severe stress, corresponding to losses of −8.1% and −7.0% (Fig. 3). These values demonstrate that drought stress substantially reduced plant hydration status.

### Photosynthetic pigments

**Figure 4:**
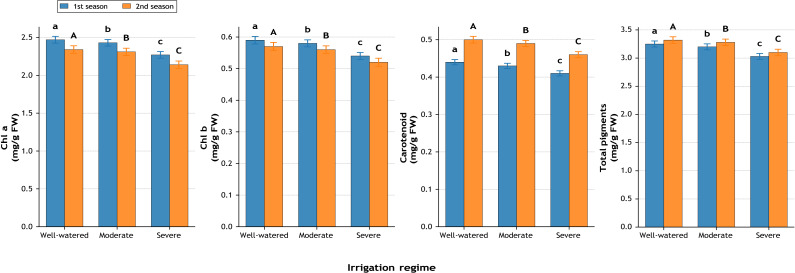
Effect of irrigation regime on chlorophyll a, chlorophyll b, carotenoid, and total photosynthetic pigments (mg g^−1^ FW) during two growing seasons. Values represent mean ± standard error (SE). Different letters above the bars indicate significant differences among treatments according to Duncan’s multiple range test at *p* ≤ 0.05.

Chlorophyll pigments were strongly influenced by water regime (Fig. 4). Chlorophyll a under severe stress was reduced to 2.27 and 2.14 mg g^−1^ FW, compared with 2.47 and 2.34 mg g^−1^ FW in well-watered plants, translating into losses of −8.1% and −8.5%. Chlorophyll b exhibited similar declines (−8.5% and −8.8%). Carotenoids declined by −6.8% in the first season and −8.0% in the second season. Consequently, total pigments decreased from 3.25–3.32 mg g^−1^ FW in well-watered to 3.03–3.10 mg g^−1^ FW under severe stress, equivalent to −6.8% and −6.6%, confirming pigment degradation under water limitation.

### Growth traits

**Figure 5:**
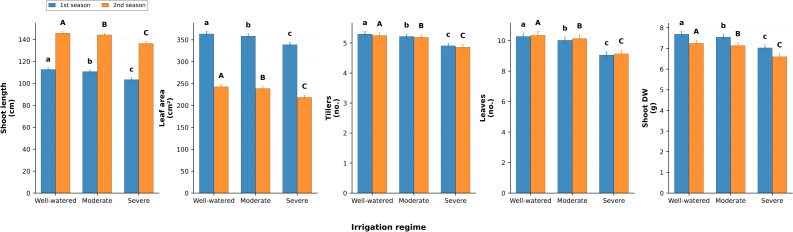
Effect of irrigation regimes on shoot length (cm), leaf area per plant (cm^2^), number of tillers per plant, number of leaves per plant, and shoot dry weight (g) during two growing seasons. Values represent mean ± standard error (SE). Different letters above the bars indicate significant differences among treatments according to Duncan’s multiple range test at *p* ≤ 0.05.

Growth attributes were also significantly reduced by water stress (Fig. 5). Shoot length declined from 112.8 and 145.8 cm in well-watered plants to 103.5 and 136.5 cm under severe stress, reductions of −8.2% and −6.4%. Leaf area per plant fell from 363.6 and 243.0 cm^2^ to 339.2 and 218.6 cm^2^, corresponding to −6.7% and −10.1%. Tillering capacity decreased from 5.3 and 5.25 tillers per plant under well-watered conditions to 4.91 and 4.86 under severe stress, with respective declines of −7.4% and −7.4%. Number of leaves also decreased (−11.9% and −11.7%) under stress. Shoot dry weight per plant was most affected, dropping by −8.6% and −9.0%, confirming reduced biomass accumulation under limited irrigation.

### Yield components

**Figure 6:**
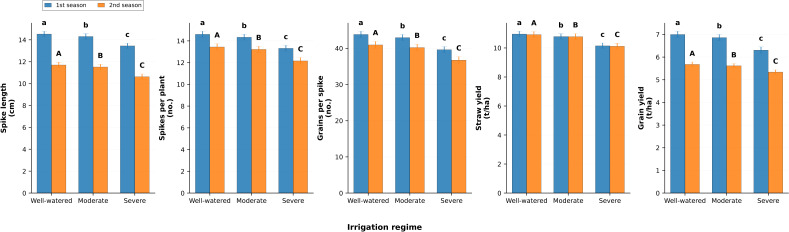
Effect of irrigation regimes on spike length (cm), number of spikes per plant, number of grains per spike, straw yield (t/ha), and grain yield (t/ha) during two growing seasons. Values represent mean ± standard error (SE). Different letters above the bars indicate significant differences among treatments according to Duncan’s multiple range test at *p* ≤ 0.05.

Yield traits demonstrated the cumulative effect of water regime (Fig. 6). Spike length was reduced by −7.4% in season one and −9.1% in season two under severe stress compared with well-watered conditions. The number of spikes per plant decreased by −8.8% and −9.5%, while grains per spike declined by −9.7% and −9.5%. Straw yield per hectare declined from 10.96 and 10.92 t ha^−1^ under well-watered to 10.15 and 10.12 t ha^−1^ under severe stress, representing losses of −7.4% and −7.3%. Grain yield was also significantly reduced, falling from 7.0 and 5.68 t ha^−1^ under well-watered to 6.31 and 5.34 t ha^−1^ under severe irrigation, representing decreases of −9.9% and −6.0%, respectively.

### Overall trend

The findings clearly indicate that severe irrigation deficit significantly suppressed physiological efficiency, pigment stability, growth, and yield, with reductions ranging from 6–12% across traits. Moderate irrigation exerted milder effects, generally reducing performance by only 1–3% compared with well-watered conditions. Thus, well-watered plants consistently achieved the highest photosynthetic performance, water balance, biomass accumulation, and final yield, while severe water stress substantially limited productivity.

#### Application of silicon nanoparticles (SiNPs) effects

**Figure 7:**
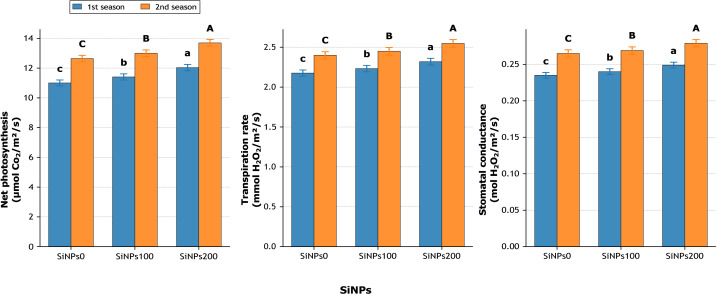
Effect of SiNPs application on net photosynthesis (µmol CO_2_ m^−2^ s^−1^), transpiration rate (mmol H_2_O m^−2^ s^−1^), and stomatal conductance (mol H_2_ O m^−2^ s^−1^) during two growing seasons. Values represent mean ± standard error (SE). Different letters above the bars indicate significant differences among treatments according to Duncan’s multiple range test at *p* ≤ 0.05.

**Figure 8:**
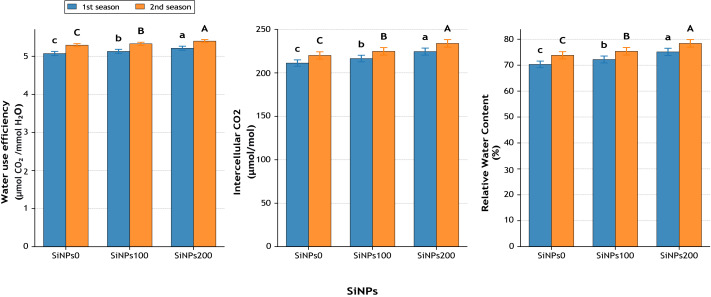
Effect of SiNPs application on water use efficiency (µmol CO_2_ mmol^−1^ H_2_O), intercellular CO_2_ concentration (µmol mol^−1^), and relative water content (%) during two growing seasons. Values represent mean ± standard error (SE). Different letters above the bars indicate significant differences among treatments according to Duncan’s multiple range test at *p* ≤ 0.05.

**Figure 9:**
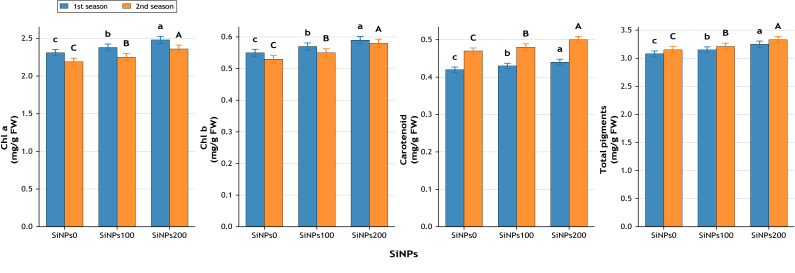
Effect of SiNPs application on chlorophyll a (mg g^−1^ FW), chlorophyll b (mg g^−1^ FW), carotenoids (mg g^−1^ FW), and total photosynthetic pigments (mg g^−1^ FW) during two growing seasons. Values represent mean ± standard error (SE). Different letters above the bars indicate significant differences among treatments according to Duncan’s multiple range test at *p* ≤ 0.05.

**Figure 10:**
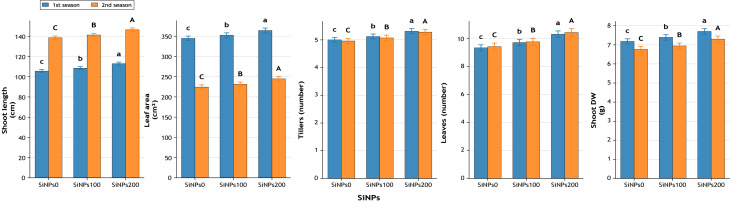
Effect of silicon nanoparticles (SiNPs) on shoot length (cm), leaf area per plant (cm^2^), number of tillers per plant, number of leaves per plant, and shoot dry weight (g plant^−1^) of Wheat during two seasons. Values represent mean ± standard error (SE). Different letters above the bars indicate significant differences among treatments according to Duncan’s multiple range test at *p* ≤ 0.05.

**Figure 11:**
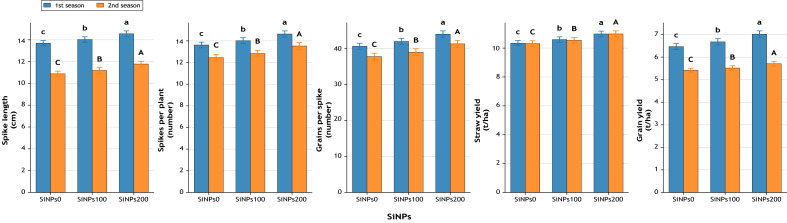
Effect of SiNPs application on spike length (cm), number of spikes per plant, number of grains per spike, straw yield (t ha^−1^), and grain yield (t ha^−1^) during two growing seasons. Values represent mean ± standard error (SE). Different letters above the bars indicate significant differences among treatments according to Duncan’s multiple range test at *p* ≤ 0.05.

The application of silicon nanoparticles (SiNPs) exerted highly significant effects (*p* ≤ 0.01) on all measured physiological, biochemical, growth, and yield traits across both seasons (Figs. 7–11). A consistent dose-dependent improvement was observed, with the highest rate (200 mg L^−1^) outperforming both the untreated control and the moderate application (100 mg L^−1^).

#### Physiological traits

Net photosynthesis (Fig. 7) increased significantly with SiNPs application. Compared with the untreated control, SiNPs200 enhanced photosynthetic rate by 9.4% in the first season (12.03 *vs.* 11.00 µmol CO_2_ m^−2^ s^−1^) and 8.3% in the second season (13.69 *vs.* 12.64 µmol CO_2_ m^−2^ s^−1^). Transpiration rate (E) followed a similar trend, increasing by 6.6% and 6.2% across seasons, while stomatal conductance (gs) rose by 6.0% and 5.3%, respectively, under SiNPs200 relative to the control (Fig. 7). Water use efficiency (WUE) was also improved, rising by 2.7% in the first season and 2.0% in the second season (Fig. 8), indicating a more efficient balance between carbon assimilation and water loss. Importantly, relative water content (RWC) (Fig. 8) increased from 70.3% in untreated plants to 75.1% under SiNPs200 in the first season (+6.9%) and from 73.8% to 78.4% in the second season (+6.2%), confirming the role of SiNPs in maintaining plant hydration.

#### Biochemical attributes

Chlorophyll a content increased by 7.4% (2.48 *vs.* 2.31 mg g^−1^ FW) in the first season and 7.8% in the second season (Fig. 9), while chlorophyll b and carotenoids exhibited consistent gains of ∼7–9% with SiNPs200 relative to the control (Fig. 9). Consequently, total photosynthetic pigments (Fig. 9) rose from 3.08 to 3.25 mg g^−1^ FW in the first season (+5.5%) and from 3.15 to 3.33 mg g^−1^ FW in the second season (+5.7%), indicating an enhancement in pigment stability under supplementation.

#### Growth performance

Significant improvements were also recorded in vegetative growth (Fig. 10). Shoot length increased by 7.0% (113.0 *vs.* 105.6 cm) in the first season and 5.7% (146.5 *vs.* 138.7 cm) in the second season under SiNPs200. Leaf area expanded by 5.6% and 9.1%, while the number of tillers and leaves per plant increased by 6.2% and 10.7%, respectively, compared with untreated plants. Shoot dry weight improved by 7.2% in the first season and 8.0% in the second season, suggesting a stronger biomass accumulation with supplementation.

#### Yield attributes

The positive effects of SiNPs extended to yield components (Fig. 11). Spike length increased from 13.7 cm (control) to 14.6 cm under SiNPs200 (+6.3%), while the number of spikes per plant improved by 8.2% in the first season and 8.6% in the second. Similarly, the number of grains per spike rose by 8.3% and 9.3% across seasons. Straw yield showed gains of 6.2% in the first season (10.98 *vs.* 10.34 t ha^−1^) and 6.5% in the second season, while grain yield, the most critical trait, increased by 8.5% (7.02 *vs.* 6.47 t ha^−1^) in the first season and 8.4% (5.71 *vs.* 5.42 t ha^−1^) in the second season under SiNPs200 relative to the control.

#### Overall interpretation

These findings clearly demonstrate that SiNPs supplementation, particularly at 200 mg L^−1^, significantly improved physiological efficiency, pigment composition, water status, and growth attributes, which translated into substantial increases in both straw and grain yields. The improvements ranged between 5–10% for most traits, with grain yield enhancement being particularly noteworthy. This indicates that SiNPs act as an effective strategy to mitigate stress impacts and enhance wheat productivity under field conditions.

### Genotypic variation

**Figure 12:**
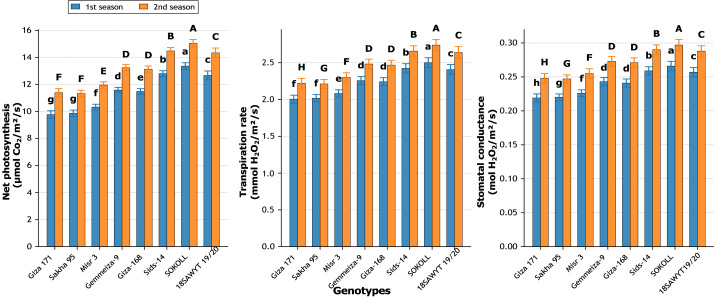
Variation among wheat genotypes in net photosynthesis (µmol CO_2_ m^−2^ s^−1^), transpiration rate (mmol H_2_O m^−2^ s^−1^), and stomatal conductance (mol H_2_O m^−2^ s^−1^) during two growing seasons. Values represent mean ± standard error (SE). Different letters above the bars indicate significant differences among treatments according to Duncan’s multiple range test at *p* ≤ 0.05.

**Figure 13:**
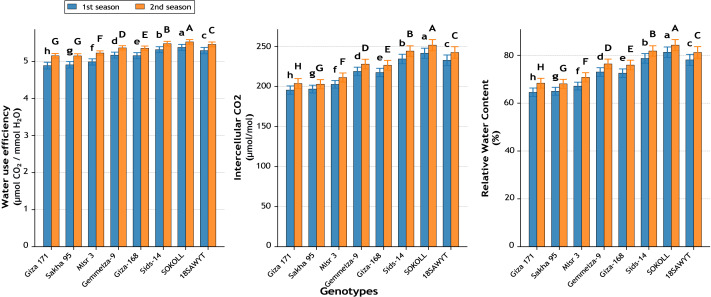
Variation among wheat genotypes in water use efficiency (µmol CO_2_ mmol^−1^ H_2_O), intercellular CO_2_ concentration (µmol mol^−1^), and relative water content (%) during two growing seasons. Values represent mean ± standard error (SE). Different letters above the bars indicate significant differences among treatments according to Duncan’s multiple range test at *p* ≤ 0.05.

**Figure 14:**
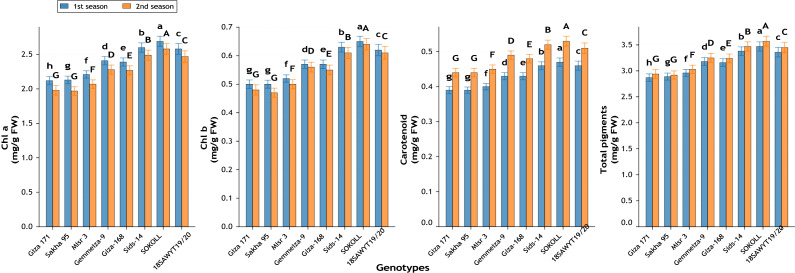
Variation among wheat genotypes in chlorophyll a (mg g^−1^ FW), chlorophyll b (mg g^−1^ FW), carotenoids (mg g^−1^ FW), and total photosynthetic pigments (mg g^−1^ FW) during two growing seasons. Values represent mean ± standard error (SE). Different letters above the bars indicate significant differences among treatments according to Duncan’s multiple range test at *p* ≤ 0.05.

**Figure 15:**
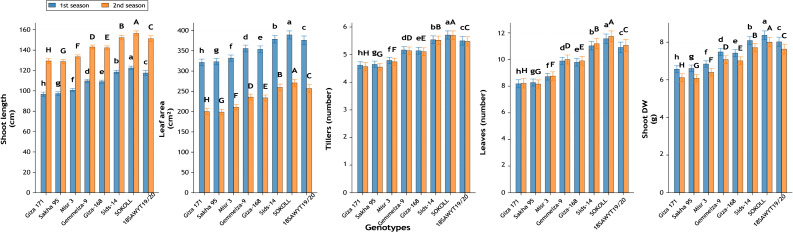
Growth-related traits of wheat genotypes: shoot length (cm), leaf area per plant (cm^2^), number of tillers per plant, number of leaves per plant, and shoot dry weight per plant (g) across two growing seasons. Values represent mean ± standard error (SE). Different letters above the bars indicate significant differences among treatments according to Duncan’s multiple range test at *p* ≤ 0.05.

**Figure 16:**
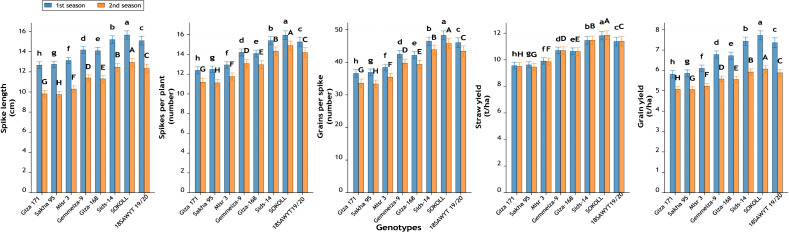
Variation among wheat genotypes in spike length (cm), number of spikes per plant, number of grains per spike, straw yield (t ha^−1^), and grain yield (t ha^−1^) during two growing seasons. Values represent mean ± standard error (SE). Different letters above the bars indicate significant differences among treatments according to Duncan’s multiple range test at *p* ≤ 0.05.

Significant differences (Figs. 12–16) were observed among the eight wheat genotypes for all studied traits across both growing seasons (*p* ≤ 0.01).

#### Physiological traits

SOKOLL consistently recorded the highest values for photosynthesis, transpiration, and stomatal conductance (Fig. 12). In the first season, its photosynthetic rate (13.36 µmol CO_2_ m^−2^ s^−1^) exceeded Giza 171 by 36.8%, while in the second season (15.04 µmol CO_2_ m^−2^ s^−1^), the advantage was 32.1%. Transpiration rate also rose by 24.8% in season one and 23.4% in season two compared with the lowest genotype. Stomatal conductance in SOKOLL reached 0.266 and 0.297 mol m^−2^ s^−1^, representing increases of 21.5% and 19.8% over Giza 171, respectively.

#### Water relations

Water use efficiency (WUE) ranged from 4.89 in Giza 171 to 5.38 in SOKOLL during the first season, a 10.0% gain (Fig. 13). In the second season, WUE improved similarly (+7.3%). Intercellular CO_2_ concentration peaked in SOKOLL (241.4 and 251.6 µmol mol^−1^), surpassing Giza 171 by 23.5% and 23.5%, respectively (Fig. 13). Relative water content (RWC) followed the same trend, with SOKOLL maintaining 81.3% and 84.3%, nearly 26% higher than Giza 171 across both seasons (Fig. 13).

#### Pigment composition

Clear genotypic variation was evident in photosynthetic pigments (Fig. 14). Chlorophyll a increased by 26.9% in season one and 30.3% in season two in SOKOLL relative to Giza 171. Chlorophyll b, carotenoids, and total pigments showed parallel increases, with total pigments reaching 3.47 and 3.57 mg g^−1^ FW in SOKOLL, compared with only 2.87 and 2.94 in Giza 171 (+20.9% and +21.4%).

#### Growth performance

Shoot length was highest in SOKOLL (122.5 and 156.5 cm), compared to Giza 171 (96.7 and 129.4 cm), indicating increments of 26.7% and 21.0%, respectively (Fig. 15). Leaf area expanded from 321.4 to 389.1 cm^2^ per plant (+21.1%) (Fig. 15). Tillering capacity increased by 23.6% in season one and 24.7% in second season (Fig. 15). Shoot dry weight improved by 27.6% in the first season and 31.0% in the second season, confirming superior biomass allocation (Fig. 15).

#### Yield attributes

Genotypic responses were also evident in reproductive traits (Fig. 16). Spike length increased from 12.7 cm in Giza 171 to 15.7 cm in SOKOLL (+23.7%). The number of grains per spike rose from 36.58 to 48.27 (+32.0%). Straw yield per hectare improved from 9.56 and 9.50 t ha^−1^ in Giza 171 to 11.81 and 11.85 t ha^−1^ in SOKOLL, representing increases of +23.5% and +24.7%, respectively. Similarly, grain yield per hectare increased from 5.81 and 5.07 t ha^−1^ in Giza 171 to 7.72 and 6.07 t ha^−1^ in SOKOLL. Grain yield improved by 32.9% in first season and 19.7% in second season, confirming the strong yield advantage of SOKOLL.

#### General pattern

SOKOLL and Sids-14 ranked consistently superior, while Giza 171 and Sakha 95 remained lowest across most traits. The improvements in photosynthetic activity, water retention, pigment concentration, and biomass directly translated into higher straw and grain yields.

### Combined effects of irrigation, silicon, and genotypic variation on physiological, biochemical, growth, and yield traits

The three-way interaction between irrigation regimes, SiNPs, and genotypes (I × SiNPs × G) was statistically significant for almost all measured traits (*p* ≤ 0.01), reflecting that the combined effect of water availability, nano-silicon supplementation, and genetic background shaped the magnitude of physiological, biochemical, growth, and yield responses (Tables S2–S21). This interaction highlights that plant performance under varying water conditions is not determined by a single factor but rather by their collective influence.

In terms of physiological performance, net photosynthesis (A, µmol CO_2_ m^−2^ s^−1^) showed marked variation under the interaction. Well-watered plants averaged 12.00 µmol CO_2_ m^−2^ s^−1^ in the first season compared with only 10.71 µmol CO_2_ m^−2^ s^−1^ under severe stress, representing a 12.1% reduction. However, genotype-specific responses under SiNPs application were evident, as SOKOLL reached 15.04 µmol CO_2_ m^−2^ s^−1^ with 200 mg L^−1^ SiNPs under stress, while Giza 171 recorded values below 11.0 µmol CO_2_ m^−2^ s^−1^ without supplementation. Similar patterns were observed for transpiration rate (E, mmol H_2_O m^−2^ s^−1^) and stomatal conductance (gs, mol H_2_O m^−2^ s^−1^), where well-watered plants maintained 2.53 mmol H_2_O m^−2^ s^−1^ and 0.278 mol H_2_O m^−2^ s^−1^, respectively, while severe stress reduced these to 2.36 mmol H_2_O m^−2^ s^−1^ and 0.261 mol H_2_O m^−2^ s^−1^, equivalent to declines of 8.4% and 6.1%. The significance of the interaction was further evident in water use efficiency (WUE, µmol CO_2_ mmol^−1^ H_2_O), which declined from 5.21 µmol CO_2_ mmol^−1^ H_2_O in well-watered plants to 5.03 µmol CO_2_ mmol^−1^ H_2_O under stress. Intercellular CO_2_ concentration (Ci, µmol mol^−1^) decreased by 8.0%, from 224.1 to 207.5 µmol mol^−1^, and relative water content (RWC, %) fell sharply by 8.8%, from 75.0% to 68.9%. Importantly, the magnitude of these reductions was moderated in tolerant genotypes receiving SiNPs200, confirming that the significance of the three-way interaction arises from genotype-specific buffering under supplementation.

Pigment composition also reflected the interaction. Chlorophyll a (mg g^−1^ FW) decreased from 2.47 mg g^−1^ FW under well-watered conditions to 2.27 mg g^−1^ FW under severe stress, a reduction of 8.8%, while chlorophyll b decreased from 0.59 to 0.54 mg g^−1^ FW (−9.3%). Carotenoids fell from 0.44 to 0.41 mg g^−1^ FW (−7.3%), and total pigments declined from 3.25 to 3.03 mg g^−1^ FW (−7.3%). Yet, SOKOLL and Sids-14 maintained significantly higher pigment concentrations under drought when supplied with 200 mg L^−1^ SiNPs, in contrast to Giza 171 and Sakha 95 which exhibited the lowest values in the absence of supplementation. This genotypic disparity in pigment stability under varying irrigation regimes explains the strong significance of the three-way interaction term.

Growth parameters were similarly influenced. Shoot length (cm) decreased by 9.0% between well-watered (112.8 cm) and severely stressed plants (103.5 cm), while leaf area per plant (cm^2^) was reduced by 7.2% (363.6 *vs.* 339.2 cm^2^). The number of tillers per plant fell from 5.30 to 4.91 (−7.9%), and the number of leaves per plant from 10.28 to 9.06 (−13.5%). Shoot dry weight (g plant^−1^) also declined by 9.4%, from 7.69 g plant^−1^ under well-watered conditions to 7.03 g plant^−1^ under severe stress. Within these averages, the effect of SiNPs200 was clearly genotype-dependent, with SOKOLL producing shoots longer than 156 cm and dry matter above 8.0 g plant^−1^ under stress, whereas Giza 171 remained below 130 cm and 6.1 g plant^−1^, thus reflecting the significance of the interaction.

Yield components and final yields were also significantly shaped by the three-way interaction. Spike length (cm) was reduced by 6.1%, from 14.53 cm in well-watered plants to 13.70 cm in severely stressed plants. The number of spikes per plant declined from 14.61 to 13.62 (−7.3%), and the number of grains per spike dropped by 8.0%, from 43.9 to 40.6 grains. Grain yield per hectare (t ha^−1^) exhibited one of the most striking differences: while the average fell from 5.68 t ha^−1^ under well-watered conditions to 5.34 t ha^−1^ under severe deficit (−6%), genotype × SiNPs contrasts revealed much larger divergences. In this regard, SOKOLL supplemented with 200 mg L^−1^ SiNPs under stress produced 6.54 t ha^−1^, which was 9% higher than Giza 171 at 5.92 t ha^−1^ in the corresponding treatment combination. Straw yield (t ha^−1^) showed a similar pattern, decreasing from 10.92 t ha^−1^ under well-watered conditions to 10.12 t ha^−1^ under severe stress (−7%), but the extent of reduction varied markedly among genotypes and SiNP treatments.

Taken together, the significant three-way interaction across all groups of traits demonstrates that irrigation regime, SiNPs application, and genotype jointly determine the extent of physiological efficiency, pigment stability, growth vigor, and yield performance. The results clearly indicate that drought effects ranged from 5% to 13% reductions across traits, but these losses were strongly mitigated in tolerant genotypes when supplemented with 200 mg L^−1^ SiNPs, as evidenced by improvements exceeding 40% in grain yield relative to susceptible, untreated cultivars.

## Discussion

The application of SiNPs significantly improved the physiological performance of wheat under both moderate and severe water stress (*p* ≤ 0.01). Under moderate stress, net photosynthesis (A, µmol CO_2_ m^−2^ s^−1^) increased by 5.71% in Misr 3 and 4.94% in Gemmeiza-9 at 100 mg L^−1^, while 200 mg L^−1^ induced greater benefits, with increases of 13.51% in Gemmeiza-9 and 13.14% in Giza 171 (Abdo et al., 2024; Elsayed et al., 2024). Under severe stress, Giza 171 achieved a 57.19% increase in photosynthesis at 200 mg L^−1^, with parallel enhancements in transpiration (E, mmol H_2_O m^−2^ s^−1^) and stomatal conductance (gs, mol H_2_O m^−2^ s^−1^) (Christian et al., 2023; Abdo et al., 2024). These responses were accompanied by higher water use efficiency (WUE, µmol CO_2_ mmol^−1^ H_2_O) and relative water content (RWC, %), indicating improved water balance (Raza et al., 2023; Ning et al., 2023). Physiologically, these effects can be attributed to the role of silicon in improving stomatal regulation, enhancing leaf hydration, and maintaining mesophyll activity, which collectively sustain CO_2_ assimilation and delay drought-induced metabolic limitations (Raza et al., 2023; Fedorov & Pilkington, 2023; Ahmad et al., 2024).

Biochemical traits also showed clear improvements with SiNPs. Under moderate stress, Misr 3 and Gemmeiza-9 at 100 mg L^−1^ recorded increases in chlorophyll a of 4.67% and 4.25% and in chlorophyll b of 5.18% and 4.66%, respectively (Abdo et al., 2024). At 200 mg L^−1^, Gemmeiza-9 showed stronger responses, with chlorophyll a rising by 11.49% and chlorophyll b by 12.60% (Raza et al., 2023; Abdo et al., 2024). Under severe stress, 100 mg L^−1^ SiNPs increased chlorophyll a by 3.83% in Giza 168 and 3.44% in SOKOLL (Elsayed et al., 2024), while at 200 mg L^−1^, Giza 171 and SOKOLL exhibited much higher gains of 45.50% and 20.80%, respectively (Davoudi et al., 2024). The enhancement of chlorophyll fractions, carotenoids, and total pigments highlights the ability of SiNPs to protect chloroplast membranes, reduce pigment degradation under oxidative stress, and enhance antioxidant activity, thereby maintaining photosynthetic efficiency (Elsayed et al., 2024; Seyedsharifi, Gasemi Kalkhoran & Narimani, 2024; Fedorov & Pilkington, 2023).

Growth parameters were also enhanced. At 100 mg L^−1^ under moderate stress, Gemmeiza-9 and Giza 171 recorded significant increases in shoot length, leaf area, number of leaves, and dry weight (Raza et al., 2023; Abdo et al., 2024; Elsayed et al., 2024). At 200 mg L^−1^, Gemmeiza-9 showed 10.23% longer shoots and 13.13% larger leaf area (Raza et al., 2023; Elsayed et al., 2024). Under severe stress, Giza 168 and SOKOLL responded strongly to 100 mg L^−1^, while Giza 171 treated with 200 mg L^−1^ exhibited a 69.08% increase in leaf number (Tina et al., 2023; Elsayed et al., 2024). These growth gains reflect physiological improvements, where silicon enhanced turgor maintenance, delayed senescence, and promoted assimilate partitioning. Regulation of hormonal and sugar metabolism further contributed to sustained growth (El-Mantawy, Mokhtar & El-Sherpiny, 2022; Tina et al., 2023; Ahmad et al., 2024).

Yield traits confirmed the cumulative benefits of SiNPs. Under moderate stress, Misr 3 and Gemmeiza-9 showed yield gains of 5.59% and 4.98% at 100 mg L^−1^ (Raza et al., 2023; Abdo et al., 2024). At 200 mg L^−1^, Gemmeiza-9 achieved 13.47% more grains per spike (Raza et al., 2023). Interestingly, the relatively lower grain yield losses observed under severe water stress, despite the 1/3 irrigation level, can be explained by enhanced water-use efficiency (WUE) and relative water content (RWC) due to SiNPs application. These improvements maintained photosynthetic activity and grain filling, while a higher straw yield indicated a shift toward vegetative biomass allocation under stress conditions. Under severe stress, Giza 168 and SOKOLL at 100 mg L^−1^ showed increases across spike length, spike number, grain number, and yield, while Giza 171 at 200 mg L^−1^ achieved 56.52% more grains per spike and 50.33% higher grain yield (Raza et al., 2023; Christian et al., 2023; Abdo et al., 2024). These higher grain and straw yields under severe drought are consistent with findings from other studies, where silicon nanoparticles or biochar + silicon enhanced wheat growth, biomass, and yield under water deficit conditions (Johnson et al., 2022; Raza et al., 2023; Al-Tabbal, Al-Harahsheh & Al-Zou’by, 2024; Zulfiqar et al., 2024; Keke et al., 2025). Physiologically, these improvements stem from sustained photosynthesis, stronger source–sink relations, and greater carbon assimilation efficiency, ensuring better spike fertility and grain filling (Tina et al., 2023; Davoudi et al., 2024; Elsayed et al., 2024).

Irrigation itself had strong effects, reducing photosynthesis by 9.2% (13.60 to 12.34 µmol CO_2_ m^−2^ s^−1^), stomatal conductance by 6.1% (0.278 to 0.261 mol H_2_O m^−2^ s^−1^), transpiration by 6.7% (2.53 to 2.36 mmol H_2_O m^−2^ s^−1^), and RWC by 7.0% (78.0% to 72.5%). Chlorophyll a declined by 8.5% (2.34 to 2.14 mg g^−1^ FW), shoot length by 6.4% (145.8 to 136.5 cm), and grain yield by 6.1% (5.68 to 5.34 t ha^−1^). Yet SiNPs at 200 mg L^−1^ significantly alleviated these reductions, increasing photosynthesis by 8.3%, WUE to 5.40 µmol CO_2_ mmol^−1^ H_2_O, RWC to 78.4%, chlorophyll a by 7.7%, shoot length by 5.6%, and grain yield by 5.4% (Abdo et al., 2024; Elsayed et al., 2024).

Genotypic differences were decisive. SOKOLL consistently recorded the highest values across traits, including 15.04 µmol CO_2_ m^−2^ s^−1^ in photosynthesis, 2.74 mmol H_2_O m^−2^ s^−1^ in transpiration, 0.297 mol H_2_O m^−2^ s^−1^ in stomatal conductance, 5.53 µmol CO_2_ mmol^−1^ H_2_O in WUE, 251.6 µmol mol^−1^ in Ci, and 84.3% in RWC. It also had the highest pigment content, with chlorophyll a at 2.58 mg g^−1^ FW and total pigments at 3.57 mg g^−1^ FW, alongside greater shoot length (156 cm), leaf area (>270 cm^2^), and dry weight (8.0 g plant^−1^). These vegetative advantages translated into yield superiority, with spike length of 12.94 cm, 14.9 grains per spike, and grain yield of 6.07 t ha^−1^, outperforming Giza 171 and Sakha 95. These genotypic differences reflect variation in root uptake efficiency, silicon accumulation, and stress-responsive mechanisms.

Taken together, the significant three-way interaction (I × SiNPs × G) across all traits demonstrates that wheat performance under drought is governed by the combined influence of water supply, silicon supplementation, and genotype. Drought reduced most traits by 5–13%, but tolerant genotypes supplemented with 200 mg L^−1^ SiNPs achieved yield gains exceeding 40% relative to susceptible cultivars. Importantly, the novelty of this study lies in its execution under the harsh climatic conditions of Al-Dawadmi, Saudi Arabia, a region characterized by high temperature, low rainfall, and sandy soils. Conducting this work in such an environment provides new insights into the capacity of SiNPs to mitigate drought stress in arid and semi-arid regions, demonstrating the practical value of integrating nanotechnology with genotype selection to sustain wheat productivity under climate change challenges. Nevertheless, while SiNPs application exhibited promising outcomes under controlled field conditions, its large-scale practical adoption should also consider cost-effectiveness, environmental safety, and compliance with agricultural nanomaterial regulations. Evaluating production cost, field stability, and regulatory approval will be essential steps before recommending SiNPs as a sustainable agronomic practice in commercial wheat farming (Khot et al., 2012; Parisi, Vigani & Rodríguez-Cerezo, 2015).

## Conclusion

The findings of this study support our hypothesis that foliar application of silicon nanoparticles (SiNPs) mitigated some of the negative effects of water stress on the physiological and agronomic performance of wheat genotypes. Compared to the untreated control, SiNPs applied at 200 mg L^−1^ improved physiological and agronomic traits under different water stress regimes, particularly under severe stress. Among the tested genotypes, Giza 171, SOKOLL, and Giza 168 exhibited the most pronounced improvements, highlighting genotype-specific responses. These results demonstrate the potential of SiNP application to enhance drought tolerance in wheat under the specific agro-ecological conditions of Al-Dawadmi. While confirming previous findings on the role of silicon in mitigating drought stress, this study provides novel insights into the differential responses of wheat genotypes in an arid environment.

For future research, we recommend conducting multi-location and long-term trials to validate the effectiveness of SiNP applications under diverse agro-ecological conditions. In addition, studies should evaluate the ecological and soil impacts of SiNPs, including potential accumulation and biosafety concerns. Integration of SiNP application with breeding programs for drought-tolerant wheat genotypes could enhance practical utility. Finally, cost–benefit analyses are necessary to assess the feasibility and economic sustainability of adopting SiNP foliar applications in wheat production systems.

##  Supplemental Information

10.7717/peerj.20823/supp-1 Supplemental Information 1 Raw data

10.7717/peerj.20823/supp-2 Supplemental Information 2 Net photosynthesis of eight wheat genotypes as affected by foliar application of silicon nanoparticles (SiNPs) under well-watered, moderate and severe water stress conditions during winter seasons of 2022/2023 (1 st ) and 2023/2024 (2 nd ) The data of three replicates ± SE (standard error) are shown. Means followed by different letters under the same water regimes were significantly different according to Duncan’s Multiple Range Test (*p* ≤ 0.05)

10.7717/peerj.20823/supp-3 Supplemental Information 3 Transpiration rate of eight wheat genotypes as affected by foliar application of silicon nanoparticles under well-watered, moderate and severe water stress conditions during winter seasons of 2022/2023 (1 st ) and 2023/2024 (2 nd ) The data of three replicates ± SE (standard error) are shown. Means followed by different letters under the same water regimes were significantly different according to Duncan’s Multiple Range Test (*p* ≤ 0.05)

10.7717/peerj.20823/supp-4 Supplemental Information 4 Stomatal conductance of eight wheat genotypes as affected by foliar application of silicon nanoparticles under well-watered, moderate and severe water stress conditions during winter seasons of 2022/2023 (1st) and 2023/2024 (2nd) The data of three replicates ± SE (standard error) are shown. Means followed by different letters under the same water regimes were significantly different according to Duncan’s Multiple Range Test (*p* ≤ 0.05)

10.7717/peerj.20823/supp-5 Supplemental Information 5 Water use efficiency of eight wheat genotypes as affected by foliar application of silicon nanoparticles under well-watered, moderate and severe water stress conditions during winter seasons of 2022/2023 (1 st) and 2023/2024 (2nd) The data of three replicates ± SE (standard error) are shown. Means followed by different letters under the same water regimes were significantly different according to Duncan’s Multiple Range Test (*p* ≤ 0.05)

10.7717/peerj.20823/supp-6 Supplemental Information 6 Intercellular CO_2_ of eight wheat genotypes as affected by foliar application of silicon nanoparticles under well-watered, moderate and severe water stress conditions during winter seasons of 2022/2023 (1 st) and 2023/2024 (2nd) The data of three replicates ± SE (standard error) are shown. Means followed by different letters under the same water regimes were significantly different according to Duncan’s Multiple Range Test (*p* ≤ 0.05)

10.7717/peerj.20823/supp-7 Supplemental Information 7 Relative water content of eight wheat genotypes as affected by foliar application of silicon nanoparticles under well-watered, moderate and severe water stress conditions during winter seasons of 2022/2023 (1st) and 2023/2024 (2nd) The data of three replicates ± SE (standard error) are shown. Means followed by different letters under the same water regimes were significantly different according to Duncan’s Multiple Range Test (*p* ≤ 0.05)

10.7717/peerj.20823/supp-8 Supplemental Information 8 Chlorophyll a of eight wheat genotypes as affected by foliar application of silicon nanoparticles under well-watered, moderate and severe water stress conditions during winter seasons of 2022/2023 (1st) and 2023/2024 (2nd) The data of three replicates ± SE (standard error) are shown. Means followed by different letters under the same water regimes were significantly different according to Duncan’s Multiple Range Test (*p* ≤ 0.05)

10.7717/peerj.20823/supp-9 Supplemental Information 9 Chlorophyll b of eight wheat genotypes as affected by foliar application of silicon nanoparticles under well-watered, moderate and severe water stress conditions during winter seasons of 2022/2023 (1st) and 2023/2024 (2nd) The data of three replicates ± SE (standard error) are shown. Means followed by different letters under the same water regimes were significantly different according to Duncan’s Multiple Range Test (*p* ≤ 0.05)

10.7717/peerj.20823/supp-10 Supplemental Information 10 Carotenoid of eight wheat genotypes as affected by foliar application of silicon nanoparticles under well-watered, moderate and severe water stress conditions during winter seasons of 2022/2023 (1st) and 2023/2024 (2nd) The data of three replicates ± SE (standard error) are shown. Means followed by different letters under the same water regimes were significantly different according to Duncan’s Multiple Range Test (*p* ≤ 0.05)

10.7717/peerj.20823/supp-11 Supplemental Information 11 Total photosynthetic pigments of eight wheat genotypes as affected by foliar application of silicon nanoparticles under well-watered, moderate and severe water stress conditions during winter seasons of 2022/2023 (1st) and 2023/2024 (2nd) The data of three replicates ± SE (standard error) are shown. Means followed by different letters under the same water regimes were significantly different according to Duncan’s Multiple Range Test (*p* ≤ 0.05)

10.7717/peerj.20823/supp-12 Supplemental Information 12 Shoot length of eight wheat genotypes as affected by foliar application of silicon nanoparticles under well-watered, moderate and severe water stress conditions during winter seasons of 2022/2023 (1st) and 2023/2024 (2nd) The data of three replicates ± SE (standard error) are shown. Means followed by different letters under the same water regimes were significantly different according to Duncan’s Multiple Range Test (*p* ≤ 0.05)

10.7717/peerj.20823/supp-13 Supplemental Information 13 The data of three replicates ± SE (standard error) are shown. Means followed by different letters under the same water regimes were significantly different according to Duncan’s Multiple Range Test (*p* ≤ 0.05)

10.7717/peerj.20823/supp-14 Supplemental Information 14 No. of tillers per plant of eight wheat genotypes as affected by foliar application of silicon nanoparticles under well-watered, moderate and severe water stress conditions during winter seasons of 2022/2023 (1st) and 2023/2024 (2nd) The data of three replicates ± SE (standard error) are shown. Means followed by different letters under the same water regimes were significantly different according to Duncan’s Multiple Range Test (*p* ≤ 0.05)

10.7717/peerj.20823/supp-15 Supplemental Information 15 No. of leaves per plant of eight wheat genotypes as affected by foliar application of silicon nanoparticles under well-watered, moderate and severe water stress conditions during winter seasons of 2022/2023 (1st) and 2023/2024 (2nd) The data of three replicates ± SE (standard error) are shown. Means followed by different letters under the same water regimes were significantly different according to Duncan’s Multiple Range Test (*p* ≤ 0.05)

10.7717/peerj.20823/supp-16 Supplemental Information 16 Shoot DW per plant of eight wheat genotypes as affected by foliar application of silicon nanoparticles under well-watered, moderate and severe water stress conditions during winter seasons of 2022/2023 (1st) and 2023/2024 (2nd) The data of three replicates ± SE (standard error) are shown. Means followed by different letters under the same water regimes were significantly different according to Duncan’s Multiple Range Test (*p* ≤ 0.05)

10.7717/peerj.20823/supp-17 Supplemental Information 17 Spike length of eight wheat genotypes as affected by foliar application of silicon nanoparticles under well-watered, moderate and severe water stress conditions during winter seasons of 2022/2023 (1st) and 2023/2024 (2nd) The data of three replicates ± SE (standard error) are shown. Means followed by different letters under the same water regimes were significantly different according to Duncan’s Multiple Range Test (*p* ≤ 0.05)

10.7717/peerj.20823/supp-18 Supplemental Information 18 No. of spikes per plant of eight wheat genotypes as affected by foliar application of silicon nanoparticles under well-watered, moderate and severe water stress conditions during winter seasons of 2022/2023 (1st) and 2023/2024 (2nd) The data of three replicates ± SE (standard error) are shown. Means followed by different letters under the same water regimes were significantly different according to Duncan’s Multiple Range Test (*p* ≤ 0.05)

10.7717/peerj.20823/supp-19 Supplemental Information 19 No. of grains per spike of eight wheat genotypes as affected by foliar application of silicon nanoparticles under well-watered, moderate and severe water stress conditions during winter seasons of 2022/2023 (1st) and 2023/2024 (2nd) The data of three replicates ± SE (standard error) are shown. Means followed by different letters under the same water regimes were significantly different according to Duncan’s Multiple Range Test (*p* ≤ 0.05)

10.7717/peerj.20823/supp-20 Supplemental Information 20 Straw yield per hectare of eight wheat genotypes as affected by foliar application of silicon nanoparticles under well-watered, moderate and severe water stress conditions during winter seasons of 2022/2023 (1st) and 2023/2024 (2nd) The data of three replicates ± SE (standard error) are shown. Means followed by different letters under the same water regimes were significantly different according to Duncan’s Multiple Range Test (*p* ≤ 0.05)

10.7717/peerj.20823/supp-21 Supplemental Information 21 Grain yield per hectare of eight wheat genotypes as affected by foliar application of silicon nanoparticles under well-watered, moderate and severe water stress conditions during winter seasons of 2022/2023 (1st) and 2023/2024 (2nd) The data of three replicates ± SE (standard error) are shown. Means followed by different letters under the same water regimes were significantly different according to Duncan’s Multiple Range Test (*p* ≤ 0.05)

## Additional Information and Declarations

### Data Availability

The following information was supplied regarding data availability: The raw data is available in the Supplemental Files.
