# Supplementary material for: Improving drought tolerance in some wheat genotypes with foliar application of silicon nanoparticles in Al-Dawadmi, Saudi Arabia
Source: PeerJ. 2026 Feb 24;14:e20823. doi: 10.7717/peerj.20823 (PMC12947762; doi:10.7717/peerj.20823)
Supplement: Supplemental Information 2 — The data of three replicates ± SE (standard error) are shown. Means followed by different letters under the same water regimes were significantly different according to Duncan’s Multiple Range Test (p ≤ 0.05) [file peerj-14-20823-s002.docx]

Supplementary Table S1. Net photosynthesis of eight wheat genotypes as affected by foliar application of silicon nanoparticles (SiNPs) under well-watered, moderate and severe water stress conditions during winter seasons of 2022/2023 (1^st^) and 2023/2024 (2^nd^ )

| SiNPs | Net photosynthesis | | | | | | |
| --- | --- | --- | --- | --- | --- | --- | --- |
|  | Genotypes | Well-watered | | Moderate | | Severe | |
|  |  | 1st | 2nd | 1st | 2nd | 1st | 2nd |
| SiNPs_0_ | Giza 171 | 9.49w±0.84 | 11.10v±0.96 | 9.23w±0.84 | 10.84w±0.94 | 8.13v±0.84 | 9.73v±0.86 |
|  | Sakha 95 | 10.27tu±0.88 | 11.89st±1.03 | 9.80uv±0.86 | 11.43uv±0.99 | 8.40tuv±0.83 | 9.99tuv±0.88 |
|  | Misr 3 | 10.41t±0.88 | 12.04s±1.07 | 9.95tu±0.86 | 11.58tu±1.02 | 9.17rs±0.85 | 10.78rs±0.94 |
|  | Gemmeiza-9 | 11.04pqr±0.91 | 12.69pqr±1.13 | 11.51mn±0.95 | 13.15mn±1.18 | 10.90ijk±0.91 | 12.55ijk±1.12 |
|  | Giza-168 | 11.83jkl±0.99 | 13.48jkl±1.22 | 11.28mno±0.94 | 12.93mno±1.16 | 10.20p±0.88 | 11.82p±1.05 |
|  | Sids-14 | 12.76i±1.07 | 14.43i±1.33 | 12.28ijk±1.03 | 13.95jk±1.27 | 11.91d→g±0.98 | 13.58d→g±1.23 |
|  | SOKOLL | 13.22gh±1.12 | 14.89gh±1.38 | 12.69ghi±1.06 | 14.36ghi±1.34 | 12.09def±1.01 | 13.74def±1.25 |
|  | 18 SAWYT 19/20 | 13.69b→f±1.18 | 15.37b→f±1.46 | 13.13def±1.10 | 14.81def±1.37 | 10.64k→o±0.90 | 12.27k→o±1.07 |
| SiNPs_100_ | Giza 171 | 9.83vw±0.84 | 11.45uv±1.01 | 9.45vw±0.83 | 11.07vw±0.98 | 8.46tuv±0.84 | 10.07tuv±0.88 |
|  | Sakha 95 | 10.94p→s±0.91 | 11.16v±0.97 | 10.28q→t±0.87 | 11.92q→t±1.06 | 8.55tu±0.84 | 10.15tu±0.89 |
|  | Misr 3 | 11.32m→p±0.94 | 12.97m→p±1.16 | 10.52pqr±0.89 | 12.15pqr±1.06 | 9.39qr±0.85 | 11.00qr±0.95 |
|  | Gemmeiza-9 | 11.48l→o±0.96 | 13.12l→o±1.16 | 12.08jkl±1.00 | 13.74jkl±1.25 | 11.13hij±0.92 | 12.77hij±1.14 |
|  | Giza-168 | 11.94jk±1.00 | 13.60jk±1.23 | 11.62m±0.96 | 13.28m±1.20 | 10.66k→n±0.89 | 12.30k→n±1.09 |
|  | Sids-14 | 13.43d→g±1.13 | 15.13d→g±1.43 | 12.84fgh±1.08 | 14.51fgh±1.34 | 12.15cde±1.01 | 13.82de±1.26 |
|  | SOKOLL | 13.77a→e±1.17 | 15.47a→e±1.47 | 13.25b→e±1.12 | 14.93cde±1.39 | 12.56bc±1.04 | 14.24bc±1.33 |
|  | 18 SAWYT 19/20 | 14.01ab±1.21 | 15.70abc±1.48 | 13.28bcd±1.11 | 14.96bcd±1.41 | 10.79jkl±0.90 | 12.43jkl±1.11 |
| SiNPs_200_ | Giza 171 | 10.07tuv±0.86 | 11.70stu±1.04 | 10.45qrs±0.88 | 12.08qrs±1.07 | 12.79b±1.06 | 14.47b±1.33 |
|  | Sakha 95 | 11.21n→q±0.93 | 12.85n→q±1.15 | 10.54pq±0.89 | 12.17pq±1.08 | 8.76st±0.83 | 10.37t±0.90 |
|  | Misr 3 | 11.57k→n±0.95 | 13.23k→n±1.19 | 10.90op±0.91 | 12.53p±1.10 | 9.68q±0.85 | 11.30q±0.98 |
|  | Gemmeiza-9 | 11.70klm±0.97 | 13.36klm±1.21 | 13.06d→g±1.10 | 14.74d→g±1.39 | 11.36h±0.94 | 13.00h±1.15 |
|  | Giza-168 | 12.13j±1.02 | 13.78j±1.25 | 12.42ij±1.03 | 14.08ij±1.29 | 11.23hi±0.93 | 12.88hi±1.13 |
|  | Sids-14 | 13.81a→d±1.17 | 15.51a→d±1.48 | 13.75a±1.17 | 15.44a±1.45 | 12.25cd±1.01 | 13.92cd±1.27 |
|  | SOKOLL | 14.01ab±1.21 | 15.71ab±1.50 | 13.60abc±1.16 | 15.27abc±1.43 | 15.02a±1.32 | 16.74a±1.64 |
|  | 18 SAWYT 19/20 | 14.11a±1.21 | 15.81a±1.52 | 13.65ab±1.18 | 15.33ab±1.46 | 10.73j→m±0.91 | 12.36klm±1.08 |
| The data of three replicates ± SE (standard error) are shown.  Means followed by different letters under the same water regimes were significantly different according to Duncan’s Multiple Range Test (p≤ 0.05) | | | | | | | |
