# Supplementary material for: Improving drought tolerance in some wheat genotypes with foliar application of silicon nanoparticles in Al-Dawadmi, Saudi Arabia
Source: PeerJ. 2026 Feb 24;14:e20823. doi: 10.7717/peerj.20823 (PMC12947762; doi:10.7717/peerj.20823)
Supplement: Supplemental Information 3 — The data of three replicates ± SE (standard error) are shown. Means followed by different letters under the same water regimes were significantly different according to Duncan’s Multiple Range Test (p ≤ 0.05) [file peerj-14-20823-s003.docx]

Supplementary Table S2. Transpiration rate of eight wheat genotypes as affected by foliar application of silicon nanoparticles under well-watered, moderate and severe water stress conditions during winter seasons of 2022/2023 (1^st^) and 2023/2024 (2^nd^ )

| SiNPs | Transpiration rate | | | | | | |
| --- | --- | --- | --- | --- | --- | --- | --- |
|  | Genotypes | Well-watered | | Moderate | | Severe | |
|  |  | 1^st^ | 2^nd^ | 1^st^ | 2^nd^ | 1^st^ | 2^nd^ |
| SiNPs_0_ | Giza 171 | 1.966v±0.249 | 2.179v±0.339 | 1.931v±0.242 | 2.142w±0.334 | 1.779t±0.205 | 1.985u±0.310 |
|  | Sakha 95 | 2.075stu±0.279 | 2.292st±0.359 | 2.010s→v±0.260 | 2.226tuv±0.348 | 1.816t±0.215 | 2.022tu±0.315 |
|  | Misr 3 | 2.094rst±0.283 | 2.314s±0.365 | 2.031q→u±0.268 | 2.248stu±0.354 | 1.923qrs±0.240 | 2.135qrs±0.333 |
|  | Gemmeiza-9 | 2.182m→r±0.304 | 2.405n→r±0.380 | 2.247lmn±0.323 | 2.472mn±0.392 | 2.162h→k±0.300 | 2.386h→k±0.378 |
|  | Giza-168 | 2.290jkl±0.334 | 2.518jkl±0.401 | 2.215mno±0.313 | 2.440mno±0.387 | 2.065l→p±0.277 | 2.283m→p±0.360 |
|  | Sids-14 | 2.420ghi±0.367 | 2.652hi±0.424 | 2.353h→k±0.350 | 2.584h→k±0.413 | 2.302c→g±0.334 | 2.532d→g±0.403 |
|  | SOKOLL | 2.483d→h±0.383 | 2.719fgh±0.436 | 2.409d→i±0.365 | 2.643f→i±0.426 | 2.327c→f±0.345 | 2.555c→f±0.407 |
|  | 18 SAWYT 19/20 | 2.548a→f±0.402 | 2.787a→f±0.452 | 2.470a→f±0.378 | 2.707b→f±0.434 | 2.125i→o±0.289 | 2.347j→o±0.368 |
| SiNPs_100_ | Giza 171 | 2.014tuv±0.262 | 2.230tuv±0.351 | 1.961uv±0.248 | 2.176uvw±0.342 | 1.824t±0.213 | 2.033tu±0.317 |
|  | Sakha 95 | 2.168n→s±0.301 | 2.189uv±0.342 | 2.076p→t±0.277 | 2.296q→t±0.362 | 1.837st±0.218 | 2.045tu±0.319 |
|  | Misr 3 | 2.220k→p±0.314 | 2.446l→p±0.388 | 2.110pqr±0.286 | 2.329pqr±0.365 | 1.952qr±0.246 | 2.166qr±0.338 |
|  | Gemmeiza-9 | 2.243j→o±0.322 | 2.467k→o±0.389 | 2.325i→l±0.342 | 2.555jkl±0.407 | 2.194hij±0.309 | 2.417hij±0.382 |
|  | Giza-168 | 2.306jk±0.337 | 2.536jk±0.404 | 2.262klm±0.326 | 2.489lm±0.395 | 2.129i→n±0.290 | 2.351j→n±0.371 |
|  | Sids-14 | 2.512b→g±0.390 | 2.752d→g±0.445 | 2.430d→h±0.369 | 2.664d→h±0.427 | 2.336b→e±0.344 | 2.567cde±0.409 |
|  | SOKOLL | 2.560a→e±0.403 | 2.801a→e±0.454 | 2.488a→e±0.384 | 2.724a→e±0.438 | 2.392bc±0.359 | 2.626bc±0.422 |
|  | 18 SAWYT 19/20 | 2.593ab±0.412 | 2.833abc±0.458 | 2.492a→d±0.386 | 2.729a→d±0.441 | 2.147h→l±0.297 | 2.368i→l±0.374 |
| SiNPs_200_ | Giza 171 | 2.047tuv±0.271 | 2.265stu±0.357 | 2.099p→s±0.284 | 2.320p→s±0.366 | 2.423b±0.365 | 2.658b±0.425 |
|  | Sakha 95 | 2.204l→q±0.311 | 2.429m→q±0.385 | 2.112pq±0.289 | 2.331pq±0.368 | 1.866rst±0.224 | 2.076st±0.323 |
|  | Misr 3 | 2.255j→n±0.322 | 2.483j→n±0.394 | 2.162nop±0.300 | 2.384op±0.374 | 1.992pq±0.254 | 2.209pq±0.344 |
|  | Gemmeiza-9 | 2.273j→m±0.328 | 2.501j→m±0.397 | 2.462a→g±0.379 | 2.698c→g±0.436 | 2.225gh±0.315 | 2.450h±0.385 |
|  | Giza-168 | 2.332ij±0.346 | 2.561j±0.408 | 2.372g→j±0.354 | 2.604hij±0.416 | 2.208hi±0.309 | 2.432hi±0.382 |
|  | Sids-14 | 2.565a→d±0.404 | 2.807a→d±0.455 | 2.556a±0.401 | 2.796a±0.451 | 2.350bcd±0.347 | 2.581bcd±0.411 |
|  | SOKOLL | 2.593ab±0.412 | 2.836ab±0.461 | 2.535abc±0.397 | 2.773abc±0.446 | 2.733a±0.448 | 2.982a±0.488 |
|  | 18 SAWYT 19/20 | 2.607a±0.416 | 2.849a±0.463 | 2.543ab±0.401 | 2.781ab±0.451 | 2.138h→m±0.294 | 2.358i→m±0.370 |
| The data of three replicates ± SE (standard error) are shown.  Means followed by different letters under the same water regimes were significantly different according to Duncan’s Multiple Range Test (p≤ 0.05) | | | | | | | |
