# Supplementary material for: Improving drought tolerance in some wheat genotypes with foliar application of silicon nanoparticles in Al-Dawadmi, Saudi Arabia
Source: PeerJ. 2026 Feb 24;14:e20823. doi: 10.7717/peerj.20823 (PMC12947762; doi:10.7717/peerj.20823)
Supplement: Supplemental Information 4 — The data of three replicates ± SE (standard error) are shown. Means followed by different letters under the same water regimes were significantly different according to Duncan’s Multiple Range Test (p ≤ 0.05) [file peerj-14-20823-s004.docx]

Supplementary Table S3. Stomatal conductance of eight wheat genotypes as affected by foliar application of silicon nanoparticles under well-watered, moderate and severe water stress conditions during winter seasons of 2022/2023 (1^st^) and 2023/2024 (2^nd^ )

| SiNPs | Stomatal conductance | | | | | | |
| --- | --- | --- | --- | --- | --- | --- | --- |
|  | Genotypes | Well-watered | | Moderate | | Severe | |
|  |  | 1^st^ | 2^nd^ | 1^st^ | 2^nd^ | 1^st^ | 2^nd^ |
| SiNPs_0_ | Giza 171 | 0.215v±0.027 | 0.244v±0.037 | 0.212v±0.026 | 0.240w±0.036 | 0.197t±0.023 | 0.225u±0.034 |
|  | Sakha 95 | 0.225stu±0.030 | 0.254st±0.038 | 0.219s→v±0.028 | 0.248tuv±0.037 | 0.200t±0.024 | 0.228tu±0.034 |
|  | Misr 3 | 0.227rst±0.030 | 0.256s±0.039 | 0.221q→u±0.029 | 0.250stu±0.038 | 0.211qrs±0.026 | 0.239qrs±0.036 |
|  | Gemmeiza-9 | 0.236m→r±0.032 | 0.265n→r±0.040 | 0.242lmn±0.034 | 0.272mn±0.041 | 0.234h→k±0.032 | 0.263h→k±0.040 |
|  | Giza-168 | 0.246jkl±0.035 | 0.276jkl±0.042 | 0.239mno±0.033 | 0.269mno±0.041 | 0.224l→p±0.030 | 0.253m→p±0.038 |
|  | Sids-14 | 0.258ghi±0.038 | 0.289hi±0.044 | 0.252h→k±0.037 | 0.282ijk±0.043 | 0.247c→g±0.035 | 0.277d→g±0.042 |
|  | SOKOLL | 0.264d→h±0.040 | 0.295fgh±0.046 | 0.257d→i±0.038 | 0.288f→i±0.045 | 0.249c→f±0.036 | 0.280c→f±0.043 |
|  | 18 SAWYT 19/20 | 0.271a→f±0.042 | 0.302a→f±0.047 | 0.263a→f±0.039 | 0.294b→f±0.045 | 0.230i→o±0.031 | 0.260j→o±0.039 |
| SiNPs_100_ | Giza 171 | 0.219tuv±0.028 | 0.248tuv±0.038 | 0.214uv±0.027 | 0.243uvw±0.037 | 0.201t±0.023 | 0.230tu±0.034 |
|  | Sakha 95 | 0.234n→s±0.032 | 0.244uv±0.037 | 0.225p→t±0.030 | 0.255q→t±0.039 | 0.202st±0.024 | 0.231tu±0.035 |
|  | Misr 3 | 0.239k→p±0.033 | 0.269l→p±0.041 | 0.229pqr±0.031 | 0.258pqr±0.039 | 0.214qr±0.027 | 0.242qr±0.036 |
|  | Gemmeiza-9 | 0.241j→o±0.034 | 0.271k→o±0.041 | 0.249i→l±0.036 | 0.280jkl±0.043 | 0.237hij±0.033 | 0.266hij±0.041 |
|  | Giza-168 | 0.247jk±0.036 | 0.278jk±0.043 | 0.243klm±0.034 | 0.273lm±0.042 | 0.230i→n±0.031 | 0.260j→n±0.039 |
|  | Sids-14 | 0.267a→g±0.041 | 0.298d→g±0.046 | 0.259d→h±0.039 | 0.290d→h±0.045 | 0.250b→e±0.036 | 0.281cde±0.043 |
|  | SOKOLL | 0.272a→e±0.042 | 0.303a→e±0.047 | 0.265a→e±0.040 | 0.296a→e±0.046 | 0.256bc±0.038 | 0.286bc±0.044 |
|  | 18 SAWYT 19/20 | 0.275ab±0.043 | 0.306abc±0.047 | 0.265a→d±0.040 | 0.296a→d±0.046 | 0.232h→l±0.032 | 0.262i→l±0.040 |
| SiNPs_200_ | Giza 171 | 0.223tuv±0.029 | 0.252stu±0.038 | 0.228p→s±0.030 | 0.257p→s±0.039 | 0.259b±0.038 | 0.289b±0.044 |
|  | Sakha 95 | 0.238l→q±0.033 | 0.267m→q±0.041 | 0.229pq±0.031 | 0.258pq±0.039 | 0.205rst±0.025 | 0.234st±0.035 |
|  | Misr 3 | 0.243j→n±0.034 | 0.273j→n±0.042 | 0.234nop±0.032 | 0.263op±0.040 | 0.217pq±0.027 | 0.246pq±0.037 |
|  | Gemmeiza-9 | 0.244j→m±0.035 | 0.274j→m±0.042 | 0.262b→g±0.040 | 0.293c→g±0.045 | 0.240gh±0.033 | 0.269h±0.041 |
|  | Giza-168 | 0.250ij±0.036 | 0.280j±0.043 | 0.254g→j±0.037 | 0.284hij±0.044 | 0.238ghi±0.033 | 0.268hi±0.040 |
|  | Sids-14 | 0.272a→d±0.042 | 0.304a→d±0.047 | 0.271a±0.042 | 0.303a±0.047 | 0.252bcd±0.036 | 0.282bcd±0.043 |
|  | SOKOLL | 0.275ab±0.043 | 0.306ab±0.048 | 0.269abc±0.041 | 0.300abc±0.046 | 0.288a±0.046 | 0.320a±0.050 |
|  | 18 SAWYT 19/20 | 0.276a±0.043 | 0.308a±0.048 | 0.270ab±0.042 | 0.301ab±0.047 | 0.231h→m±0.031 | 0.261i→m±0.039 |
| The data of three replicates ± SE (standard error) are shown.  Means followed by different letters under the same water regimes were significantly different according to Duncan’s Multiple Range Test (p≤ 0.05) | | | | | | | |
