# Supplementary material for: Improving drought tolerance in some wheat genotypes with foliar application of silicon nanoparticles in Al-Dawadmi, Saudi Arabia
Source: PeerJ. 2026 Feb 24;14:e20823. doi: 10.7717/peerj.20823 (PMC12947762; doi:10.7717/peerj.20823)
Supplement: Supplemental Information 5 — The data of three replicates ± SE (standard error) are shown. Means followed by different letters under the same water regimes were significantly different according to Duncan’s Multiple Range Test (p ≤ 0.05) [file peerj-14-20823-s005.docx]

Supplementary Table S4. Water use efficiency of eight wheat genotypes as affected by foliar application of silicon nanoparticles under well-watered, moderate and severe water stress conditions during winter seasons of 2022/2023 (1^st^) and 2023/2024 (2^nd^ )

| SiNPs | Water use efficiency | | | | | | |
| --- | --- | --- | --- | --- | --- | --- | --- |
|  | Genotypes | Well-watered | | Moderate | | Severe | |
|  |  | 1st | 2nd | 1st | 2nd | 1st | 2nd |
| SiNPs_0_ | Giza 171 | 4.857x±0.513 | 5.127w±0.341 | 4.812x±0.515 | 5.093x±0.337 | 4.598x±0.536 | 4.934x±0.323 |
|  | Sakha 95 | 4.985tu±0.503 | 5.224st±0.350 | 4.910uv±0.510 | 5.169uv±0.344 | 4.653vw±0.528 | 4.974uvw±0.326 |
|  | Misr 3 | 5.006t±0.502 | 5.241s±0.349 | 4.935u±0.505 | 5.187u±0.344 | 4.801s±0.517 | 5.086s±0.336 |
|  | Gemmeiza-9 | 5.099qr±0.499 | 5.312qr±0.357 | 5.163mn±0.494 | 5.360mn±0.362 | 5.079k±0.500 | 5.297jk±0.355 |
|  | Giza-168 | 5.204kl±0.494 | 5.392jkl±0.365 | 5.132o±0.497 | 5.337no±0.359 | 4.973p±0.503 | 5.216p±0.347 |
|  | Sids-14 | 5.317i±0.492 | 5.478i±0.375 | 5.260jk±0.494 | 5.435jk±0.370 | 5.215efg±0.496 | 5.401efg±0.366 |
|  | SOKOLL | 5.367gh±0.492 | 5.518gh±0.379 | 5.308hi±0.491 | 5.472hi±0.372 | 5.237def±0.492 | 5.417def±0.368 |
|  | 18 SAWYT 19/20 | 5.417def±0.491 | 5.556b→f±0.382 | 5.358def±0.494 | 5.511def±0.378 | 5.040l→o±0.503 | 5.268l→o±0.354 |
| SiNPs_100_ | Giza 171 | 4.914w±0.508 | 5.172v±0.343 | 4.850w±0.513 | 5.124w±0.338 | 4.666uv±0.532 | 4.986uv±0.327 |
|  | Sakha 95 | 5.084rs±0.500 | 5.136w±0.341 | 4.987st±0.504 | 5.227q→t±0.348 | 4.684u±0.528 | 4.998u±0.328 |
|  | Misr 3 | 5.137op±0.498 | 5.342nop±0.360 | 5.024qr±0.503 | 5.254qr±0.353 | 4.839r±0.515 | 5.115r±0.339 |
|  | Gemmeiza-9 | 5.159mno±0.496 | 5.357mno±0.364 | 5.235kl±0.494 | 5.417kl±0.368 | 5.111ij±0.496 | 5.321hij±0.358 |
|  | Giza-168 | 5.218jk±0.495 | 5.404jk±0.366 | 5.178m±0.495 | 5.372m±0.363 | 5.044lmn±0.501 | 5.271k→n±0.353 |
|  | Sids-14 | 5.391fg±0.493 | 5.536efg±0.379 | 5.325gh±0.493 | 5.485fgh±0.375 | 5.245de±0.495 | 5.424de±0.369 |
|  | SOKOLL | 5.426b→e±0.492 | 5.563a→e±0.383 | 5.371de±0.492 | 5.521cde±0.379 | 5.293bc±0.493 | 5.461bc±0.371 |
|  | 18 SAWYT 19/20 | 5.450ab±0.492 | 5.582abc±0.386 | 5.374d±0.491 | 5.523cd±0.378 | 5.063kl±0.499 | 5.284kl±0.354 |
| SiNPs_200_ | Giza 171 | 4.953v±0.506 | 5.202tu±0.345 | 5.012qrs±0.502 | 5.246qrs±0.350 | 5.320b±0.495 | 5.482b±0.375 |
|  | Sakha 95 | 5.122pq±0.497 | 5.329opq±0.359 | 5.025q±0.499 | 5.255q±0.351 | 4.726t±0.525 | 5.029t±0.331 |
|  | Misr 3 | 5.171mn±0.498 | 5.368lmn±0.363 | 5.079p±0.500 | 5.296p±0.357 | 4.889q±0.513 | 5.153q±0.343 |
|  | Gemmeiza-9 | 5.187lm±0.496 | 5.380klm±0.364 | 5.351d→g±0.491 | 5.505d→g±0.376 | 5.142h±0.498 | 5.345h±0.362 |
|  | Giza-168 | 5.242j±0.492 | 5.420j±0.368 | 5.277j±0.493 | 5.448ij±0.371 | 5.125hi±0.500 | 5.332hi±0.361 |
|  | Sids-14 | 5.430bcd±0.492 | 5.567a→d±0.383 | 5.423a±0.493 | 5.561a±0.384 | 5.257d±0.494 | 5.433cd±0.370 |
|  | SOKOLL | 5.450ab±0.492 | 5.582ab±0.385 | 5.408abc±0.492 | 5.549abc±0.382 | 5.546a±0.494 | 5.657a±0.393 |
|  | 18 SAWYT 19/20 | 5.460a±0.492 | 5.590a±0.386 | 5.413ab±0.490 | 5.553ab±0.381 | 5.053klm±0.501 | 5.277klm±0.355 |
| The data of three replicates ± SE (standard error) are shown.  Means followed by different letters under the same water regimes were significantly different according to Duncan’s Multiple Range Test (p≤ 0.05) | | | | | | | |
