# Supplementary material for: Improving drought tolerance in some wheat genotypes with foliar application of silicon nanoparticles in Al-Dawadmi, Saudi Arabia
Source: PeerJ. 2026 Feb 24;14:e20823. doi: 10.7717/peerj.20823 (PMC12947762; doi:10.7717/peerj.20823)
Supplement: Supplemental Information 6 — The data of three replicates ± SE (standard error) are shown. Means followed by different letters under the same water regimes were significantly different according to Duncan’s Multiple Range Test (p ≤ 0.05) [file peerj-14-20823-s006.docx]

Supplementary Table S5. Intercellular CO_2_ of eight wheat genotypes as affected by foliar application of silicon nanoparticles under well-watered, moderate and severe water stress conditions during winter seasons of 2022/2023 (1^st^) and 2023/2024 (2^nd^ )

| SiNPs | Intercellular CO2 | | | | | | |
| --- | --- | --- | --- | --- | --- | --- | --- |
|  | Genotypes | Well-watered | | Moderate | | Severe | |
|  |  | 1^st^ | 2^nd^ | 1^st^ | 2^nd^ | 1^st^ | 2^nd^ |
| SiNPs_0_ | Giza 171 | 191.92v±24.23 | 199.82v±31.16 | 188.71v±23.57 | 196.40w±30.64 | 174.63t±20.07 | 181.84u±28.43 |
|  | Sakha 95 | 201.97stu±26.98 | 210.23st±32.97 | 195.97s→v±25.26 | 204.12tuv±31.94 | 178.04t±20.98 | 185.26tu±28.89 |
|  | Misr 3 | 203.75rst±27.37 | 212.25s±33.55 | 197.93q→u±25.95 | 206.14stu±32.52 | 187.90qrs±23.38 | 195.68qrs±30.63 |
|  | Gemmeiza-9 | 211.81m→r±29.31 | 220.70n→r±34.96 | 217.82lmn±31.07 | 226.80mn±36.04 | 210.04h→k±28.90 | 218.89h→k±34.71 |
|  | Giza-168 | 221.87jkl±32.11 | 231.11jkl±36.86 | 214.89mno±30.17 | 223.93mno±35.57 | 201.01l→p±26.80 | 209.37m→p±33.12 |
|  | Sids-14 | 233.82ghi±35.18 | 243.52hi±39.04 | 227.68h→k±33.57 | 237.22h→k±37.95 | 222.96c→g±32.12 | 232.37d→g±37.03 |
|  | SOKOLL | 239.64d→h±36.65 | 249.62fgh±40.16 | 232.86d→i±34.99 | 242.65f→i±39.16 | 225.27c→f±33.11 | 234.53c→f±37.44 |
|  | 18 SAWYT 19/20 | 245.65a→f±38.44 | 255.94a→f±41.62 | 238.48a→f±36.14 | 248.55b→f±39.95 | 206.63i→o±27.93 | 215.27j→o±33.81 |
| SiNPs_100_ | Giza 171 | 196.29tuv±25.37 | 204.52tuv±32.22 | 191.44uv±24.13 | 199.49uvw±31.41 | 178.81t±20.87 | 186.34tu±29.08 |
|  | Sakha 95 | 210.52n→s±29.00 | 200.71uv±31.42 | 202.11p→t±26.79 | 210.63q→t±33.25 | 179.96st±21.34 | 187.41tu±29.27 |
|  | Misr 3 | 215.38k→p±30.26 | 224.46l→p±35.67 | 205.19pqr±27.65 | 213.65pqr±33.51 | 190.63qr±23.94 | 198.55qr±31.03 |
|  | Gemmeiza-9 | 217.49j→o±30.94 | 226.41k→o±35.75 | 225.08i→l±32.80 | 234.53jkl±37.44 | 212.97hij±29.80 | 221.77hij±35.17 |
|  | Giza-168 | 223.31jk±32.39 | 232.73jk±37.17 | 219.27klm±31.34 | 228.42lm±36.35 | 206.96i→n±28.05 | 215.66j→n±34.10 |
|  | Sids-14 | 242.37b→g±37.25 | 252.71d→g±40.99 | 234.79d→h±35.36 | 244.59d→h±39.25 | 226.04b→e±32.98 | 235.60cde±37.65 |
|  | SOKOLL | 246.74a→e±38.45 | 257.21a→e±41.82 | 240.12a→e±36.74 | 250.16a→e±40.27 | 231.22bc±34.40 | 241.03bc±38.85 |
|  | 18 SAWYT 19/20 | 249.82ab±39.32 | 260.23abc±42.12 | 240.45a→d±36.88 | 250.56a→d±40.57 | 208.60h→l±28.63 | 217.28i→l±34.40 |
| SiNPs_200_ | Giza 171 | 199.37tuv±26.22 | 207.75stu±32.82 | 204.23p→s±27.47 | 212.78p→s±33.65 | 234.11b±34.95 | 244.05b±39.14 |
|  | Sakha 95 | 213.93l→q±29.98 | 222.85m→q±35.37 | 205.38pq±27.96 | 213.86pq±33.85 | 182.70rst±21.89 | 190.29st±29.66 |
|  | Misr 3 | 218.59j→n±30.94 | 227.88j→n±36.24 | 210.04nop±28.90 | 218.68op±34.37 | 194.33pq±24.68 | 202.51pq±31.65 |
|  | Gemmeiza-9 | 220.23j→m±31.53 | 229.50j→m±36.55 | 237.71a→g±36.28 | 247.68c→g±40.07 | 215.86gh±30.35 | 224.79h±35.44 |
|  | Giza-168 | 225.76ij±33.20 | 235.07j±37.54 | 229.45g→j±33.99 | 239.02hij±38.23 | 214.22hi±29.76 | 223.18hi±35.13 |
|  | Sids-14 | 247.22a→d±38.54 | 257.75a→d±41.92 | 246.42a±38.30 | 256.81a±41.51 | 227.33bcd±33.30 | 236.87bcd±37.82 |
|  | SOKOLL | 249.82ab±39.32 | 260.44ab±42.44 | 244.49abc±37.93 | 254.66abc±41.09 | 262.74a±42.61 | 273.92a±44.96 |
|  | 18 SAWYT 19/20 | 251.11a±39.65 | 261.70a±42.65 | 245.16ab±38.34 | 255.41ab±41.51 | 207.79h→m±28.42 | 216.34i→m±34.02 |
| The data of three replicates ± SE (standard error) are shown.  Means followed by different letters under the same water regimes were significantly different according to Duncan’s Multiple Range Test (p≤ 0.05) | | | | | | | |
