# Supplementary material for: Improving drought tolerance in some wheat genotypes with foliar application of silicon nanoparticles in Al-Dawadmi, Saudi Arabia
Source: PeerJ. 2026 Feb 24;14:e20823. doi: 10.7717/peerj.20823 (PMC12947762; doi:10.7717/peerj.20823)
Supplement: Supplemental Information 7 — The data of three replicates ± SE (standard error) are shown. Means followed by different letters under the same water regimes were significantly different according to Duncan’s Multiple Range Test (p ≤ 0.05) [file peerj-14-20823-s007.docx]

Supplementary Table S6. Relative water content of eight wheat genotypes as affected by foliar application of silicon nanoparticles under well-watered, moderate and severe water stress conditions during winter seasons of 2022/2023 (1^st^) and 2023/2024 (2^nd^ )

| SiNPs | Relative Water Content | | | | | | |
| --- | --- | --- | --- | --- | --- | --- | --- |
|  | Genotypes | Well-watered | | Moderate | | Severe | |
|  |  | 1^st^ | 2^nd^ | 1^st^ | 2^nd^ | 1^st^ | 2^nd^ |
| SiNPs_0_ | Giza 171 | 63.23v±8.05 | 67.08v±10.43 | 62.05v±7.82 | 65.94w±10.26 | 56.91t±6.57 | 61.11u±9.53 |
|  | Sakha 95 | 66.90stu±9.05 | 70.54st±11.03 | 64.70s→v±8.42 | 68.51tuv±10.69 | 58.16t±6.89 | 62.24tu±9.68 |
|  | Misr 3 | 67.54rst±9.19 | 71.21s±11.23 | 65.42q→u±8.67 | 69.18stu±10.88 | 61.76qrs±7.75 | 65.70qrs±10.26 |
|  | Gemmeiza-9 | 70.49m→r±9.88 | 74.01n→r±11.69 | 72.68lmn±10.52 | 76.04mn±12.05 | 69.84h→k±9.74 | 73.42h→k±11.61 |
|  | Giza-168 | 74.16jkl±10.90 | 77.47jkl±12.32 | 71.61mno±10.20 | 75.09mno±11.89 | 66.55l→p±8.98 | 70.25m→p±11.08 |
|  | Sids-14 | 78.53ghi±12.01 | 81.60hi±13.04 | 76.28h→k±11.43 | 79.50h→k±12.68 | 74.56c→g±10.90 | 77.89d→g±12.38 |
|  | SOKOLL | 80.65d→h±12.55 | 83.63fgh±13.42 | 78.17d→i±11.95 | 81.31f→i±13.08 | 75.40c→f±11.26 | 78.61c→f±12.51 |
|  | 18 SAWYT 19/20 | 82.84a→f±13.20 | 85.72a→f±13.90 | 80.23a→f±12.37 | 83.27b→f±13.35 | 68.60i→o±9.39 | 72.21j→o±11.31 |
| SiNPs_100_ | Giza 171 | 64.82tuv±8.46 | 68.64tuv±10.78 | 63.05uv±8.02 | 66.97uvw±10.51 | 58.44t±6.85 | 62.60tu±9.74 |
|  | Sakha 95 | 70.02n→s±9.77 | 67.37uv±10.52 | 66.95p→t±8.98 | 70.67q→t±11.13 | 58.86st±7.02 | 62.96tu±9.81 |
|  | Misr 3 | 71.79k→p±10.23 | 75.27l→p±11.93 | 68.07pqr±9.29 | 71.67pqr±11.21 | 62.76qr±7.95 | 66.66qr±10.39 |
|  | Gemmeiza-9 | 72.56j→o±10.48 | 75.91k→o±11.95 | 75.33i→l±11.15 | 78.61jkl±12.51 | 70.91hij±10.06 | 74.37hij±11.76 |
|  | Giza-168 | 74.69jk±11.00 | 78.01jk±12.42 | 73.21klm±10.62 | 76.58lm±12.15 | 68.72i→n±9.43 | 72.34j→n±11.41 |
|  | Sids-14 | 81.65b→g±12.77 | 84.65d→g±13.69 | 78.88d→h±12.08 | 81.95d→h±13.11 | 75.69b→e±11.22 | 78.97cde±12.58 |
|  | SOKOLL | 83.24a→e±13.20 | 86.14a→e±13.96 | 80.82a→e±12.58 | 83.80a→e±13.45 | 77.58bc±11.73 | 80.77bc±12.98 |
|  | 18 SAWYT 19/20 | 84.37ab±13.52 | 87.15abc±14.07 | 80.94a→d±12.63 | 83.94a→d±13.55 | 69.32h→l±9.64 | 72.88i→l±11.51 |
| SiNPs_200_ | Giza 171 | 65.95tuv±8.77 | 69.71stu±10.98 | 67.72p→s±9.22 | 71.39p→s±11.26 | 78.63b±11.93 | 81.77b±13.08 |
|  | Sakha 95 | 71.26l→q±10.13 | 74.73m→q±11.83 | 68.14pq±9.40 | 71.74pq±11.33 | 59.86rst±7.21 | 63.91st±9.94 |
|  | Misr 3 | 72.96j→n±10.48 | 76.40j→n±12.12 | 69.84nop±9.74 | 73.35op±11.50 | 64.11pq±8.22 | 67.97pq±10.59 |
|  | Gemmeiza-9 | 73.56j→m±10.69 | 76.94j→m±12.22 | 79.95a→g±12.41 | 82.98c→g±13.39 | 71.97gh±10.26 | 75.37h±11.85 |
|  | Giza-168 | 75.58ij±11.30 | 78.79j±12.55 | 76.93g→j±11.58 | 80.10hij±12.78 | 71.37hi±10.05 | 74.84hi±11.75 |
|  | Sids-14 | 83.42a→d±13.24 | 86.32a→d±14.00 | 83.12a±13.15 | 86.01a±13.86 | 76.16bcd±11.33 | 79.39bcd±12.64 |
|  | SOKOLL | 84.37ab±13.52 | 87.22ab±14.17 | 82.42abc±13.02 | 85.30abc±13.72 | 89.09a±14.72 | 91.70a±15.01 |
|  | 18 SAWYT 19/20 | 84.84a±13.64 | 87.64a±14.24 | 82.67ab±13.17 | 85.55ab±13.86 | 69.02h→m±9.56 | 72.57i→m±11.38 |
| The data of three replicates ± SE (standard error) are shown.  Means followed by different letters under the same water regimes were significantly different according to Duncan’s Multiple Range Test (p≤ 0.05) | | | | | | | |
