# Supplementary material for: Improving drought tolerance in some wheat genotypes with foliar application of silicon nanoparticles in Al-Dawadmi, Saudi Arabia
Source: PeerJ. 2026 Feb 24;14:e20823. doi: 10.7717/peerj.20823 (PMC12947762; doi:10.7717/peerj.20823)
Supplement: Supplemental Information 8 — The data of three replicates ± SE (standard error) are shown. Means followed by different letters under the same water regimes were significantly different according to Duncan’s Multiple Range Test (p ≤ 0.05) [file peerj-14-20823-s008.docx]

Supplementary Table S7. Chlorophyll a of eight wheat genotypes as affected by foliar application of silicon nanoparticles under well-watered, moderate and severe water stress conditions during winter seasons of 2022/2023 (1^st^) and 2023/2024 (2^nd^ )

| SiNPs | Chlorophyll a | | | | | | |
| --- | --- | --- | --- | --- | --- | --- | --- |
|  | Genotypes | Well-watered | | Moderate | | Severe | |
|  |  | 1^st^ | 2^nd^ | 1^st^ | 2^nd^ | 1^st^ | 2^nd^ |
| SiNPs_0_ | Giza 171 | 2.072v±0.270 | 1.933v±0.330 | 2.032v±0.260 | 1.891w±0.320 | 1.856t±0.210 | 1.709u±0.290 |
|  | Sakha 95 | 2.198stu±0.300 | 2.064st±0.350 | 2.123s→v±0.280 | 1.987tuv±0.340 | 1.899t±0.230 | 1.752tu±0.300 |
|  | Misr 3 | 2.220rst±0.300 | 2.089s±0.360 | 2.148q→u±0.290 | 2.012stu±0.340 | 2.022qrs±0.250 | 1.882qrs±0.320 |
|  | Gemmeiza-9 | 2.321m→r±0.330 | 2.194n→r±0.380 | 2.396lmn±0.350 | 2.271mn±0.390 | 2.299h→k±0.320 | 2.172h→k±0.370 |
|  | Giza-168 | 2.447jkl±0.360 | 2.325jkl±0.400 | 2.360mno±0.340 | 2.235mno±0.380 | 2.186l→p±0.300 | 2.053m→p±0.350 |
|  | Sids-14 | 2.596ghi±0.400 | 2.480hi±0.430 | 2.519h→k±0.380 | 2.401h→k±0.420 | 2.460c→g±0.360 | 2.340d→g±0.400 |
|  | SOKOLL | 2.669d→h±0.420 | 2.556fgh±0.440 | 2.584d→i±0.400 | 2.469f→i±0.430 | 2.489c→f±0.370 | 2.367c→f±0.410 |
|  | 18 SAWYT 19/20 | 2.744a→f±0.440 | 2.635a→f±0.460 | 2.654a→f±0.410 | 2.542b→f±0.440 | 2.256i→o±0.310 | 2.126j→o±0.360 |
| SiNPs_100_ | Giza 171 | 2.127tuv±0.280 | 1.992tuv±0.340 | 2.066uv±0.260 | 1.929uvw±0.330 | 1.909t±0.220 | 1.765tu±0.300 |
|  | Sakha 95 | 2.305n→s±0.320 | 1.945uv±0.330 | 2.200p→t±0.300 | 2.069q→t±0.350 | 1.923st±0.230 | 1.778tu±0.300 |
|  | Misr 3 | 2.366k→p±0.340 | 2.241l→p±0.390 | 2.238pqr±0.310 | 2.106pqr±0.360 | 2.056qr±0.260 | 1.918qr±0.320 |
|  | Gemmeiza-9 | 2.392j→o±0.350 | 2.266k→o±0.390 | 2.487i→l±0.370 | 2.367jkl±0.410 | 2.336hij±0.330 | 2.208hij±0.380 |
|  | Giza-168 | 2.465jk±0.370 | 2.345jk±0.400 | 2.414klm±0.350 | 2.291lm±0.390 | 2.260i→n±0.310 | 2.131j→n±0.360 |
|  | Sids-14 | 2.703b→g±0.430 | 2.594d→g±0.460 | 2.608d→h±0.400 | 2.493d→h±0.430 | 2.499b→e±0.370 | 2.381cde±0.410 |
|  | SOKOLL | 2.758a→e±0.440 | 2.651a→e±0.470 | 2.675a→e±0.420 | 2.563a→e±0.450 | 2.564bc±0.390 | 2.449bc±0.430 |
|  | 18 SAWYT 19/20 | 2.796ab±0.450 | 2.688abc±0.470 | 2.679a→d±0.420 | 2.568a→d±0.450 | 2.281h→l±0.320 | 2.152i→l±0.370 |
| SiNPs_200_ | Giza 171 | 2.166tuv±0.290 | 2.033stu±0.350 | 2.226p→s±0.300 | 2.095p→s±0.360 | 2.600b±0.400 | 2.486b±0.430 |
|  | Sakha 95 | 2.348l→q±0.340 | 2.221m→q±0.380 | 2.241pq±0.310 | 2.109pq±0.360 | 1.957rst±0.240 | 1.814st±0.310 |
|  | Misr 3 | 2.406j→n±0.350 | 2.284j→n±0.390 | 2.299nop±0.320 | 2.169op±0.370 | 2.103pq±0.270 | 1.967pq±0.330 |
|  | Gemmeiza-9 | 2.426j→m±0.350 | 2.304j→m±0.400 | 2.645a→g±0.410 | 2.532c→g±0.440 | 2.372gh±0.340 | 2.246h±0.380 |
|  | Giza-168 | 2.495ij±0.380 | 2.374j±0.410 | 2.542g→j±0.390 | 2.423hij±0.420 | 2.351hi±0.330 | 2.225hi±0.380 |
|  | Sids-14 | 2.764a→d±0.440 | 2.657a→d±0.470 | 2.754a±0.440 | 2.646a±0.460 | 2.515bcd±0.380 | 2.396bcd±0.410 |
|  | SOKOLL | 2.796ab±0.450 | 2.691ab±0.470 | 2.729abc±0.430 | 2.619abc±0.460 | 2.958a±0.490 | 2.860a±0.510 |
|  | 18 SAWYT 19/20 | 2.812a±0.460 | 2.707a±0.480 | 2.738ab±0.440 | 2.628ab±0.460 | 2.271h→m±0.320 | 2.140i→m±0.360 |
| The data of three replicates ± SE (standard error) are shown.  Means followed by different letters under the same water regimes were significantly different according to Duncan’s Multiple Range Test (p≤ 0.05) | | | | | | | |
