# Supplementary material for: Improving drought tolerance in some wheat genotypes with foliar application of silicon nanoparticles in Al-Dawadmi, Saudi Arabia
Source: PeerJ. 2026 Feb 24;14:e20823. doi: 10.7717/peerj.20823 (PMC12947762; doi:10.7717/peerj.20823)
Supplement: Supplemental Information 9 — The data of three replicates ± SE (standard error) are shown. Means followed by different letters under the same water regimes were significantly different according to Duncan’s Multiple Range Test (p ≤ 0.05) [file peerj-14-20823-s009.docx]

Supplementary Table S8. Chlorophyll b of eight wheat genotypes as affected by foliar application of silicon nanoparticles under well-watered, moderate and severe water stress conditions during winter seasons of 2022/2023 (1^st^) and 2023/2024 (2^nd^ )

| SiNPs | Chlorophyll b | | | | | | |
| --- | --- | --- | --- | --- | --- | --- | --- |
|  | Genotypes | Well-watered | | Moderate | | Severe | |
|  |  | 1^st^ | 2^nd^ | 1^st^ | 2^nd^ | 1^st^ | 2^nd^ |
| SiNPs_0_ | Giza 171 | 0.484v±0.060 | 0.464v±0.080 | 0.473v±0.060 | 0.453w±0.080 | 0.426t±0.050 | 0.404u±0.070 |
|  | Sakha 95 | 0.518stu±0.070 | 0.499st±0.090 | 0.498s→v±0.070 | 0.479tuv±0.080 | 0.438t±0.050 | 0.416tu±0.070 |
|  | Misr 3 | 0.524rst±0.070 | 0.506s±0.090 | 0.504q→u±0.070 | 0.486stu±0.090 | 0.471qrs±0.060 | 0.451qrs±0.080 |
|  | Gemmeiza-9 | 0.551m→r±0.080 | 0.534n→r±0.100 | 0.571lmn±0.090 | 0.555mn±0.100 | 0.545h→k±0.080 | 0.528h→k±0.090 |
|  | Giza-168 | 0.584jkl±0.090 | 0.569jkl±0.100 | 0.561mno±0.080 | 0.545mno±0.100 | 0.515l→p±0.070 | 0.496m→p±0.090 |
|  | Sids-14 | 0.624ghi±0.100 | 0.611hi±0.110 | 0.604h→k±0.090 | 0.590h→k±0.110 | 0.588c→g±0.090 | 0.573d→g±0.100 |
|  | SOKOLL | 0.644d→h±0.100 | 0.631fgh±0.110 | 0.621d→i±0.100 | 0.608f→i±0.110 | 0.596c→f±0.090 | 0.581c→f±0.100 |
|  | 18 SAWYT 19/20 | 0.664a→f±0.110 | 0.652a→f±0.120 | 0.640a→f±0.100 | 0.627b→f±0.110 | 0.533i→o±0.070 | 0.516j→o±0.090 |
| SiNPs_100_ | Giza 171 | 0.499tuv±0.070 | 0.480tuv±0.090 | 0.483uv±0.060 | 0.463uvw±0.080 | 0.440t±0.050 | 0.419tu±0.070 |
|  | Sakha 95 | 0.547n→s±0.080 | 0.467uv±0.080 | 0.518p→t±0.070 | 0.501q→t±0.090 | 0.444st±0.050 | 0.423tu±0.070 |
|  | Misr 3 | 0.563k→p±0.080 | 0.547l→p±0.100 | 0.529pqr±0.070 | 0.511pqr±0.090 | 0.480qr±0.060 | 0.460qr±0.080 |
|  | Gemmeiza-9 | 0.570j→o±0.080 | 0.553k→o±0.100 | 0.595i→l±0.090 | 0.581jkl±0.100 | 0.555hij±0.080 | 0.538hij±0.100 |
|  | Giza-168 | 0.589jk±0.090 | 0.575jk±0.100 | 0.576klm±0.090 | 0.560lm±0.100 | 0.535i→n±0.080 | 0.517j→n±0.090 |
|  | Sids-14 | 0.653b→g±0.110 | 0.641d→g±0.120 | 0.628d→h±0.100 | 0.614d→h±0.110 | 0.598b→e±0.090 | 0.584cde±0.100 |
|  | SOKOLL | 0.668a→e±0.110 | 0.656a→e±0.120 | 0.646a→e±0.100 | 0.633a→e±0.110 | 0.616bc±0.100 | 0.602bc±0.110 |
|  | 18 SAWYT 19/20 | 0.678ab±0.110 | 0.667abc±0.120 | 0.647a→d±0.100 | 0.634a→d±0.120 | 0.540h→l±0.080 | 0.523i→l±0.090 |
| SiNPs_200_ | Giza 171 | 0.509tuv±0.070 | 0.491stu±0.090 | 0.525p→s±0.070 | 0.508p→s±0.090 | 0.625b±0.100 | 0.612b±0.110 |
|  | Sakha 95 | 0.558l→q±0.080 | 0.541m→q±0.100 | 0.529pq±0.070 | 0.511pq±0.090 | 0.453rst±0.060 | 0.432st±0.080 |
|  | Misr 3 | 0.574j→n±0.080 | 0.558j→n±0.100 | 0.545nop±0.080 | 0.528op±0.090 | 0.492pq±0.060 | 0.473pq±0.080 |
|  | Gemmeiza-9 | 0.579j→m±0.090 | 0.564j→m±0.100 | 0.638a→g±0.100 | 0.625c→g±0.110 | 0.564gh±0.080 | 0.548h±0.100 |
|  | Giza-168 | 0.597ij±0.090 | 0.582j±0.100 | 0.610g→j±0.090 | 0.596hij±0.110 | 0.559hi±0.080 | 0.543hi±0.100 |
|  | Sids-14 | 0.669a→d±0.110 | 0.658a→d±0.120 | 0.667a±0.110 | 0.655a±0.120 | 0.603bcd±0.090 | 0.588bcd±0.110 |
|  | SOKOLL | 0.678ab±0.110 | 0.667ab±0.120 | 0.660abc±0.110 | 0.648abc±0.120 | 0.721a±0.120 | 0.712a±0.130 |
|  | 18 SAWYT 19/20 | 0.682a±0.110 | 0.671a±0.120 | 0.662ab±0.110 | 0.650ab±0.120 | 0.537h→m±0.080 | 0.520i→m±0.090 |
| The data of three replicates ± SE (standard error) are shown.  Means followed by different letters under the same water regimes were significantly different according to Duncan’s Multiple Range Test (p≤ 0.05) | | | | | | | |
