# Supplementary material for: Improving drought tolerance in some wheat genotypes with foliar application of silicon nanoparticles in Al-Dawadmi, Saudi Arabia
Source: PeerJ. 2026 Feb 24;14:e20823. doi: 10.7717/peerj.20823 (PMC12947762; doi:10.7717/peerj.20823)
Supplement: Supplemental Information 10 — The data of three replicates ± SE (standard error) are shown. Means followed by different letters under the same water regimes were significantly different according to Duncan’s Multiple Range Test (p ≤ 0.05) [file peerj-14-20823-s010.docx]

Supplementary Table S9. Carotenoid of eight wheat genotypes as affected by foliar application of silicon nanoparticles under well-watered, moderate and severe water stress conditions during winter seasons of 2022/2023 (1^st^) and 2023/2024 (2^nd^ )

| SiNPs | Carotenoid | | | | | | |
| --- | --- | --- | --- | --- | --- | --- | --- |
|  | Genotypes | Well-watered | | Moderate | | Severe | |
|  |  | 1st | 2nd | 1st | 2nd | 1st | 2nd |
| SiNPs_0_ | Giza 171 | 0.379v±0.050 | 0.433v±0.070 | 0.373v±0.050 | 0.427w±0.060 | 0.347t±0.040 | 0.400u±0.060 |
|  | Sakha 95 | 0.398stu±0.050 | 0.453st±0.070 | 0.387s→v±0.050 | 0.441tuv±0.070 | 0.353t±0.040 | 0.406tu±0.060 |
|  | Misr 3 | 0.401rst±0.050 | 0.457s±0.070 | 0.390q→u±0.050 | 0.445stu±0.070 | 0.371qrs±0.050 | 0.426qrs±0.060 |
|  | Gemmeiza-9 | 0.416m→r±0.060 | 0.473n→r±0.070 | 0.428lmn±0.060 | 0.484mn±0.070 | 0.413h→k±0.060 | 0.469h→k±0.070 |
|  | Giza-168 | 0.435jkl±0.060 | 0.492jkl±0.080 | 0.422mno±0.060 | 0.479mno±0.070 | 0.396l→p±0.050 | 0.451m→p±0.070 |
|  | Sids-14 | 0.458ghi±0.070 | 0.515hi±0.080 | 0.446h→k±0.070 | 0.504ijk±0.080 | 0.437c→g±0.060 | 0.494d→g±0.080 |
|  | SOKOLL | 0.469d→h±0.070 | 0.527fgh±0.080 | 0.456d→i±0.070 | 0.514f→i±0.080 | 0.442c→f±0.060 | 0.499c→f±0.080 |
|  | 18 SAWYT 19/20 | 0.480a→f±0.070 | 0.539a→f±0.080 | 0.466a→f±0.070 | 0.525b→f±0.080 | 0.407i→o±0.050 | 0.462j→o±0.070 |
| SiNPs_100_ | Giza 171 | 0.387tuv±0.050 | 0.442tuv±0.070 | 0.378uv±0.050 | 0.433uvw±0.070 | 0.354t±0.040 | 0.408tu±0.060 |
|  | Sakha 95 | 0.414n→s±0.060 | 0.435uv±0.070 | 0.398p→t±0.050 | 0.454q→t±0.070 | 0.357st±0.040 | 0.410tu±0.060 |
|  | Misr 3 | 0.423k→p±0.060 | 0.480l→p±0.070 | 0.404pqr±0.050 | 0.459pqr±0.070 | 0.377qr±0.050 | 0.431qr±0.070 |
|  | Gemmeiza-9 | 0.427j→o±0.060 | 0.483k→o±0.070 | 0.441i→l±0.060 | 0.499jkl±0.080 | 0.419hij±0.060 | 0.475hij±0.070 |
|  | Giza-168 | 0.438jk±0.060 | 0.495jk±0.080 | 0.430klm±0.060 | 0.487lm±0.070 | 0.407i→n±0.050 | 0.463j→n±0.070 |
|  | Sids-14 | 0.474b→g±0.070 | 0.533d→g±0.080 | 0.460d→h±0.070 | 0.517d→h±0.080 | 0.443b→e±0.060 | 0.501cde±0.080 |
|  | SOKOLL | 0.482a→e±0.070 | 0.541a→e±0.080 | 0.470a→e±0.070 | 0.528a→e±0.080 | 0.453bc±0.070 | 0.511bc±0.080 |
|  | 18 SAWYT 19/20 | 0.488ab±0.080 | 0.547abc±0.090 | 0.470a→d±0.070 | 0.529a→d±0.080 | 0.410h→l±0.060 | 0.466i→l±0.070 |
| SiNPs_200_ | Giza 171 | 0.393tuv±0.050 | 0.448stu±0.070 | 0.402p→s±0.050 | 0.458p→s±0.070 | 0.458b±0.070 | 0.516b±0.080 |
|  | Sakha 95 | 0.420l→q±0.060 | 0.477m→q±0.070 | 0.404pq±0.050 | 0.460pq±0.070 | 0.362rst±0.040 | 0.415st±0.060 |
|  | Misr 3 | 0.429j→n±0.060 | 0.486j→n±0.070 | 0.413nop±0.060 | 0.469op±0.070 | 0.384pq±0.050 | 0.438pq±0.070 |
|  | Gemmeiza-9 | 0.432j→m±0.060 | 0.489j→m±0.080 | 0.465a→g±0.070 | 0.523c→g±0.080 | 0.424gh±0.060 | 0.480h±0.070 |
|  | Giza-168 | 0.443ij±0.060 | 0.500j±0.080 | 0.449g→j±0.070 | 0.507hij±0.080 | 0.421hi±0.060 | 0.477hi±0.070 |
|  | Sids-14 | 0.483a→d±0.070 | 0.542a→d±0.080 | 0.481a±0.070 | 0.540a±0.080 | 0.446bcd±0.060 | 0.503bcd±0.080 |
|  | SOKOLL | 0.488ab±0.080 | 0.547ab±0.090 | 0.478abc±0.070 | 0.536abc±0.080 | 0.512a±0.080 | 0.572a±0.090 |
|  | 18 SAWYT 19/20 | 0.490a±0.080 | 0.550a±0.090 | 0.479ab±0.070 | 0.538ab±0.080 | 0.409h→m±0.060 | 0.464i→m±0.070 |
| The data of three replicates ± SE (standard error) are shown.  Means followed by different letters under the same water regimes were significantly different according to Duncan’s Multiple Range Test (p≤ 0.05) | | | | | | | |
