# Supplementary material for: Improving drought tolerance in some wheat genotypes with foliar application of silicon nanoparticles in Al-Dawadmi, Saudi Arabia
Source: PeerJ. 2026 Feb 24;14:e20823. doi: 10.7717/peerj.20823 (PMC12947762; doi:10.7717/peerj.20823)
Supplement: Supplemental Information 11 — The data of three replicates ± SE (standard error) are shown. Means followed by different letters under the same water regimes were significantly different according to Duncan’s Multiple Range Test (p ≤ 0.05) [file peerj-14-20823-s011.docx]

Supplementary Table S10. Total photosynthetic pigments of eight wheat genotypes as affected by foliar application of silicon nanoparticles under well-watered, moderate and severe water stress conditions during winter seasons of 2022/2023 (1^st^) and 2023/2024 (2^nd^ )

| SiNPs | Total photosynthetic pigments | | | | | | |
| --- | --- | --- | --- | --- | --- | --- | --- |
|  | Genotypes | Well-watered | | Moderate | | Severe | |
|  |  | 1st | 2nd | 1st | 2nd | 1st | 2nd |
| SiNPs_0_ | Giza 171 | 2.822v±0.350 | 2.884v±0.440 | 2.780v±0.340 | 2.839w±0.430 | 2.594t±0.300 | 2.647u±0.400 |
|  | Sakha 95 | 2.955stu±0.390 | 3.022st±0.460 | 2.875s→v±0.370 | 2.941tuv±0.450 | 2.639t±0.310 | 2.693tu±0.410 |
|  | Misr 3 | 2.978rst±0.400 | 3.048s±0.470 | 2.901q→u±0.380 | 2.968stu±0.460 | 2.769qrs±0.340 | 2.830qrs±0.430 |
|  | Gemmeiza-9 | 3.084m→r±0.420 | 3.160n→r±0.490 | 3.164lmn±0.440 | 3.240mn±0.500 | 3.061h→k±0.420 | 3.136h→k±0.490 |
|  | Giza-168 | 3.217jkl±0.460 | 3.297jkl±0.510 | 3.125mno±0.430 | 3.202mno±0.500 | 2.942l→p±0.390 | 3.010m→p±0.470 |
|  | Sids-14 | 3.374ghi±0.500 | 3.461hi±0.540 | 3.294h→k±0.480 | 3.378h→k±0.530 | 3.231c→g±0.460 | 3.314d→g±0.520 |
|  | SOKOLL | 3.451d→h±0.520 | 3.541fgh±0.560 | 3.362d→i±0.500 | 3.449f→i±0.540 | 3.262c→f±0.470 | 3.342c→f±0.520 |
|  | 18 SAWYT 19/20 | 3.530a→f±0.540 | 3.624a→f±0.570 | 3.436a→f±0.510 | 3.527b→f±0.550 | 3.016i→o±0.400 | 3.088j→o±0.470 |
| SiNPs_100_ | Giza 171 | 2.880tuv±0.370 | 2.946tuv±0.450 | 2.816uv±0.350 | 2.880uvw±0.440 | 2.649t±0.310 | 2.707tu±0.410 |
|  | Sakha 95 | 3.067n→s±0.420 | 2.896uv±0.440 | 2.956p→t±0.390 | 3.027q→t±0.470 | 2.664st±0.310 | 2.721tu±0.420 |
|  | Misr 3 | 3.131k→p±0.430 | 3.209l→p±0.500 | 2.997pqr±0.400 | 3.067pqr±0.470 | 2.805qr±0.350 | 2.868qr±0.440 |
|  | Gemmeiza-9 | 3.159j→o±0.440 | 3.235k→o±0.500 | 3.259i→l±0.470 | 3.342jkl±0.520 | 3.100hij±0.430 | 3.174hij±0.490 |
|  | Giza-168 | 3.236jk±0.460 | 3.318jk±0.520 | 3.183klm±0.450 | 3.262lm±0.510 | 3.020i→n±0.400 | 3.093j→n±0.480 |
|  | Sids-14 | 3.487b→g±0.530 | 3.582d→g±0.570 | 3.387d→h±0.500 | 3.475d→h±0.540 | 3.272b→e±0.470 | 3.356cde±0.520 |
|  | SOKOLL | 3.545a→e±0.540 | 3.641a→e±0.580 | 3.457a→e±0.520 | 3.548a→e±0.560 | 3.340bc±0.490 | 3.428bc±0.540 |
|  | 18 SAWYT 19/20 | 3.585ab±0.550 | 3.681abc±0.580 | 3.462a→d±0.520 | 3.553a→d±0.560 | 3.042h→l±0.410 | 3.115i→l±0.480 |
| SiNPs_200_ | Giza 171 | 2.920tuv±0.380 | 2.989stu±0.460 | 2.984p→s±0.400 | 3.055p→s±0.470 | 3.378b±0.500 | 3.468b±0.540 |
|  | Sakha 95 | 3.112l→q±0.430 | 3.188m→q±0.490 | 3.000pq±0.400 | 3.070pq±0.470 | 2.700rst±0.320 | 2.759st±0.420 |
|  | Misr 3 | 3.174j→n±0.440 | 3.254j→n±0.510 | 3.061nop±0.420 | 3.133op±0.480 | 2.854pq±0.360 | 2.920pq±0.450 |
|  | Gemmeiza-9 | 3.195j→m±0.450 | 3.276j→m±0.510 | 3.426a→g±0.510 | 3.515c→g±0.550 | 3.138gh±0.440 | 3.214h±0.490 |
|  | Giza-168 | 3.268ij±0.470 | 3.349j±0.520 | 3.317g→j±0.480 | 3.401hij±0.530 | 3.116hi±0.430 | 3.192hi±0.490 |
|  | Sids-14 | 3.551a→d±0.540 | 3.648a→d±0.580 | 3.540a±0.540 | 3.636a±0.570 | 3.289bcd±0.470 | 3.373bcd±0.530 |
|  | SOKOLL | 3.585ab±0.550 | 3.684ab±0.590 | 3.515abc±0.540 | 3.607abc±0.570 | 3.756a±0.600 | 3.861a±0.620 |
|  | 18 SAWYT 19/20 | 3.602a±0.560 | 3.700a±0.590 | 3.524ab±0.540 | 3.617ab±0.570 | 3.031h→m±0.410 | 3.102i→m±0.480 |
| The data of three replicates ± SE (standard error) are shown.  Means followed by different letters under the same water regimes were significantly different according to Duncan’s Multiple Range Test (p≤ 0.05) | | | | | | | |
