# Supplementary material for: Improving drought tolerance in some wheat genotypes with foliar application of silicon nanoparticles in Al-Dawadmi, Saudi Arabia
Source: PeerJ. 2026 Feb 24;14:e20823. doi: 10.7717/peerj.20823 (PMC12947762; doi:10.7717/peerj.20823)
Supplement: Supplemental Information 12 — The data of three replicates ± SE (standard error) are shown. Means followed by different letters under the same water regimes were significantly different according to Duncan’s Multiple Range Test (p ≤ 0.05) [file peerj-14-20823-s012.docx]

Supplementary Table S11. Shoot length of eight wheat genotypes as affected by foliar application of silicon nanoparticles under well-watered, moderate and severe water stress conditions during winter seasons of 2022/2023 (1^st^) and 2023/2024 (2^nd^ )

| SiNPs | Shoot length | | | | | | |
| --- | --- | --- | --- | --- | --- | --- | --- |
|  | Genotypes | Well-wattered | | Moderate | | Severe | |
|  |  | 1^st^ | 2nd | 1st | 2nd | 1st | 2nd |
| SiNPs_0_ | Giza 171 | 94.69w±6.43 | 127.18v±7.47 | 92.88w±6.41 | 125.24w±7.18 | 84.98v±6.32 | 116.98u±6.12 |
|  | Sakha 95 | 100.33tu±6.84 | 133.09st±8.32 | 96.97uv±6.62 | 129.62tuv±7.80 | 86.89tuv±6.27 | 118.92tu±6.35 |
|  | Misr 3 | 101.33t±6.82 | 134.22s±8.63 | 98.06tu±6.62 | 130.76tu±8.11 | 92.43rs±6.51 | 124.82rs±7.10 |
|  | Gemmeiza-9 | 105.87pqr±7.07 | 139.02o→r±9.42 | 109.23mn±7.41 | 142.49mn±9.98 | 104.87ijk±7.08 | 137.99h→k±9.21 |
|  | Giza-168 | 111.51jkl±7.69 | 144.93jkl±10.33 | 107.60mno±7.28 | 140.85mno±9.68 | 99.79p±6.79 | 132.59nop±8.35 |
|  | Sids-14 | 118.23i±8.29 | 151.97hi±11.58 | 114.78ijk±8.00 | 148.39ijk±10.90 | 112.13d→g±7.61 | 145.65d→g±10.50 |
|  | SOKOLL | 121.49gh±8.64 | 155.44fgh±12.17 | 117.68ghi±8.23 | 151.47f→i±11.58 | 113.42def±7.87 | 146.87c→f±10.68 |
|  | 18 SAWYT 19/20 | 124.86b→f±9.11 | 159.01a→f±12.85 | 120.85def±8.48 | 154.83c→f±12.08 | 102.96k→o±6.96 | 135.95j→o±8.77 |
| SiNPs_100_ | Giza 171 | 97.15vw±6.52 | 129.84tuv±7.98 | 94.42vw±6.41 | 126.99vw±7.56 | 87.33tuv±6.32 | 119.53tu±6.42 |
|  | Sakha 95 | 105.14p→s±7.10 | 127.68v±7.50 | 100.41q→t±6.71 | 133.31q→t±8.50 | 87.97tu±6.34 | 120.14tu±6.50 |
|  | Misr 3 | 107.87m→p±7.31 | 141.16m→p±9.72 | 102.15pqr±6.89 | 135.03pqr±8.64 | 93.97qr±6.50 | 126.46qr±7.34 |
|  | Gemmeiza-9 | 109.05l→o±7.48 | 142.27k→o±9.79 | 113.32jkl±7.79 | 146.87jkl±10.68 | 106.51hij±7.18 | 139.63hij±9.50 |
|  | Giza-168 | 112.32jk±7.77 | 145.84jk±10.46 | 110.05m±7.49 | 143.40lm±10.11 | 103.14k→n±6.88 | 136.17j→n±8.95 |
|  | Sids-14 | 123.03d→g±8.73 | 157.18d→g±12.58 | 118.77fgh±8.34 | 152.58d→h±11.67 | 113.86cde±7.84 | 147.48cde±10.77 |
|  | SOKOLL | 125.49a→e±9.02 | 159.74a→e±13.05 | 121.77b→e±8.66 | 155.74b→e±12.22 | 116.77bc±8.06 | 150.56bc±11.45 |
|  | 18 SAWYT 19/20 | 127.22ab±9.30 | 161.46abc±13.24 | 121.95bcd±8.63 | 155.96a→d±12.41 | 104.05jkl±7.00 | 137.08i→l±9.08 |
| SiNPs_200_ | Giza 171 | 98.88tuv±6.69 | 131.67stu±8.23 | 101.60qrs±6.84 | 134.53p→s±8.67 | 118.40b±8.22 | 152.28b±11.62 |
|  | Sakha 95 | 107.05n→q±7.23 | 140.24n→q±9.59 | 102.24pq±6.94 | 135.14pq±8.75 | 89.51st±6.31 | 121.77st±6.71 |
|  | Misr 3 | 109.68k→n±7.39 | 143.10k→n±10.06 | 104.87op±7.08 | 137.89op±9.10 | 96.05q±6.53 | 128.71q±7.68 |
|  | Gemmeiza-9 | 110.59klm±7.54 | 144.01j→m±10.20 | 120.41d→g±8.53 | 154.33d→g±12.08 | 108.14h±7.34 | 141.35h±9.66 |
|  | Giza-168 | 113.69j±7.90 | 147.17j±10.72 | 115.77ij±8.03 | 149.42hij±11.13 | 107.23hi±7.20 | 140.44hi±9.53 |
|  | Sids-14 | 125.76a→d±9.05 | 160.04a→d±13.09 | 125.31a±9.06 | 159.52a±12.86 | 114.59cd±7.84 | 148.20cd±10.95 |
|  | SOKOLL | 127.22ab±9.30 | 161.56ab±13.32 | 124.22abc±8.95 | 158.30abc±12.68 | 134.48a±10.12 | 169.22a±14.73 |
|  | 18 SAWYT 19/20 | 127.94a±9.32 | 162.29a±13.52 | 124.59ab±9.08 | 158.71ab±12.81 | 103.60j→m±7.06 | 136.55j→m±8.85 |
| The data of three replicates ± SE (standard error) are shown.  Means followed by different letters under the same water regimes were significantly different according to Duncan’s Multiple Range Test (p≤ 0.05) | | | | | | | |
