# Supplementary material for: Improving drought tolerance in some wheat genotypes with foliar application of silicon nanoparticles in Al-Dawadmi, Saudi Arabia
Source: PeerJ. 2026 Feb 24;14:e20823. doi: 10.7717/peerj.20823 (PMC12947762; doi:10.7717/peerj.20823)
Supplement: Supplemental Information 13 [file peerj-14-20823-s013.docx]

Supplementary Table S12. Leaf area per plant of eight wheat genotypes as affected by foliar application of silicon nanoparticles under well-watered, moderate and severe water stress conditions during winter seasons of 2022/2023 (1^st^) and 2023/2024 (2^nd^ )

| SiNPs | Leaf area per plant | | | | | | |
| --- | --- | --- | --- | --- | --- | --- | --- |
|  | Genotypes | Well-watered | | Moderate | | Severe | |
|  |  | 1st | 2nd | 1st | 2nd | 1st | 2nd |
| SiNPs_0_ | Giza 171 | 316.17v±39.63 | 194.49v±34.83 | 311.43v±38.65 | 189.45w±33.96 | 290.69t±33.37 | 168.00u±30.28 |
|  | Sakha 95 | 330.98stu±43.73 | 209.84st±37.72 | 322.13s→v±41.16 | 200.84tuv±36.05 | 295.71t±34.75 | 173.04tu±31.08 |
|  | Misr 3 | 333.59rst±44.32 | 212.80s±38.64 | 325.02q→u±42.19 | 203.80stu±36.96 | 310.24qrs±38.35 | 188.39qrs±33.86 |
|  | Gemmeiza-9 | 345.48m→r±47.19 | 225.25n→r±40.95 | 354.33lmn±49.81 | 234.25mn±42.67 | 342.87h→k±46.59 | 222.60h→k±40.48 |
|  | Giza-168 | 360.29jkl±51.35 | 240.60jkl±43.92 | 350.02mno±48.47 | 230.01mno±41.88 | 329.56l→p±43.46 | 208.56m→p±37.89 |
|  | Sids-14 | 377.91ghi±55.89 | 258.88hi±47.44 | 368.86h→k±53.51 | 249.60h→k±45.66 | 361.91c→g±51.36 | 242.46d→g±44.25 |
|  | SOKOLL | 386.48d→h±58.07 | 267.88fgh±49.21 | 376.49d→i±55.62 | 257.60f→i±47.57 | 365.31c→f±52.84 | 245.63c→f±44.87 |
|  | 18 SAWYT 19/20 | 395.33a→f±60.72 | 277.19a→f±51.43 | 384.77a→f±57.33 | 266.29b→f±48.90 | 337.85i→o±45.13 | 217.25j→o±39.09 |
| SiNPs_100_ | Giza 171 | 322.61tuv±41.33 | 201.42tuv±36.50 | 315.46uv±39.49 | 194.01uvw±35.16 | 296.85t±34.58 | 174.63tu±31.38 |
|  | Sakha 95 | 343.58n→s±46.73 | 195.80uv±35.18 | 331.18p→t±43.46 | 210.42q→t±38.17 | 298.55st±35.29 | 176.21tu±31.68 |
|  | Misr 3 | 350.73k→p±48.60 | 230.80l→p±42.04 | 335.72pqr±44.73 | 214.87pqr±38.63 | 314.27qr±39.19 | 192.63qr±34.57 |
|  | Gemmeiza-9 | 353.85j→o±49.61 | 233.67k→o±42.20 | 365.03i→l±52.37 | 245.63jkl±44.87 | 347.18hij±47.92 | 226.84hij±41.26 |
|  | Giza-168 | 362.42jk±51.76 | 242.98jk±44.39 | 356.46klm±50.21 | 236.63lm±43.14 | 338.33i→n±45.32 | 217.84j→n±39.55 |
|  | Sids-14 | 390.50b→g±58.96 | 272.43d→g±50.48 | 379.33d→h±56.16 | 260.46d→h±47.76 | 366.45b→e±52.64 | 247.22cde±45.19 |
|  | SOKOLL | 396.95a→e±60.74 | 279.05a→e±51.79 | 387.19a→e±58.20 | 268.67a→e±49.37 | 374.08bc±54.75 | 255.22bc±47.10 |
|  | 18 SAWYT 19/20 | 401.49ab±62.02 | 283.50abc±52.31 | 387.67a→d±58.42 | 269.26a→d±49.85 | 340.74h→l±46.19 | 220.22i→l±40.02 |
| SiNPs_200_ | Giza 171 | 327.15tuv±42.60 | 206.18stu±37.42 | 334.30p→s±44.45 | 213.60p→s±38.79 | 378.33b±55.56 | 259.67b±47.60 |
|  | Sakha 95 | 348.60l→q±48.20 | 228.43m→q±41.57 | 336.00pq±45.18 | 215.18pq±39.10 | 302.58rst±36.11 | 180.45st±32.36 |
|  | Misr 3 | 355.46j→n±49.61 | 235.84j→n±42.98 | 342.87nop±46.59 | 222.29op±40.01 | 319.72pq±40.30 | 198.46pq±35.59 |
|  | Gemmeiza-9 | 357.88j→m±50.48 | 238.22j→m±43.45 | 383.64a→g±57.53 | 265.02c→g±49.02 | 351.44gh±48.74 | 231.29h±41.73 |
|  | Giza-168 | 366.02ij±52.97 | 246.43j±45.03 | 371.47g→j±54.13 | 252.26hij±46.15 | 349.02hi±47.87 | 228.91hi±41.26 |
|  | Sids-14 | 397.65a→d±60.87 | 279.85a→d±51.95 | 396.46a±60.52 | 278.47a±51.31 | 368.35bcd±53.12 | 249.08bcd±45.52 |
|  | SOKOLL | 401.49ab±62.02 | 283.81ab±52.74 | 393.63abc±59.97 | 275.29abc±50.68 | 420.52a±66.89 | 303.68a±56.71 |
|  | 18 SAWYT 19/20 | 403.39a±62.51 | 285.67a±53.11 | 394.62ab±60.58 | 276.40ab±51.27 | 339.55h→m±45.86 | 218.84i→m±39.40 |
| The data of three replicates ± SE (standard error) are shown.  Means followed by different letters under the same water regimes were significantly different according to Duncan’s Multiple Range Test (p≤ 0.05) | | | | | | | |
