# Supplementary material for: Improving drought tolerance in some wheat genotypes with foliar application of silicon nanoparticles in Al-Dawadmi, Saudi Arabia
Source: PeerJ. 2026 Feb 24;14:e20823. doi: 10.7717/peerj.20823 (PMC12947762; doi:10.7717/peerj.20823)
Supplement: Supplemental Information 14 — The data of three replicates ± SE (standard error) are shown. Means followed by different letters under the same water regimes were significantly different according to Duncan’s Multiple Range Test (p ≤ 0.05) [file peerj-14-20823-s014.docx]

Supplementary Table S13. No. of tillers per plant of eight wheat genotypes as affected by foliar application of silicon nanoparticles under well-watered, moderate and severe water stress conditions during winter seasons of 2022/2023 (1^st^) and 2023/2024 (2^nd^ )

| SiNPs | No. of tillers per plant | | | | | | |
| --- | --- | --- | --- | --- | --- | --- | --- |
|  | Genotypes | Well-watered | | Moderate | | Severe | |
|  |  | 1st | 2nd | 1st | 2^nd^ | 1st | 2nd |
| SiNPs_0_ | Giza 171 | 4.54v±0.57 | 4.48v±0.71 | 4.46v±0.56 | 4.40w±0.70 | 4.13t±0.47 | 4.05u±0.64 |
|  | Sakha 95 | 4.78stu±0.64 | 4.72st±0.75 | 4.63s→v±0.60 | 4.58tuv±0.73 | 4.21t±0.50 | 4.13tu±0.65 |
|  | Misr 3 | 4.82rst±0.65 | 4.77s±0.77 | 4.68q→u±0.61 | 4.63stu±0.74 | 4.44qrs±0.55 | 4.38qrs±0.70 |
|  | Gemmeiza-9 | 5.01m→r±0.69 | 4.97n→r±0.80 | 5.15lmn±0.73 | 5.11mn±0.82 | 4.97h→k±0.68 | 4.93h→k±0.79 |
|  | Giza-168 | 5.24jkl±0.76 | 5.22jkl±0.84 | 5.08mno±0.71 | 5.05mno±0.81 | 4.75l→p±0.63 | 4.70m→p±0.75 |
|  | Sids-14 | 5.53ghi±0.83 | 5.51hi±0.90 | 5.38h→k±0.79 | 5.36h→k±0.87 | 5.27c→g±0.76 | 5.25d→g±0.85 |
|  | SOKOLL | 5.66d→h±0.87 | 5.65fgh±0.92 | 5.50d→i±0.83 | 5.49f→i±0.90 | 5.33c→f±0.78 | 5.30c→f±0.86 |
|  | 18 SAWYT 19/20 | 5.81a→f±0.91 | 5.80a→f±0.96 | 5.64a→f±0.85 | 5.63b→f±0.92 | 4.89i→o±0.66 | 4.84j→o±0.77 |
| SiNPs_100_ | Giza 171 | 4.64tuv±0.60 | 4.59tuv±0.73 | 4.53uv±0.57 | 4.47uvw±0.71 | 4.23t±0.49 | 4.16tu±0.66 |
|  | Sakha 95 | 4.98n→s±0.69 | 4.50uv±0.71 | 4.78p→t±0.63 | 4.73q→t±0.76 | 4.26st±0.50 | 4.19tu±0.66 |
|  | Misr 3 | 5.09k→p±0.72 | 5.06l→p±0.82 | 4.85pqr±0.65 | 4.80pqr±0.76 | 4.51qr±0.57 | 4.45qr±0.71 |
|  | Gemmeiza-9 | 5.14j→o±0.73 | 5.11k→o±0.82 | 5.32i→l±0.78 | 5.30jkl±0.86 | 5.03hij±0.70 | 5.00hij±0.80 |
|  | Giza-168 | 5.28jk±0.77 | 5.25jk±0.85 | 5.18klm±0.74 | 5.15lm±0.83 | 4.89i→n±0.66 | 4.85j→n±0.78 |
|  | Sids-14 | 5.73b→g±0.88 | 5.73d→g±0.94 | 5.55d→h±0.84 | 5.53d→h±0.90 | 5.34b→e±0.78 | 5.32cde±0.86 |
|  | SOKOLL | 5.83a→e±0.91 | 5.83a→e±0.96 | 5.68a→e±0.87 | 5.67a→e±0.93 | 5.47bc±0.81 | 5.45bc±0.89 |
|  | 18 SAWYT 19/20 | 5.90ab±0.93 | 5.90abc±0.97 | 5.68a→d±0.87 | 5.68a→d±0.93 | 4.93h→l±0.68 | 4.89i→l±0.79 |
| SiNPs_200_ | Giza 171 | 4.71tuv±0.62 | 4.67stu±0.75 | 4.83p→s±0.65 | 4.78p→s±0.77 | 5.53b±0.83 | 5.52b±0.90 |
|  | Sakha 95 | 5.06l→q±0.71 | 5.02m→q±0.81 | 4.86pq±0.66 | 4.81pq±0.77 | 4.32rst±0.52 | 4.25st±0.67 |
|  | Misr 3 | 5.17j→n±0.73 | 5.14j→n±0.83 | 4.97nop±0.68 | 4.92op±0.78 | 4.60pq±0.58 | 4.54pq±0.72 |
|  | Gemmeiza-9 | 5.21j→m±0.75 | 5.18j→m±0.84 | 5.62a→g±0.86 | 5.61c→g±0.92 | 5.10gh±0.72 | 5.07h±0.81 |
|  | Giza-168 | 5.34ij±0.78 | 5.31j±0.86 | 5.42g→j±0.80 | 5.40hij±0.88 | 5.06hi±0.70 | 5.03hi±0.80 |
|  | Sids-14 | 5.84a→d±0.91 | 5.84a→d±0.96 | 5.82a±0.90 | 5.82a±0.95 | 5.37bcd±0.79 | 5.35bcd±0.87 |
|  | SOKOLL | 5.90ab±0.93 | 5.91ab±0.98 | 5.78abc±0.90 | 5.77abc±0.95 | 6.21a±1.01 | 6.23a±1.04 |
|  | 18 SAWYT 19/20 | 5.93a±0.94 | 5.94a±0.98 | 5.79ab±0.91 | 5.79ab±0.96 | 4.91h→m±0.67 | 4.87i→m±0.78 |
| The data of three replicates ± SE (standard error) are shown.  Means followed by different letters under the same water regimes were significantly different according to Duncan’s Multiple Range Test (p≤ 0.05) | | | | | | | |
