# Supplementary material for: Improving drought tolerance in some wheat genotypes with foliar application of silicon nanoparticles in Al-Dawadmi, Saudi Arabia
Source: PeerJ. 2026 Feb 24;14:e20823. doi: 10.7717/peerj.20823 (PMC12947762; doi:10.7717/peerj.20823)
Supplement: Supplemental Information 15 — The data of three replicates ± SE (standard error) are shown. Means followed by different letters under the same water regimes were significantly different according to Duncan’s Multiple Range Test (p ≤ 0.05) [file peerj-14-20823-s015.docx]

Supplementary Table S14. No. of leaves per plant of eight wheat genotypes as affected by foliar application of silicon nanoparticles under well-watered, moderate and severe water stress conditions during winter seasons of 2022/2023 (1^st^) and 2023/2024 (2^nd^ )

| SiNPs | No. of leaves per plant | | | | | | |
| --- | --- | --- | --- | --- | --- | --- | --- |
|  | Genotypes | Well-watered | | Moderate | | Severe | |
|  |  | 1st | 2nd | 1st | 2nd | 1st | 2nd |
| SiNPs_0_ | Giza 171 | 7.92v±1.09 | 7.94v±1.55 | 7.68v±1.05 | 7.69w±1.51 | 6.65t±0.85 | 6.62u±1.31 |
|  | Sakha 95 | 8.66stu±1.27 | 8.70st±1.70 | 8.22s→v±1.16 | 8.25tuv±1.62 | 6.90t±0.90 | 6.87tu±1.36 |
|  | Misr 3 | 8.79rst±1.30 | 8.85s±1.75 | 8.36q→u±1.20 | 8.40stu±1.66 | 7.62qrs±1.04 | 7.63qrs±1.50 |
|  | Gemmeiza-9 | 9.38m→r±1.43 | 9.47n→r±1.87 | 9.82lmn±1.55 | 9.92mn±1.96 | 9.25h→k±1.40 | 9.34h→k±1.84 |
|  | Giza-168 | 10.12jkl±1.63 | 10.24jkl±2.02 | 9.61mno±1.49 | 9.71mno±1.92 | 8.59l→p±1.26 | 8.64m→p±1.71 |
|  | Sids-14 | 11.00ghi±1.84 | 11.15hi±2.20 | 10.55h→k±1.73 | 10.68h→k±2.11 | 10.20c→g±1.63 | 10.33d→g±2.04 |
|  | SOKOLL | 11.42d→h±1.95 | 11.60fgh±2.29 | 10.93d→i±1.83 | 11.08f→i±2.21 | 10.37c→f±1.70 | 10.49c→f±2.07 |
|  | 18 SAWYT 19/20 | 11.86a→f±2.08 | 12.06a→f±2.40 | 11.34a→f±1.91 | 11.52b→f±2.28 | 9.00i→o±1.34 | 9.07j→o±1.77 |
| SiNPs_100_ | Giza 171 | 8.24tuv±1.16 | 8.28tuv±1.64 | 7.88uv±1.08 | 7.91uvw±1.57 | 6.95t±0.89 | 6.95tu±1.37 |
|  | Sakha 95 | 9.28n→s±1.41 | 8.00uv±1.57 | 8.67p→t±1.26 | 8.73q→t±1.73 | 7.04st±0.92 | 7.03tu±1.39 |
|  | Misr 3 | 9.64k→p±1.50 | 9.75l→p±1.92 | 8.89pqr±1.32 | 8.95pqr±1.75 | 7.82qr±1.08 | 7.84qr±1.54 |
|  | Gemmeiza-9 | 9.80j→o±1.54 | 9.89k→o±1.93 | 10.35i→l±1.67 | 10.49jkl±2.07 | 9.46hij±1.46 | 9.55hij±1.89 |
|  | Giza-168 | 10.22jk±1.65 | 10.35jk±2.04 | 9.93klm±1.57 | 10.04lm±1.98 | 9.02i→n±1.34 | 9.10j→n±1.80 |
|  | Sids-14 | 11.62b→g±1.99 | 11.82d→g±2.36 | 11.07d→h±1.86 | 11.23d→h±2.22 | 10.42b→e±1.69 | 10.57cde±2.09 |
|  | SOKOLL | 11.95a→e±2.08 | 12.15a→e±2.42 | 11.46a→e±1.95 | 11.64a→e±2.30 | 10.81bc±1.79 | 10.96bc±2.18 |
|  | 18 SAWYT 19/20 | 12.17ab±2.14 | 12.37abc±2.45 | 11.48a→d±1.96 | 11.66a→d±2.33 | 9.14h→l±1.38 | 9.22i→l±1.82 |
| SiNPs_200_ | Giza 171 | 8.47tuv±1.22 | 8.52stu±1.69 | 8.82p→s±1.31 | 8.89p→s±1.76 | 11.02b±1.83 | 11.19b±2.21 |
|  | Sakha 95 | 9.53l→q±1.48 | 9.63m→q±1.90 | 8.91pq±1.34 | 8.97pq±1.77 | 7.24rst±0.95 | 7.24st±1.42 |
|  | Misr 3 | 9.88j→n±1.54 | 10.00j→n±1.97 | 9.25nop±1.40 | 9.32op±1.82 | 8.09pq±1.12 | 8.13pq±1.59 |
|  | Gemmeiza-9 | 10.00j→m±1.58 | 10.12j→m±2.00 | 11.28a→g±1.92 | 11.45c→g±2.28 | 9.68gh±1.50 | 9.77h±1.91 |
|  | Giza-168 | 10.40ij±1.70 | 10.53j±2.08 | 10.68g→j±1.76 | 10.82hij±2.14 | 9.56hi±1.46 | 9.65hi±1.89 |
|  | Sids-14 | 11.98a→d±2.08 | 12.19a→d±2.43 | 11.92a±2.07 | 12.12a±2.40 | 10.52bcd±1.71 | 10.66bcd±2.10 |
|  | SOKOLL | 12.17ab±2.14 | 12.39ab±2.47 | 11.78abc±2.04 | 11.97abc±2.37 | 13.12a±2.38 | 13.38a±2.67 |
|  | 18 SAWYT 19/20 | 12.27a±2.16 | 12.48a±2.49 | 11.83ab±2.07 | 12.02ab±2.40 | 9.08h→m±1.37 | 9.15i→m±1.79 |
| The data of three replicates ± SE (standard error) are shown.  Means followed by different letters under the same water regimes were significantly different according to Duncan’s Multiple Range Test (p≤ 0.05) | | | | | | | |
