# Supplementary material for: Improving drought tolerance in some wheat genotypes with foliar application of silicon nanoparticles in Al-Dawadmi, Saudi Arabia
Source: PeerJ. 2026 Feb 24;14:e20823. doi: 10.7717/peerj.20823 (PMC12947762; doi:10.7717/peerj.20823)
Supplement: Supplemental Information 16 — The data of three replicates ± SE (standard error) are shown. Means followed by different letters under the same water regimes were significantly different according to Duncan’s Multiple Range Test (p ≤ 0.05) [file peerj-14-20823-s016.docx]

Supplementary Table S15. Shoot DW per plant of eight wheat genotypes as affected by foliar application of silicon nanoparticles under well-watered, moderate and severe water stress conditions during winter seasons of 2022/2023 (1^st^) and 2023/2024 (2^nd^ )

| SiNPs | Shoot DW per plant | | | | | | |
| --- | --- | --- | --- | --- | --- | --- | --- |
|  | Genotypes | Well-watered | | Moderate | | Severe | |
|  |  | 1st | 2nd | 1st | 2nd | 1st | 2nd |
| SiNPs_0_ | Giza 171 | 6.42v±0.82 | 5.96v±1.01 | 6.29v±0.80 | 5.82w±0.99 | 5.74t±0.67 | 5.25u±0.90 |
|  | Sakha 95 | 6.82stu±0.93 | 6.37st±1.09 | 6.58s→v±0.86 | 6.13tuv±1.05 | 5.87t±0.70 | 5.39tu±0.92 |
|  | Misr 3 | 6.89rst±0.94 | 6.45s±1.11 | 6.66q→u±0.89 | 6.21stu±1.07 | 6.26qrs±0.79 | 5.80qrs±0.99 |
|  | Gemmeiza-9 | 7.20m→r±1.02 | 6.78n→r±1.17 | 7.44lmn±1.09 | 7.02mn±1.22 | 7.13h→k±1.00 | 6.71h→k±1.16 |
|  | Giza-168 | 7.60jkl±1.13 | 7.19jkl±1.25 | 7.32mno±1.05 | 6.91mno±1.20 | 6.78l→p±0.92 | 6.34m→p±1.09 |
|  | Sids-14 | 8.07ghi±1.25 | 7.68hi±1.34 | 7.83h→k±1.19 | 7.43h→k±1.30 | 7.64c→g±1.13 | 7.24d→g±1.26 |
|  | SOKOLL | 8.30d→h±1.30 | 7.92fgh±1.39 | 8.03d→i±1.24 | 7.65f→i±1.35 | 7.73c→f±1.17 | 7.33c→f±1.27 |
|  | 18 SAWYT 19/20 | 8.54a→f±1.38 | 8.17a→f±1.45 | 8.25a→f±1.29 | 7.88b→f±1.38 | 7.00i→o±0.97 | 6.57j→o±1.12 |
| SiNPs_100_ | Giza 171 | 6.59tuv±0.87 | 6.14tuv±1.06 | 6.40uv±0.82 | 5.95uvw±1.02 | 5.90t±0.70 | 5.43tu±0.92 |
|  | Sakha 95 | 7.15n→s±1.01 | 5.99uv±1.02 | 6.82p→t±0.92 | 6.39q→t±1.10 | 5.95st±0.71 | 5.47tu±0.93 |
|  | Misr 3 | 7.34k→p±1.06 | 6.93l→p±1.20 | 6.94pqr±0.95 | 6.50pqr±1.11 | 6.37qr±0.81 | 5.91qr±1.01 |
|  | Gemmeiza-9 | 7.43j→o±1.08 | 7.01k→o±1.20 | 7.73i→l±1.15 | 7.33jkl±1.27 | 7.25hij±1.04 | 6.82hij±1.18 |
|  | Giza-168 | 7.66jk±1.14 | 7.26jk±1.26 | 7.50klm±1.10 | 7.09lm±1.23 | 7.01i→n±0.97 | 6.58j→n±1.14 |
|  | Sids-14 | 8.41b→g±1.33 | 8.04d→g±1.42 | 8.11d→h±1.25 | 7.72d→h±1.35 | 7.76b→e±1.16 | 7.37cde±1.28 |
|  | SOKOLL | 8.58a→e±1.38 | 8.22a→e±1.46 | 8.32a→e±1.31 | 7.94a→e±1.39 | 7.97bc±1.22 | 7.58bc±1.33 |
|  | 18 SAWYT 19/20 | 8.70ab±1.41 | 8.34abc±1.47 | 8.33a→d±1.31 | 7.96a→d±1.41 | 7.08h→l±0.99 | 6.65i→l±1.15 |
| SiNPs_200_ | Giza 171 | 6.71tuv±0.90 | 6.27stu±1.08 | 6.90p→s±0.95 | 6.47p→s±1.12 | 8.08b±1.24 | 7.70b±1.35 |
|  | Sakha 95 | 7.29l→q±1.04 | 6.87m→q±1.19 | 6.95pq±0.97 | 6.51pq±1.12 | 6.06rst±0.73 | 5.58st±0.95 |
|  | Misr 3 | 7.47j→n±1.08 | 7.06j→n±1.23 | 7.13nop±1.00 | 6.70op±1.15 | 6.51pq±0.84 | 6.07pq±1.03 |
|  | Gemmeiza-9 | 7.53j→m±1.11 | 7.13j→m±1.24 | 8.22a→g±1.29 | 7.84c→g±1.38 | 7.36gh±1.06 | 6.94h±1.19 |
|  | Giza-168 | 7.75ij±1.17 | 7.35j±1.28 | 7.90g→j±1.20 | 7.50hij±1.31 | 7.30hi±1.04 | 6.88hi±1.18 |
|  | Sids-14 | 8.60a→d±1.38 | 8.24a→d±1.46 | 8.57a±1.37 | 8.20a±1.44 | 7.81bcd±1.17 | 7.42bcd±1.29 |
|  | SOKOLL | 8.70ab±1.41 | 8.35ab±1.48 | 8.49abc±1.36 | 8.12abc±1.43 | 9.21a±1.54 | 8.88a±1.59 |
|  | 18 SAWYT 19/20 | 8.75a±1.42 | 8.40a±1.49 | 8.52ab±1.37 | 8.15ab±1.44 | 7.04h→m±0.98 | 6.61i→m±1.13 |
| The data of three replicates ± SE (standard error) are shown.  Means followed by different letters under the same water regimes were significantly different according to Duncan’s Multiple Range Test (p≤ 0.05) | | | | | | | |
