# Supplementary material for: Improving drought tolerance in some wheat genotypes with foliar application of silicon nanoparticles in Al-Dawadmi, Saudi Arabia
Source: PeerJ. 2026 Feb 24;14:e20823. doi: 10.7717/peerj.20823 (PMC12947762; doi:10.7717/peerj.20823)
Supplement: Supplemental Information 17 — The data of three replicates ± SE (standard error) are shown. Means followed by different letters under the same water regimes were significantly different according to Duncan’s Multiple Range Test (p ≤ 0.05) [file peerj-14-20823-s017.docx]

Supplementary Table S16. Spike length of eight wheat genotypes as affected by foliar application of silicon nanoparticles under well-watered, moderate and severe water stress conditions during winter seasons of 2022/2023 (1^st^) and 2023/2024 (2^nd^ )

| SiNPs | Spike length | | | | | | |
| --- | --- | --- | --- | --- | --- | --- | --- |
|  | Genotypes | Well-watered | | Moderate | | Severe | |
|  |  | 1st | 2nd | 1st | 2nd | 1st | 2nd |
| SiNPs_0_ | Giza 171 | 12.43v±1.57 | 9.56v±1.64 | 12.22v±1.53 | 9.33w±1.61 | 11.30t±1.30 | 8.38u±1.45 |
|  | Sakha 95 | 13.09stu±1.75 | 10.24st±1.77 | 12.69s→v±1.64 | 9.84tuv±1.70 | 11.52t±1.36 | 8.61tu±1.48 |
|  | Misr 3 | 13.20rst±1.78 | 10.37s±1.81 | 12.82q→u±1.68 | 9.97stu±1.74 | 12.17qrs±1.52 | 9.29qrs±1.60 |
|  | Gemmeiza-9 | 13.73m→r±1.90 | 10.92n→r±1.91 | 14.12lmn±2.02 | 11.32mn±1.98 | 13.61h→k±1.88 | 10.80h→k±1.89 |
|  | Giza-168 | 14.39jkl±2.08 | 11.60jkl±2.04 | 13.93mno±1.96 | 11.13mno±1.95 | 13.02l→p±1.74 | 10.18m→p±1.78 |
|  | Sids-14 | 15.17ghi±2.28 | 12.41hi±2.19 | 14.77h→k±2.18 | 12.00h→k±2.12 | 14.46c→g±2.09 | 11.68d→g±2.05 |
|  | SOKOLL | 15.55d→h±2.38 | 12.81fgh±2.27 | 15.10d→i±2.27 | 12.35f→i±2.20 | 14.61c→f±2.15 | 11.82c→f±2.08 |
|  | 18 SAWYT 19/20 | 15.94a→f±2.50 | 13.22a→f±2.37 | 15.47a→f±2.35 | 12.74b→f±2.26 | 13.39i→o±1.81 | 10.57j→o±1.83 |
| SiNPs_100_ | Giza 171 | 12.72tuv±1.64 | 9.86tuv±1.72 | 12.40uv±1.56 | 9.53uvw±1.66 | 11.57t±1.35 | 8.68tu±1.50 |
|  | Sakha 95 | 13.65n→s±1.88 | 9.61uv±1.66 | 13.10p→t±1.74 | 10.26q→t±1.79 | 11.65st±1.38 | 8.75tu±1.51 |
|  | Misr 3 | 13.96k→p±1.96 | 11.17l→p±1.96 | 13.30pqr±1.79 | 10.46pqr±1.81 | 12.35qr±1.55 | 9.47qr±1.63 |
|  | Gemmeiza-9 | 14.10j→o±2.01 | 11.29k→o±1.96 | 14.60i→l±2.13 | 11.82jkl±2.08 | 13.81hij±1.93 | 10.99hij±1.92 |
|  | Giza-168 | 14.48jk±2.10 | 11.71jk±2.06 | 14.22klm±2.03 | 11.42lm±2.01 | 13.41i→n±1.82 | 10.59j→n±1.85 |
|  | Sids-14 | 15.73b→g±2.42 | 13.01d→g±2.33 | 15.23d→h±2.30 | 12.48d→h±2.21 | 14.66b→e±2.14 | 11.89cde±2.10 |
|  | SOKOLL | 16.01a→e±2.50 | 13.30a→e±2.38 | 15.58a→e±2.39 | 12.84a→e±2.28 | 15.00bc±2.23 | 12.25bc±2.18 |
|  | 18 SAWYT 19/20 | 16.21ab±2.56 | 13.50abc±2.41 | 15.60a→d±2.40 | 12.87a→d±2.30 | 13.52h→l±1.86 | 10.70i→l±1.87 |
| SiNPs_200_ | Giza 171 | 12.92tuv±1.70 | 10.07stu±1.76 | 13.23p→s±1.78 | 10.40p→s±1.82 | 15.19b±2.27 | 12.45b±2.20 |
|  | Sakha 95 | 13.87l→q±1.95 | 11.06m→q±1.94 | 13.31pq±1.81 | 10.47pq±1.83 | 11.83rst±1.42 | 8.93st±1.54 |
|  | Misr 3 | 14.17j→n±2.01 | 11.39j→n±2.00 | 13.61nop±1.88 | 10.79op±1.87 | 12.59pq±1.60 | 9.73pq±1.68 |
|  | Gemmeiza-9 | 14.28j→m±2.05 | 11.49j→m±2.02 | 15.42a→g±2.36 | 12.68c→g±2.26 | 13.99gh±1.97 | 11.19h±1.94 |
|  | Giza-168 | 14.64ij±2.16 | 11.86j±2.09 | 14.88g→j±2.21 | 12.12hij±2.14 | 13.89hi±1.93 | 11.08hi±1.92 |
|  | Sids-14 | 16.04a→d±2.50 | 13.34a→d±2.39 | 15.99a±2.49 | 13.28a±2.36 | 14.74bcd±2.16 | 11.98bcd±2.11 |
|  | SOKOLL | 16.21ab±2.56 | 13.52ab±2.43 | 15.86abc±2.46 | 13.14abc±2.33 | 17.06a±2.77 | 14.40a±2.60 |
|  | 18 SAWYT 19/20 | 16.30a±2.58 | 13.60a±2.44 | 15.91ab±2.49 | 13.19ab±2.36 | 13.47h→m±1.84 | 10.64i→m±1.84 |
| The data of three replicates ± SE (standard error) are shown.  Means followed by different letters under the same water regimes were significantly different according to Duncan’s Multiple Range Test (p≤ 0.05) | | | | | | | |
