# Supplementary material for: Improving drought tolerance in some wheat genotypes with foliar application of silicon nanoparticles in Al-Dawadmi, Saudi Arabia
Source: PeerJ. 2026 Feb 24;14:e20823. doi: 10.7717/peerj.20823 (PMC12947762; doi:10.7717/peerj.20823)
Supplement: Supplemental Information 18 — The data of three replicates ± SE (standard error) are shown. Means followed by different letters under the same water regimes were significantly different according to Duncan’s Multiple Range Test (p ≤ 0.05) [file peerj-14-20823-s018.docx]

Supplementary Table S17. No. of spikes per plant of eight wheat genotypes as affected by foliar application of silicon nanoparticles under well-watered, moderate and severe water stress conditions during winter seasons of 2022/2023 (1^st^) and 2023/2024 (2^nd^ )

| SiNPs | No. of spikes per plant | | | | | | |
| --- | --- | --- | --- | --- | --- | --- | --- |
|  | Genotypes | Well-watered | | Moderate | | Severe | |
|  |  | 1st | 2nd | 1st | 2nd | 1st | 2nd |
| SiNPs_0_ | Giza 171 | 12.11v±1.56 | 10.89v±1.90 | 11.86v±1.51 | 10.63w±1.86 | 10.77t±1.25 | 9.50u±1.67 |
|  | Sakha 95 | 12.89stu±1.77 | 11.70st±2.05 | 12.43s→v±1.64 | 11.22tuv±1.97 | 11.04t±1.32 | 9.76tu±1.71 |
|  | Misr 3 | 13.03rst±1.80 | 11.85s±2.10 | 12.58q→u±1.69 | 11.38stu±2.01 | 11.80qrs±1.50 | 10.57qrs±1.85 |
|  | Gemmeiza-9 | 13.66m→r±1.94 | 12.51n→r±2.22 | 14.12lmn±2.08 | 12.98mn±2.31 | 13.52h→k±1.91 | 12.37h→k±2.20 |
|  | Giza-168 | 14.44jkl±2.16 | 13.32jkl±2.38 | 13.90mno±2.01 | 12.76mno±2.27 | 12.82l→p±1.75 | 11.63m→p±2.06 |
|  | Sids-14 | 15.36ghi±2.39 | 14.28hi±2.56 | 14.89h→k±2.27 | 13.79h→k±2.47 | 14.52c→g±2.16 | 13.42d→g±2.39 |
|  | SOKOLL | 15.81d→h±2.51 | 14.75fgh±2.65 | 15.29d→i±2.38 | 14.21f→i±2.57 | 14.70c→f±2.23 | 13.58c→f±2.43 |
|  | 18 SAWYT 19/20 | 16.28a→f±2.64 | 15.24a→f±2.77 | 15.72a→f±2.47 | 14.67b→f±2.64 | 13.25i→o±1.84 | 12.09j→o±2.12 |
| SiNPs_100_ | Giza 171 | 12.45tuv±1.64 | 11.26tuv±1.99 | 12.08uv±1.55 | 10.87uvw±1.92 | 11.10t±1.31 | 9.84tu±1.72 |
|  | Sakha 95 | 13.56n→s±1.92 | 10.96uv±1.92 | 12.90p→t±1.75 | 11.73q→t±2.08 | 11.19st±1.35 | 9.93tu±1.74 |
|  | Misr 3 | 13.93k→p±2.02 | 12.80l→p±2.28 | 13.14pqr±1.82 | 11.96pqr±2.10 | 12.01qr±1.54 | 10.79qr±1.89 |
|  | Gemmeiza-9 | 14.10j→o±2.07 | 12.95k→o±2.29 | 14.69i→l±2.21 | 13.58jkl±2.43 | 13.75hij±1.98 | 12.59hij±2.24 |
|  | Giza-168 | 14.55jk±2.18 | 13.44jk±2.40 | 14.23klm±2.10 | 13.11lm±2.34 | 13.28i→n±1.85 | 12.12j→n±2.15 |
|  | Sids-14 | 16.03b→g±2.55 | 14.99d→g±2.72 | 15.44d→h±2.41 | 14.36d→h±2.58 | 14.76b→e±2.22 | 13.67cde±2.44 |
|  | SOKOLL | 16.37a→e±2.64 | 15.34a→e±2.79 | 15.85a→e±2.51 | 14.79a→e±2.66 | 15.16bc±2.33 | 14.09bc±2.54 |
|  | 18 SAWYT 19/20 | 16.60ab±2.71 | 15.58abc±2.81 | 15.88a→d±2.52 | 14.83a→d±2.69 | 13.41h→l±1.89 | 12.24i→l±2.17 |
| SiNPs_200_ | Giza 171 | 12.69tuv±1.71 | 11.51stu±2.04 | 13.07p→s±1.80 | 11.90p→s±2.11 | 15.39b±2.38 | 14.32b±2.57 |
|  | Sakha 95 | 13.82l→q±2.00 | 12.68m→q±2.25 | 13.16pq±1.84 | 11.98pq±2.13 | 11.40rst±1.39 | 10.15st±1.77 |
|  | Misr 3 | 14.18j→n±2.07 | 13.07j→n±2.33 | 13.52nop±1.91 | 12.35op±2.17 | 12.30pq±1.59 | 11.10pq±1.94 |
|  | Gemmeiza-9 | 14.31j→m±2.11 | 13.19j→m±2.35 | 15.66a→g±2.48 | 14.60c→g±2.64 | 13.97gh±2.02 | 12.83h±2.26 |
|  | Giza-168 | 14.74ij±2.24 | 13.62j±2.43 | 15.02g→j±2.30 | 13.93hij±2.49 | 13.84hi±1.98 | 12.70hi±2.24 |
|  | Sids-14 | 16.40a→d±2.65 | 15.38a→d±2.80 | 16.34a±2.63 | 15.31a±2.76 | 14.86bcd±2.25 | 13.76bcd±2.46 |
|  | SOKOLL | 16.60ab±2.71 | 15.59ab±2.84 | 16.19abc±2.60 | 15.14abc±2.73 | 17.61a±2.97 | 16.64a±3.04 |
|  | 18 SAWYT 19/20 | 16.70a±2.74 | 15.69a±2.86 | 16.24ab±2.64 | 15.20ab±2.76 | 13.34h→m±1.88 | 12.17i→m±2.14 |
| The data of three replicates ± SE (standard error) are shown.  Means followed by different letters under the same water regimes were significantly different according to Duncan’s Multiple Range Test (p≤ 0.05) | | | | | | | |
