# Supplementary material for: Improving drought tolerance in some wheat genotypes with foliar application of silicon nanoparticles in Al-Dawadmi, Saudi Arabia
Source: PeerJ. 2026 Feb 24;14:e20823. doi: 10.7717/peerj.20823 (PMC12947762; doi:10.7717/peerj.20823)
Supplement: Supplemental Information 19 — The data of three replicates ± SE (standard error) are shown. Means followed by different letters under the same water regimes were significantly different according to Duncan’s Multiple Range Test (p ≤ 0.05) [file peerj-14-20823-s019.docx]

Supplementary Table S18. No. of grains per spike of eight wheat genotypes as affected by foliar application of silicon nanoparticles under well-watered, moderate and severe water stress conditions during winter seasons of 2022/2023 (1^st^) and 2023/2024 (2^nd^ )

| SiNPs | No. of grains per spike | | | | | | |
| --- | --- | --- | --- | --- | --- | --- | --- |
|  | Genotypes | Well-watered | | Moderate | | Severe | |
|  |  | 1st | 2nd | 1st | 2nd | 1st | 2nd |
| SiNPs_0_ | Giza 171 | 35.67v±4.66 | 32.58v±5.91 | 34.85v±4.51 | 31.72w±5.76 | 31.27t±3.70 | 28.01u±5.12 |
|  | Sakha 95 | 38.23stu±5.33 | 35.24st±6.41 | 36.70s→v±4.91 | 33.68tuv±6.12 | 32.14t±3.90 | 28.88tu±5.26 |
|  | Misr 3 | 38.68rst±5.42 | 35.75s±6.57 | 37.20q→u±5.08 | 34.19stu±6.28 | 34.64qrs±4.47 | 31.53qrs±5.74 |
|  | Gemmeiza-9 | 40.73m→r±5.90 | 37.90n→r±6.97 | 42.26lmn±6.33 | 39.45mn±7.27 | 40.28h→k±5.80 | 37.44h→k±6.89 |
|  | Giza-168 | 43.29jkl±6.60 | 40.55jkl±7.49 | 41.51mno±6.11 | 38.72mno±7.13 | 37.98l→p±5.29 | 35.01m→p±6.44 |
|  | Sids-14 | 46.33ghi±7.36 | 43.71hi±8.10 | 44.77h→k±6.96 | 42.10h→k±7.79 | 43.57c→g±6.60 | 40.87d→g±7.54 |
|  | SOKOLL | 47.81d→h±7.73 | 45.26fgh±8.41 | 46.09d→i±7.31 | 43.49f→i±8.12 | 44.16c→f±6.85 | 41.42c→f±7.65 |
|  | 18 SAWYT 19/20 | 49.34a→f±8.18 | 46.87a→f±8.79 | 47.52a→f±7.60 | 44.99b→f±8.35 | 39.41i→o±5.56 | 36.52j→o±6.65 |
| SiNPs_100_ | Giza 171 | 36.78tuv±4.93 | 33.78tuv±6.20 | 35.55uv±4.64 | 32.50uvw±5.97 | 32.33t±3.88 | 29.15tu±5.31 |
|  | Sakha 95 | 40.40n→s±5.83 | 32.81uv±5.97 | 38.26p→t±5.28 | 35.34q→t±6.49 | 32.63st±3.99 | 29.43tu±5.36 |
|  | Misr 3 | 41.64k→p±6.14 | 38.86l→p±7.16 | 39.04pqr±5.49 | 36.11pqr±6.57 | 35.34qr±4.60 | 32.26qr±5.86 |
|  | Gemmeiza-9 | 42.18j→o±6.31 | 39.35k→o±7.19 | 44.11i→l±6.77 | 41.42jkl±7.65 | 41.02hij±6.02 | 38.17hij±7.03 |
|  | Giza-168 | 43.66jk±6.67 | 40.96jk±7.57 | 42.63klm±6.40 | 39.86lm±7.35 | 39.50i→n±5.59 | 36.62j→n±6.73 |
|  | Sids-14 | 48.51b→g±7.88 | 46.05d→g±8.63 | 46.58d→h±7.41 | 43.98d→h±8.15 | 44.35b→e±6.81 | 41.69cde±7.71 |
|  | SOKOLL | 49.62a→e±8.18 | 47.19a→e±8.85 | 47.93a→e±7.75 | 45.40a→e±8.43 | 45.67bc±7.17 | 43.07bc±8.04 |
|  | 18 SAWYT 19/20 | 50.40ab±8.40 | 47.96abc±8.94 | 48.02a→d±7.79 | 45.50a→d±8.52 | 39.91h→l±5.73 | 37.03i→l±6.81 |
| SiNPs_200_ | Giza 171 | 37.56tuv±5.14 | 34.60stu±6.36 | 38.80p→s±5.45 | 35.88p→s±6.60 | 46.40b±7.31 | 43.84b±8.13 |
|  | Sakha 95 | 41.27l→q±6.07 | 38.45m→q±7.08 | 39.09pq±5.57 | 36.16pq±6.65 | 33.32rst±4.11 | 30.16st±5.48 |
|  | Misr 3 | 42.46j→n±6.30 | 39.73j→n±7.32 | 40.28nop±5.80 | 37.39op±6.81 | 36.28pq±4.77 | 33.27pq±6.04 |
|  | Gemmeiza-9 | 42.87j→m±6.45 | 40.14j→m±7.41 | 47.32a→g±7.64 | 44.77c→g±8.37 | 41.76gh±6.16 | 38.94h±7.11 |
|  | Giza-168 | 44.28ij±6.87 | 41.56j±7.68 | 45.22g→j±7.06 | 42.56hij±7.87 | 41.34hi±6.01 | 38.53hi±7.03 |
|  | Sids-14 | 49.74a→d±8.21 | 47.33a→d±8.88 | 49.54a±8.15 | 47.09a±8.77 | 44.68bcd±6.89 | 42.01bcd±7.77 |
|  | SOKOLL | 50.40ab±8.40 | 48.01ab±9.02 | 49.05abc±8.05 | 46.54abc±8.66 | 53.69a±9.23 | 51.44a±9.71 |
|  | 18 SAWYT 19/20 | 50.73a±8.48 | 48.33a±9.08 | 49.22ab±8.16 | 46.73ab±8.76 | 39.71h→m±5.68 | 36.79i→m±6.70 |
| The data of three replicates ± SE (standard error) are shown.  Means followed by different letters under the same water regimes were significantly different according to Duncan’s Multiple Range Test (p≤ 0.05) | | | | | | | |
