# Supplementary material for: Improving drought tolerance in some wheat genotypes with foliar application of silicon nanoparticles in Al-Dawadmi, Saudi Arabia
Source: PeerJ. 2026 Feb 24;14:e20823. doi: 10.7717/peerj.20823 (PMC12947762; doi:10.7717/peerj.20823)
Supplement: Supplemental Information 20 — The data of three replicates ± SE (standard error) are shown. Means followed by different letters under the same water regimes were significantly different according to Duncan’s Multiple Range Test (p ≤ 0.05) [file peerj-14-20823-s020.docx]

Supplementary Table S19. Straw yield per hectare of eight wheat genotypes as affected by foliar application of silicon nanoparticles under well-watered, moderate and severe water stress conditions during winter seasons of 2022/2023 (1^st^) and 2023/2024 (2^nd^ )

| SiNPs | Straw yield per hectare | | | | | | |
| --- | --- | --- | --- | --- | --- | --- | --- |
|  | Genotypes | Well-watered | | Moderate | | Severe | |
|  |  | 1st | 2nd | 1st | 2nd | 1st | 2nd |
| SiNPs_0_ | Giza 171 | 9.39v±1.18 | 9.31v±1.47 | 9.23v±1.15 | 9.15w±1.45 | 8.54t±0.98 | 8.43u±1.34 |
|  | Sakha 95 | 9.88stu±1.32 | 9.82st±1.56 | 9.59s→v±1.24 | 9.52tuv±1.51 | 8.71t±1.03 | 8.60tu±1.36 |
|  | Misr 3 | 9.97rst±1.34 | 9.92s±1.59 | 9.68q→u±1.27 | 9.62stu±1.54 | 9.19qrs±1.14 | 9.11qrs±1.45 |
|  | Gemmeiza-9 | 10.36m→r±1.43 | 10.34n→r±1.66 | 10.66lmn±1.52 | 10.63mn±1.71 | 10.27h→k±1.41 | 10.25h→k±1.65 |
|  | Giza-168 | 10.85jkl±1.57 | 10.85jkl±1.75 | 10.51mno±1.48 | 10.49mno±1.69 | 9.83l→p±1.31 | 9.78m→p±1.57 |
|  | Sids-14 | 11.44ghi±1.72 | 11.45hi±1.86 | 11.14h→k±1.64 | 11.14h→k±1.81 | 10.91c→g±1.57 | 10.91d→g±1.76 |
|  | SOKOLL | 11.72d→h±1.79 | 11.75fgh±1.92 | 11.39d→i±1.71 | 11.41f→i±1.87 | 11.02c→f±1.62 | 11.01c→f±1.78 |
|  | 18 SAWYT 19/20 | 12.02a→f±1.88 | 12.06a→f±1.99 | 11.67a→f±1.77 | 11.70b→f±1.91 | 10.11i→o±1.37 | 10.07j→o±1.60 |
| SiNPs_100_ | Giza 171 | 9.60tuv±1.24 | 9.54tuv±1.52 | 9.36uv±1.18 | 9.30uvw±1.48 | 8.74t±1.02 | 8.65tu±1.37 |
|  | Sakha 95 | 10.30n→s±1.42 | 9.36uv±1.48 | 9.89p→t±1.31 | 9.84q→t±1.58 | 8.80st±1.04 | 8.71tu±1.38 |
|  | Misr 3 | 10.54k→p±1.48 | 10.52l→p±1.70 | 10.04pqr±1.35 | 9.99pqr±1.59 | 9.32qr±1.17 | 9.25qr±1.47 |
|  | Gemmeiza-9 | 10.64j→o±1.51 | 10.61k→o±1.70 | 11.01i→l±1.61 | 11.01jkl±1.78 | 10.42hij±1.46 | 10.39hij±1.67 |
|  | Giza-168 | 10.92jk±1.58 | 10.92jk±1.77 | 10.73klm±1.53 | 10.71lm±1.73 | 10.12i→n±1.37 | 10.09j→n±1.62 |
|  | Sids-14 | 11.86b→g±1.82 | 11.90d→g±1.96 | 11.49d→h±1.73 | 11.51d→h±1.87 | 11.06b→e±1.61 | 11.07cde±1.79 |
|  | SOKOLL | 12.07a→e±1.88 | 12.12a→e±2.00 | 11.75a→e±1.80 | 11.78a→e±1.92 | 11.31bc±1.68 | 11.33bc±1.85 |
|  | 18 SAWYT 19/20 | 12.22ab±1.92 | 12.27abc±2.01 | 11.76a→d±1.80 | 11.80a→d±1.94 | 10.20h→l±1.40 | 10.17i→l±1.63 |
| SiNPs_200_ | Giza 171 | 9.75tuv±1.28 | 9.70stu±1.55 | 9.99p→s±1.34 | 9.95p→s±1.60 | 11.45b±1.71 | 11.48b±1.87 |
|  | Sakha 95 | 10.46l→q±1.47 | 10.44m→q±1.68 | 10.05pq±1.37 | 10.00pq±1.61 | 8.93rst±1.07 | 8.85st±1.40 |
|  | Misr 3 | 10.69j→n±1.51 | 10.69j→n±1.72 | 10.27nop±1.41 | 10.24op±1.63 | 9.50pq±1.21 | 9.44pq±1.50 |
|  | Gemmeiza-9 | 10.77j→m±1.54 | 10.77j→m±1.74 | 11.63a→g±1.78 | 11.66c→g±1.91 | 10.56gh±1.49 | 10.54h±1.68 |
|  | Giza-168 | 11.04ij±1.62 | 11.04j±1.79 | 11.22g→j±1.66 | 11.23hij±1.82 | 10.48hi±1.46 | 10.46hi±1.67 |
|  | Sids-14 | 12.10a→d±1.89 | 12.15a→d±2.00 | 12.06a±1.87 | 12.10a±1.98 | 11.12bcd±1.63 | 11.13bcd±1.80 |
|  | SOKOLL | 12.22ab±1.92 | 12.28ab±2.03 | 11.96abc±1.86 | 12.00abc±1.96 | 12.86a±2.09 | 12.94a±2.15 |
|  | 18 SAWYT 19/20 | 12.29a±1.94 | 12.34a±2.04 | 11.99ab±1.88 | 12.04ab±1.98 | 10.16h→m±1.39 | 10.12i→m±1.61 |
| The data of three replicates ± SD (standard deviation) are shown.  Means followed by different letters under the same water regimes were significantly different according to Duncan’s Multiple Range Test (p≤ 0.05) | | | | | | | |
