# Supplementary material for: Improving drought tolerance in some wheat genotypes with foliar application of silicon nanoparticles in Al-Dawadmi, Saudi Arabia
Source: PeerJ. 2026 Feb 24;14:e20823. doi: 10.7717/peerj.20823 (PMC12947762; doi:10.7717/peerj.20823)
Supplement: Supplemental Information 21 — The data of three replicates ± SE (standard error) are shown. Means followed by different letters under the same water regimes were significantly different according to Duncan’s Multiple Range Test (p ≤ 0.05) [file peerj-14-20823-s021.docx]

Supplementary Table S20. Grain yield per hectare of eight wheat genotypes as affected by foliar application of silicon nanoparticles under well-watered, moderate and severe water stress conditions during winter seasons of 2022/2023 (1^st^) and 2023/2024 (2^nd^ )

| SiNPs | Grain yield per hectare | | | | | | |
| --- | --- | --- | --- | --- | --- | --- | --- |
|  | Genotypes | Well-watered | | Moderate | | Severe | |
|  |  | 1st | 2nd | 1st | 2nd | 1st | 2nd |
| SiNPs_0_ | Giza 171 | 5.66v±0.74 | 4.99v±0.75 | 5.52v±0.72 | 4.92w±0.74 | 4.93t±0.59 | 4.62u±0.69 |
|  | Sakha 95 | 6.08stu±0.85 | 5.21st±0.78 | 5.83s→v±0.78 | 5.08tuv±0.76 | 5.08t±0.62 | 4.69tu±0.70 |
|  | Misr 3 | 6.15rst±0.87 | 5.25s±0.79 | 5.91q→u±0.81 | 5.12stu±0.77 | 5.49qrs±0.71 | 4.91qrs±0.74 |
|  | Gemmeiza-9 | 6.49m→r±0.94 | 5.43n→r±0.82 | 6.74lmn±1.02 | 5.55mn±0.84 | 6.41h→k±0.93 | 5.39h→k±0.82 |
|  | Giza-168 | 6.91jkl±1.06 | 5.64jkl±0.86 | 6.62mno±0.98 | 5.49mno±0.83 | 6.04l→p±0.85 | 5.19m→p±0.79 |
|  | Sids-14 | 7.41ghi±1.18 | 5.90hi±0.90 | 7.15h→k±1.12 | 5.77h→k±0.88 | 6.95c→g±1.06 | 5.67d→g±0.86 |
|  | SOKOLL | 7.65d→h±1.24 | 6.03fgh±0.93 | 7.37d→i±1.18 | 5.89f→i±0.91 | 7.05c→f±1.10 | 5.72c→f±0.87 |
|  | 18 SAWYT 19/20 | 7.90a→f±1.32 | 6.16a→f±0.96 | 7.60a→f±1.22 | 6.01b→f±0.92 | 6.27i→o±0.89 | 5.31j→o±0.80 |
| SiNPs_100_ | Giza 171 | 5.84tuv±0.79 | 5.09tuv±0.77 | 5.64uv±0.74 | 4.98uvw±0.75 | 5.11t±0.62 | 4.71tu±0.70 |
|  | Sakha 95 | 6.43n→s±0.93 | 5.01uv±0.75 | 6.08p→t±0.84 | 5.22q→t±0.79 | 5.16st±0.63 | 4.73tu±0.71 |
|  | Misr 3 | 6.64k→p±0.98 | 5.51l→p±0.84 | 6.21pqr±0.88 | 5.28pqr±0.79 | 5.60qr±0.73 | 4.97qr±0.74 |
|  | Gemmeiza-9 | 6.72j→o±1.01 | 5.55k→o±0.84 | 7.04i→l±1.09 | 5.72jkl±0.87 | 6.54hij±0.96 | 5.45hij±0.83 |
|  | Giza-168 | 6.97jk±1.07 | 5.68jk±0.87 | 6.80klm±1.03 | 5.59lm±0.85 | 6.28i→n±0.89 | 5.32j→n±0.80 |
|  | Sids-14 | 7.76b→g±1.27 | 6.10d→g±0.94 | 7.45d→h±1.19 | 5.93d→h±0.91 | 7.08b→e±1.09 | 5.74cde±0.88 |
|  | SOKOLL | 7.95a→e±1.32 | 6.19a→e±0.96 | 7.67a→e±1.25 | 6.04a→e±0.93 | 7.30bc±1.15 | 5.85bc±0.90 |
|  | 18 SAWYT 19/20 | 8.07ab±1.35 | 6.25abc±0.97 | 7.68a→d±1.25 | 6.05a→d±0.93 | 6.35h→l±0.92 | 5.36i→l±0.81 |
| SiNPs_200_ | Giza 171 | 5.97tuv±0.82 | 5.16stu±0.78 | 6.17p→s±0.87 | 5.26p→s±0.80 | 7.42b±1.18 | 5.92b±0.91 |
|  | Sakha 95 | 6.58l→q±0.97 | 5.47m→q±0.83 | 6.22pq±0.89 | 5.28pq±0.80 | 5.27rst±0.65 | 4.79st±0.72 |
|  | Misr 3 | 6.77j→n±1.01 | 5.58j→n±0.85 | 6.41nop±0.93 | 5.39op±0.81 | 5.76pq±0.76 | 5.05pq±0.76 |
|  | Gemmeiza-9 | 6.84j→m±1.04 | 5.61j→m±0.85 | 7.57a→g±1.23 | 5.99c→g±0.92 | 6.66gh±0.99 | 5.51h±0.83 |
|  | Giza-168 | 7.07ij±1.10 | 5.73j±0.87 | 7.22g→j±1.14 | 5.81hij±0.89 | 6.59hi±0.96 | 5.48hi±0.82 |
|  | Sids-14 | 7.97a→d±1.32 | 6.20a→d±0.96 | 7.93a±1.31 | 6.18a±0.95 | 7.14bcd±1.11 | 5.77bcd±0.88 |
|  | SOKOLL | 8.07ab±1.35 | 6.26ab±0.97 | 7.85abc±1.30 | 6.14abc±0.94 | 8.61a±1.49 | 6.54a±1.02 |
|  | 18 SAWYT 19/20 | 8.13a±1.37 | 6.28a±0.98 | 7.88ab±1.31 | 6.15ab±0.95 | 6.32h→m±0.91 | 5.34i→m±0.80 |
| The data of three replicates ± SD (standard deviation) are shown.  Means followed by different letters under the same water regimes were significantly different according to Duncan’s Multiple Range Test (p≤ 0.05) | | | | | | | |
